# Risk factors for neonatal mortality: an observational cohort study in Sarlahi district of rural southern Nepal

**DOI:** 10.1136/bmjopen-2022-066931

**Published:** 2023-09-14

**Authors:** Tingting Yan, Luke C Mullany, Seema Subedi, Elizabeth A Hazel, Subarna K Khatry, Diwakar Mohan, Scott Zeger, James M Tielsch, Steven C LeClerq, Joanne Katz

**Affiliations:** 1 Department of International Health, Johns Hopkins University Bloomberg School of Public Health, Baltimore, Maryland, USA; 2 Nepal Nutrition Intervention Project - Sarlahi (NNIPS), Nepal Eye Hospital Complex, Tripureshwor, Kathmandu, Nepal; 3 Department of Biostatistics, Johns Hopkins University Bloomberg School of Public Health, Baltimore, Maryland, USA; 4 Department of Global Health, George Washington University School of Public Health and Health Services, Washington, DC, USA

**Keywords:** Epidemiology, Public health, Risk Factors, Factor Analysis, Statistical, Primary Prevention, NEONATOLOGY

## Abstract

**Objectives:**

To assess the association between maternal characteristics, adverse birth outcomes (small-for-gestational-age (SGA) and/or preterm) and neonatal mortality in rural Nepal.

**Design:**

This is a secondary observational analysis to identify risk factors for neonatal mortality, using data from a randomised trial to assess the impact of newborn massage with different oils on neonatal mortality in Sarlahi district, Nepal.

**Setting:**

Rural Sarlahi district, Nepal.

**Participants:**

40 119 pregnant women enrolled from 9 September 2010 to 16 January 2017.

**Main outcome:**

The outcome variable is neonatal death. Cox regression was used to estimate adjusted Hazard Ratios (aHRs) to assess the association between adverse birth outcomes and neonatal mortality.

**Results:**

There were 32 004 live births and 998 neonatal deaths. SGA and/or preterm birth was strongly associated with increased neonatal mortality: SGA and preterm (aHR: 7.09, 95% CI: (4.44 to 11.31)), SGA and term/post-term (aHR: 2.12, 95% CI: (1.58 to 2.86)), appropriate-for-gestational-age/large-for-gestational-age and preterm (aHR: 3.23, 95% CI: (2.30 to 4.54)). Neonatal mortality was increased with a history of prior child deaths (aHR: 1.53, 95% CI: (1.24 to 1.87)), being a twin or triplet (aHR: 5.64, 95% CI: (4.25 to 7.48)), births at health posts/clinics or in hospital (aHR: 1.34, 95% CI: (1.13 to 1.58)) and on the way to facilities or outdoors (aHR: 2.26, 95% CI: (1.57 to 3.26)). Risk was lower with increasing maternal height from <145 cm to 145–150 cm (aHR: 0.78, 95% CI: (0.65 to 0.94)) to ≥150 cm (aHR: 0.57, 95% CI: (0.47 to 0.68)), four or more antenatal care (ANC) visits (aHR: 0.67, 95% CI: (0.53 to 0.86)) and education >5 years (aHR: 0.75, 95% CI: (0.62 to 0.92)).

**Conclusion:**

SGA and/or preterm birth are strongly associated with increased neonatal mortality. To reduce neonatal mortality, interventions that prevent SGA and preterm births by promoting ANC and facility delivery, and care of high-risk infants after birth should be tested.

**Trial registration number:**

NCT01177111.

**Strengths and limitations of this study**

- A large number of pregnancies followed with frequent visits in the neonatal period ensure a statistically powered analysis.
- The availability of a large amount of data for each participant enables a wide range of risk factor to be investigated.
- The date of last menstrual period was obtained early in pregnancy, limiting recall bias, and stillbirths were identified, but both could lead to some misclassification of preterm birth and neonatal death.
- Missing weights due to early newborn deaths or weighing of the baby 72 hours or more after birth were estimated by using multiple imputation, which increased the variability but reduced bias of risk estimates.

## Introduction

Globally, 2.3 million children died in the first 28 days of life in 2021, with the neonatal mortality rate of 18 deaths per 1000 live births.^1^ Almost 99% of neonatal deaths occur in low- and middle-income countries (LMICs).^2^ Neonatal mortality in Nepal declined from 50 per 1000 live births in 1996 to 21 in 2022,^2^ but neonatal deaths account for a higher percentage of under-5 child deaths since mortality rates among older children have decreased faster than neonatal mortality.^2 3^ If the trend continues, it would take another 50 years^4^ to reach the Sustainable Development Goals target of reducing neonatal mortality to 12 per 1000 live births by 2030.^5^

Although Nepal has continuously reformed its primary healthcare system and expanded the number of health facilities,^6^ there are still daunting obstacles hindering neonatal health, including lacking access to antenatal care (ANC), limited health infrastructure, poor transport and communication, inadequate affordability^7^ and significant discrepancy of Nepalese households’ access to health services between urban and rural areas.^8^ Studies have also shown numerous risk factors contributing to neonatal mortality.^9–15^ Small-for-gestational-age (SGA) and preterm births are associated with neonatal deaths in LMICs.^10^ Other factors include maternal age^11^ and education,^12^ household income,^13^ previous pregnancy history,^14^ tetanus vaccination,^15^ attendance at ANC^16^ and place of delivery.^17^

To identify relatively important ones and explore more evidence for interventions to reduce neonatal mortality in specific settings, we used data from the Nepal Oil Massage Study (NOMS), a community-based, cluster-randomised controlled trial in rural southern Nepal to conduct an observational analysis to assess the association between maternal characteristics, adverse birth outcomes (SGA and/or preterm) and other maternal and infant characteristics and neonatal mortality.

## Methods

### Study setting

This study was conducted in Sarlahi district of Southern Nepal, a poor rural area of subsistence farming in the low-lying plains of Nepal bordering Bihar, India. In Sarlahi district, neonatal mortality ranges from 30.0 to 41.9 deaths per 1000 live births, even higher than the average in Nepal^18^; 30% of births are <2500 g and over 92% of births occur at home. ANC in this setting is limited.^19^

### Study design

The NOMS was a cluster-randomised, community-based trial (Clinicaltrials.gov (NCT01177111)). The geographic area was divided into sectors that were randomised to receive promotion of full-body massage with mustard oil, which is the standard practice, or the intervention group that received promotion of full-body massage with sunflower seed oil. All women of childbearing age in the 34 participating Village Development Committees were eligible for the trial. Women who consented to pregnancy surveillance were visited every 5 weeks and asked whether they had their period since the last visit. If not, they were offered a pregnancy test. If positive, they were enrolled in the trial. Women were followed with monthly visits until delivery. A birth assessment was conducted on the first visit to the newborn baby (the day of birth or as soon after birth as possible), and neonates were followed up on 1, 3, 7, 10, 14, 21 and 28 days of age. Vital status was collected on mothers and infants at each of these time points. A verbal autopsy was conducted on the causes of neonatal death and the date of death was also recorded. For the analysis of risk factors for neonatal mortality, only live born infants and their mothers were included.

### Data collection

Data for the trial were collected from November 2010 through January 2017. All data collectors were employed and trained by the study. This included local village women who collected data from the pregnant women in their communities. Data such as anthropometry were collected by study employees trained and standardised to collect these measurements. For more complex survey data and supervisory staff, local people with prior experience collecting these types of data were employed. At the time women were identified as pregnant and enrolled, date of last menstrual period, maternal age, height, education and reproductive history were recorded. Information about household assets, caste and ethnicity of the family was also collected at the time of enrolment. During pregnancy, baseline and monthly visits in pregnancy recorded receipt of tetanus vaccination, tobacco and alcohol use and maternal morbidity. In the first visit within 72 hours after birth, late pregnancy morbidity, labour and delivery characteristics and immediate newborn care practices were collected. Neonatal characteristics included sex, weight and the time since birth at which weight was measured. The aim was to measure weight as soon after birth as possible.

### Patient and public involvement

Our study was an institutional review board-approved secondary analysis of a pre-existing dataset generated from the NOMS trial. Therefore, it was not appropriate to involve patients or the public in the design, conduct, reporting, or dissemination plans for this research.

### Definitions of variables

Neonatal death was defined as the death of a live-born baby in the first 28 days of life and the neonatal mortality rate was defined as the number of neonatal deaths per 1000 live births.^20^ Low birth weight (LBW) was defined as a birth weight less than 2500 g.^21^ Birth weight was also divided into four groups: <1500 g as very low birth weight (VLBW), 1500–<2500 g as moderately LBW, 2500–4000 g (4000 g included) as normal birth weight (NBW), >4000 g as high birth weight.^22^ For our analysis, it was also categorised into two groups: LBW (<2500 g) and NBW (≥2500 g). Gestational age was calculated by taking the number of days between pregnancy outcome and date of last menstrual period obtained by recall at enrolment in the trial.^23^ Preterm births were babies born alive before 37 completed weeks gestation.^24^ Gestational age was also categorised into four groups: <32 weeks, 32 to <37 weeks, 37 to <42 weeks and 42–45 weeks, and labelled as very preterm, moderate to late preterm, term and post-term, respectively.^25 26^ For our analysis, it was also categorised into two groups: preterm (<37 weeks) and term (37–45 weeks). SGA was defined as newborns with weight below the 10th percentile of newborns using the Intergrowth reference population.^27^ Appropriate-for-gestational-age (AGA) was defined as weight between the 10th and 90th percentiles of the reference population, and large-for-gestational-age (LGA) was defined as a weight above the 90th percentile of the reference population.^27^ Parity was defined as the number of prior pregnancies resulting in a live or stillbirth. Gravidity was the number of prior pregnancies, regardless of the outcome of the pregnancy.^28^

### Data analysis

The data described above were managed and analysed using Stata V.16.0.^29^ Cumulative mortality curves (the inverse of Kaplan-Meier survival curves) (cumulative mortality) were used to describe the differences in neonatal mortality by SGA and preterm status specifically for four groups: (SGA and preterm, SGA and term/post-term, AGA/LGA and preterm, and AGA/LGA and term/post-term).

Maternal characteristics included demographics, socioeconomic status, lifestyle and reproductive history. Maternal age in pregnancy was categorised as ≤18 years, 18 years to 35 years and >35 years. Maternal height was categorised as <145 cm, 145 cm to <150 cm and ≥150 cm. Socioeconomic information included years of education (no schooling as reference, 1 year to ≤5 years and >5 years), and wealth quintiles based on a standardised score of the total number of household assets including land, animal, transport and mobile phone ownership^30^ and caste of the family (Brahmin and Chhetri, Vaishya, Shudra and Muslim). Reproductive information included parity (prior pregnancy but no parity, no prior pregnancy, 1–4 and ≥5), gravidity (0 and ≥1), last pregnancy outcome (no prior pregnancy, at least one live birth, stillbirth, or miscarriage or abortion), a history of child deaths, any prior pregnancy that ended in stillbirth or miscarriage, and if the current pregnancy was a multiple birth (twins or triplets). Maternal exposures in pregnancy included self-reported tobacco and/or alcohol use.

For healthcare in pregnancy and characteristics of delivery, tetanus vaccination receipt, place of delivery (at home or maternal home, at a health post/clinic or in hospital, or on the way to the facility or outdoors) and the number of ANC visits (0, 1, 2 or 3, ≥4) were examined.

Neonatal characteristics included birth weight (taken within 72 hours of birth), gestational age, sex, size for gestational age and singleton/twin/triplet in our analysis. Because weights were taken at varying times after birth, and birth weight was missing for some infants who died very soon after birth or was measured more than 72 hours after birth, we multiply imputed birth weights (and hence SGA percentiles) for those with missing weights (601/3957, 15.2% missing) and also imputed weight at time zero for all those with weights and times of weight measurement. This was done using an empirical Bayes regression model of early neonatal weight change by estimating, then recalibrating from the conditional distribution of each child’s birth weight given a single measurement at a known later time or imputing given missing weight based on longitudinal daily weights from day of birth through 10 days in a population of infants from the same study area as this trial.^31^

We did not include the intervention as a covariate in the regression model because the results of the randomised trial are being written up for publication but are not yet citable. However, there was no significant difference in mortality between the intervention and control groups. Hence, not including this variable in the regression should not impact the results presented here.

For estimating HRs, we used Cox regression. The outcome was defined as time from birth to death or time the baby was last seen alive. This analysis takes account of loss to follow-up and variable ages at death.

In the simple Cox regression models, we calculated the crude HR (cHR) and their 95% CI for each covariate. In multivariable regression, we did not include some variables because of collinearity. For example, we did not include both parity and gravidity. We did not include prior miscarriage or stillbirth because we included prior child deaths. We developed four different models to examine the adjusted HRs (aHRs) and their 95% CIs for risk factors. In model 1, we included maternal age, height, years of education, wealth quintile, caste, parity, prior child deaths, tetanus vaccination, place of delivery, number of ANC visits, sex of the infant, preterm, adverse birth outcomes (SGA and preterm, SGA and term/post-term, AGA/LGA and preterm, and AGA/LGA and term/post-term) and singleton/twin/triplet. In model 2, we included all the variables in model 1, except place of delivery and number of ANC visits. We ran the models with and without these two variables because about 10% of the population was missing these variables and we wanted to see whether excluding these variables produced similar regression results for the predictors of interest to when they were included. For models 3 and 4 (in supplemental materials), we included gestational age and size for gestation separately, to compare with models 1 and 2, respectively. We ran each of these four models twice, one time where we used weights and associated SGA/preterm taken within 72 hours of birth, and again using the multiply imputed weights and imputed SGA. This allowed us to compare how the imputation impacted the regression coefficients. Since some women had more than one pregnancy included in the analysis, we accounted for correlation within women using robust variance estimation.^32^

For multivariable Cox regression, we have assessed the fitness of each of the four models using Stata and found that none indicated a significant deviation from the proportional hazard assumption.

## Results

**Figure 1:**
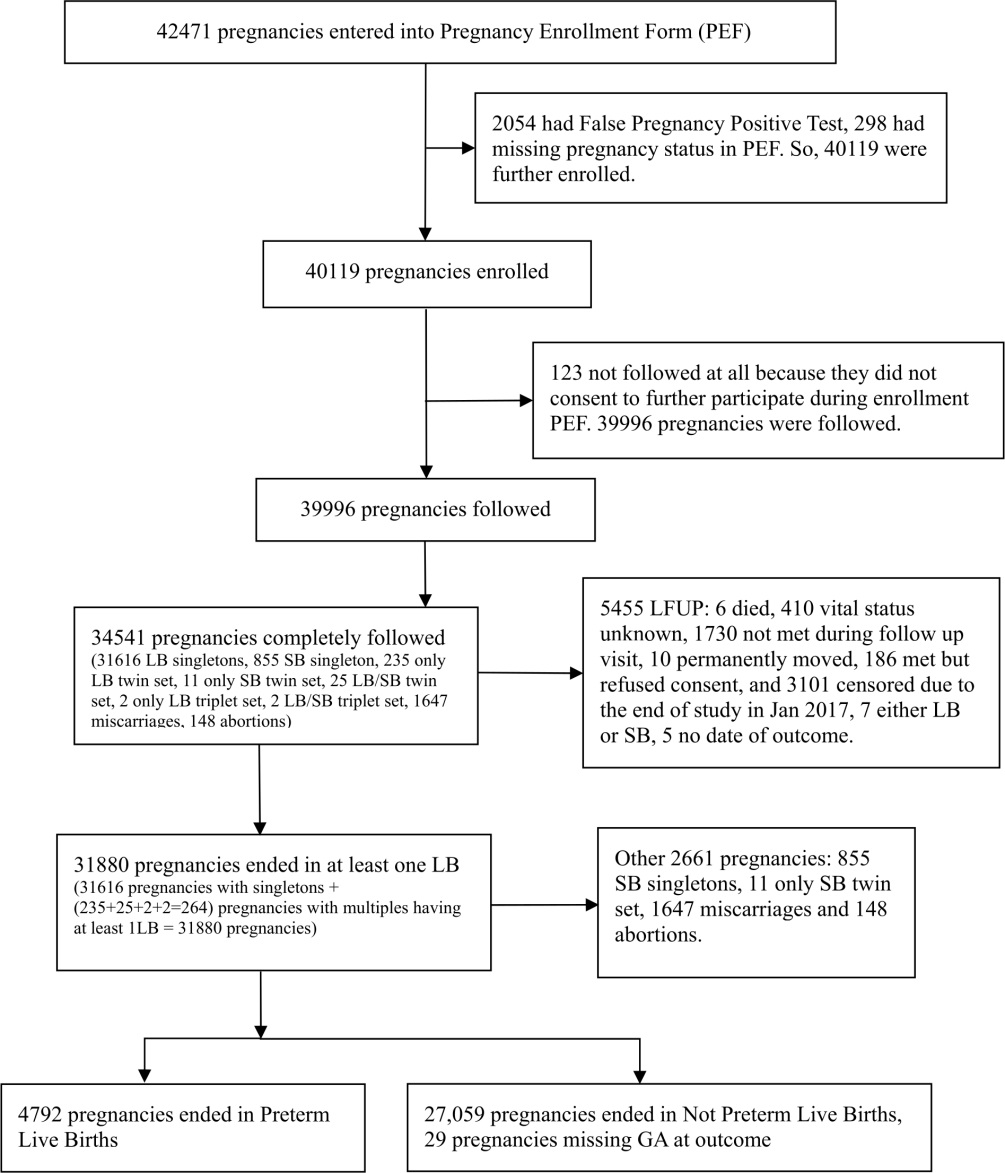
Flow diagram for participants in Nepal Oil Massage Study. *Data collection was halted for 6 weeks in December 2016. LB, Livebirth; SB, Stillbirth; GA, Gestational Age; LFUP, Lost to Follow Up.

A total of 32 004 live-born babies were included in this analysis. Figure 1 shows the number of pregnancies identified, enrolled and their outcomes (not including imputed birth weight). The baseline characteristics, neonatal mortality and cHR of each potential risk factor are shown in table 1. Most pregnant women were between ages 18 years and 35 years. Fifteen per cent of women were shorter than 145 cm and 67% had no schooling. Twenty-nine per cent had no prior pregnancy and almost no women reported the use of alcohol or tobacco during pregnancy (table 1). While 42% of women delivered in a facility, 97% of births were vaginal deliveries.

**Table 1:** Characteristics, neonatal mortality and crude HRs of potential risk factors (n=32 004)

Variables	Study population (n=32 004)			Neonatal mortality (per 1000)	Crude HR	95% CI
	Deaths	Total	Distribution %			
Maternal age (years) at LMP						
Mean, 95% CI: 22.46 (22.41 to 22.51)						
≤18	218	4948	15.46	44.06	1.57	(1.34 to 1.83)
18–35	748	26 351	82.34	28.39	1.00	Reference
>35	32	703	2.20	45.52	1.62	(1.14 to 2.31)
Missing	0	2		0.00		
Maternal height (cm)						
Mean, 95% CI: 150.55 (150.49 to 150.61)						
<145	232	4703	14.72	49.33	1.00	Reference
145–150	332	9587	30.00	34.64	0.70	(0.59 to 0.83)
≥150	429	17 664	55.28	24.29	0.49	(0.41 to 0.57)
Missing	5	50		100.00		
Maternal education (years)						
Mean, 95% CI: 2.61 (2.56 to 2.65)						
No schooling	748	21 557	67.42	34.70	1.00	Reference
1–5	77	2721	8.51	28.30	0.81	(0.64 to 1.03)
>5	173	7695	24.07	22.48	0.64	(0.54 to 0.76)
Missing	0	31		0.00		
Wealth quintile						
Poorest	262	6534	20.43	40.10	1.00	Reference
Poorer	223	6407	20.03	34.81	0.87	(0.72 to 1.05)
Middle	206	6400	20.01	32.19	0.80	(0.66 to 0.97)
Richer	154	6303	19.71	24.43	0.60	(0.49 to 0.74)
Richest	152	6339	19.82	23.98	0.59	(0.48 to 0.73)
Missing	1	21		47.62		
Caste of the family						
Brahmin and Chhetri	26	965	3.02	26.94	1.00	Reference
Vaishya	682	23 062	72.13	29.57	1.10	(0.74 to 1.63)
Shudra	208	4947	15.47	42.05	1.57	(1.05 to 2.37)
Muslim and others	81	2999	9.38	27.01	1.01	(0.65 to 1.57)
Missing	1	31		32.26		
Parity						
1–4	523	20 444	64.22	25.58	1.00	Reference
≥5	62	1398	4.39	44.35	1.75	(1.33 to 2.30)
Prior pregnancy but no parity	35	791	2.48	44.25	1.75	(1.24 to 2.47)
No prior pregnancy	369	9199	28.90	40.11	1.58	(1.38 to 1.82)
Missing	9	172		52.33		
Gravidity						
≥1	629	22 804	71.26	27.58	1.00	Reference
0 (First pregnancy)	369	9199	28.74	40.11	1.46	(1.28 to 1.67)
Missing	0	1		0.00		
Last pregnancy outcome						
No prior pregnancy	369	9199	28.76	40.11	1.53	(1.34 to 1.76)
At least one live birth	535	20 284	63.43	26.38	1.00	Reference
Stillbirth	19	532	1.66	35.71	1.36	(0.86 to 2.14)
Miscarriage and abortion	75	1965	6.14	38.17	1.46	(1.13 to 1.89)
Missing	0	24		0.00		
Prior child deaths						
No prior pregnancy	369	9199	29.19	40.11	1.76	(1.52 to 2.04)
Prior live births but no child deaths	405	17 597	55.83	23.02	1.00	Reference
Prior live births and child death	149	3641	11.55	40.92	1.80	(1.48 to 2.18)
Prior pregnancy but no live birth	45	1081	3.43	41.63	1.83	(1.33 to 2.53)
Missing	30	486		61.73		
Any prior stillbirth						
No prior pregnancy	369	9199	28.76	40.11	1.52	(1.32 to 1.74)
Prior pregnancy but no still births	571	21 394	66.88	26.69	1.00	Reference
Prior still births	58	1394	4.36	41.61	1.58	(1.18 to 2.10)
Missing	0	17		0.00		
Any prior miscarriage						
No prior pregnancy	369	9199	28.75	40.11	1.54	(1.34 to 1.77)
Prior pregnancy but no miscarriage	503	19 142	59.83	26.28	1.00	Reference
Prior miscarriage	126	3652	11.41	34.50	1.32	(1.08 to 1.62)
Missing	0	11		0.00		
Any prior multiple births						
No prior pregnancy	369	9199	28.76	40.11	1.49	(1.30 to 1.70)
Prior pregnancy but no multiples	611	22 484	70.30	27.17	1.00	Reference
Prior multiples	18	299	0.93	60.20	2.25	(1.38 to 3.68)
Missing	0	22		0.00		
Tetanus vaccination in the past 2 years						
No	193	5066	15.83	38.10	1.00	Reference
Yes	805	26 938	84.17	29.88	0.78	(0.66 to 0.92)
Missing	0	0				
Any tobacco use in pregnancy						
No	987	31 649	98.89	31.19	1.00	Reference
Yes	11	355	1.11	30.99	0.99	(0.55 to 1.80)
Missing	0	0				
Any alcohol use in pregnancy						
No	997	31 908	99.70	31.34	1.00	Reference
Yes	1	96	0.30	10.42	0.33	(0.05 to 2.37)
Missing	0	0				
Place of delivery						
At home or Maiti*	437	15 872	55.46	27.53	1.00	Reference
At health post/clinic or in hospital	405	12 130	42.38	33.39	1.22	(1.06 to 1.40)
On the way to facility or outdoors	40	617	2.16	64.83	2.43	(1.70 to 3.48)
Missing	116	3385		34.27		
Number of ANC visits						
0	184	5559	19.46	33.10	1.00	Reference
1	148	4173	14.61	35.47	1.07	(0.86 to 1.34)
2 or 3	366	9872	34.55	37.07	1.12	(0.93 to 1.35)
≥4	183	8967	31.38	20.41	0.61	(0.50 to 0.76)
Missing	117	3433		34.08		
Intervention						
Mustard oil	520	16 327	51.02	31.85	1.00	Reference
Sunflower oil	478	15 676	48.98	30.49	0.95	(0.84 to 1.09)
Missing	0	1		0		
Sex of child						
Male	548	16 531	51.73	26.63	1.00	Reference
Female	449	15 424	48.27	29.91	0.88	(0.77 to 0.99)
Missing	1	49		20.41		
Birth weight (within 72 hours)						
Mean, 95% CI: 2773.19 g (2766.95 to 2779.44)						
VLBW (<1500 g)	51	128	0.46	398.44	69.88	(49.58 to 98.50)
LBW (1500–2500 g)	199	7859	28.02	25.32	3.43	(2.76 to 4.26)
NBW (2500–4000 g)	141	19 354	69.01	7.29	1.00	Reference
HBW (>4000 g)	3	706	2.52	4.25	0.57	(0.18 to 1.78)
Missing	601	3957		151.88		
Imputed birth weight						
Mean, 95% CI: 2774.9 g (2770.1 to 2779.8)						
VLBW (<1500 g)	65	158	0.00	412.98	25.94	(19.38 to 34.73)
LBW (1500–2500 g)	491	8298	26.00	59.22	3.23	(2.65 to 3.94)
NBW (2500–4000 g)	437	23 355	73.17	18.69	1.00	Reference
HBW (>4000 g)	4	110	0.00	32.72	1.74	(0.59 to 5.14)
Missing	1	83		12.05		
Birth weight (within 72 hours)						
LBW (<2500 g)	250	7987	28.48	31.30	4.32	(3.51 to 5.33)
NBW (≥2500 g)	147	20 060	71.52	7.33	1.00	Reference
Missing	601	3957		151.88		
Imputed birth weight						
LBW (<2500 g)	556	8456	26.49	65.77	3.59	(2.99 to 4.32)
NBW (≥2500 g)	440	23 465	73.51	18.76	1.00	Reference
Missing	1	83		12.05		
Gestational age (days)						
Mean, 95% CI: 275.74 (275.49 to 275.99)						
Very preterm (<32 weeks)	193	662	2.12	291.54	17.44	(14.54 to 20.91)
Moderate to late preterm (32–37 weeks)	224	4244	13.62	52.78	2.66	(2.25 to 3.14)
Term (37–42 weeks)	451	22 371	71.78	20.16	1.00	Reference
Post-term (42–45 weeks)	111	3887	12.47	28.56	1.42	(1.16 to 1.75)
Missing	0	3		0.00		
Gestational age						
Preterm (<37 weeks)	417	4906	15.74	85.00	4.12	(3.60 to 4.71)
Term (37–45 weeks)	562	26 258	84.26	21.40	1.00	Reference
Missing	0	3		0.00		
Size for gestational age (within 72 hours)						
AGA	140	13 172	47.01	10.69	1.00	Reference
SGA	244	13 060	46.61	18.54	1.76	(1.42 to 2.18)
LGA	12	1789	6.38	6.83	0.63	(0.35 to 1.12)
Missing	602	3983		148.36		
Imputed size for gestational age						
AGA	373	16 127	50.57	23.15	1.00	Reference
SGA	471	14 275	44.77	32.96	1.43	(1.10 to 1.87)
LGA	147	1486	4.66	98.95	4.52	(3.49 to 5.85)
Missing	7	116		60.34		
SGA/preterm (within 72 hours)						
AGA/LGA and term/post-term	70	11 381	41.64	6.15	1.00	Reference
SGA and term/post-term	185	11 859	43.39	15.60	2.55	(1.93 to 3.37)
AGA/LGA and preterm	81	3437	12.58	23.57	3.86	(2.76 to 5.39)
SGA and preterm	54	652	2.39	82.82	13.95	(9.67 to 20.13)
Missing	608	4675		130.05		
Imputed SGA/preterm						
AGA/LGA and term/post-term	189	13 413	43.42	14.06	1.00	Reference
SGA and term/post-term	391	13 607	41.32	28.76	2.06	(1.50 to 2.83)
AGA/LGA and preterm	324	4175	13.30	77.55	5.73	(4.56 to 7.21)
SGA and preterm	79	669	1.97	118.38	8.82	(5.85 to 13.28)
Missing	15	140		60.34		
Singleton/twin/triplet						
Singleton	892	31 502	98.43	28.32	1.00	Reference
Twin or triplet	106	502	1.57	211.16	8.31	(6.43 to 10.74)
Missing	0	0				

*Maiti is the maternal home, where women may go to deliver, especially in the first pregnancy.

AGA, appropriate-for-gestational-age; ANC, antenatal care; HBW, high birth weight; LBW, low birth weight; LGA, large-for-gestational-age; LMP, Last Menstrual Period; NBW, normal birth weight; SGA, small-for-gestational-age; VLBW, very low birth weight.

Based on the cHRs, maternal height, education, wealth quintile, caste, parity, gravidity, prior child deaths and prior adverse pregnancy outcomes, place of delivery, number of ANC visits, multiple births, birth weight, SGA and preterm were all significantly associated with neonatal mortality. Birth weight imputation altered the cHRs for high birth weight (>4000 g) from being a risk factor to being protective and LGA (>90th percentile) from no association to being a significant risk factor. While being SGA and preterm had a large and significant HR in the analysis without imputed weight values, this HR increased significantly when birth weights were imputed.

Variables with high proportions of missing data (around 10%) were ANC visits (10.7%), place of delivery (10.6%), birth weight (12.4%) and weights taken after 72 hours (13.5%). Neonatal mortality was higher among women who delivered at a facility or on their way to a facility compared with home births. Women who attended four or more ANC visits had lower neonatal mortality risk. Both before and after imputation, VLBW and LBW significantly increased neonatal mortality. Preterm or post-term resulted in higher neonatal mortality. SGA and LGA babies were at higher risk compared with AGA ones. Alcohol and tobacco use was not included in multivariate models, given the low prevalence of their use in pregnancy.

Multivariable regression results are shown in tables 2 and 3. We calculated cHRs for every category for individual variables. If 95% CIs of cHRs covered 1.00, we did not include the variable in our models. These models did not include gravidity, last pregnancy outcome, any prior pregnancy ending in stillbirth, miscarriage or multiples, and tobacco and alcohol use. Model 1 included place of delivery and number of ANC visits, while model 2 did not.

**Table 2:** Multivariate Cox regression model 1

Variables	Crude HR	95% CI	Model 1: maternal age, mother’s height, mother’s years of education, wealth quintile, caste, parity, prior child deaths, tetanus vaccination, place of delivery, number of ANC visits, sex of child, SGA/preterm, imputed SGA/preterm, singleton/twin+triplet			
			Adjusted HR (n=26 626)	95% CI	Adjusted HR (using imputed SGA) (n=27 819)	95% CI
Maternal age (years) at LMP						
Mean, 95% CI: 22.46 (22.41 to 22.51)						
≤18	1.57	(1.34 to 1.83)	1.39	(1.04 to 1.86)	1.22	(1.01 to 1.50)
18–35	1.00	Reference	1.00	Reference	1.00	Reference
>35	1.62	(1.14 to 2.31)	1.28	(0.60 to 2.77)	1.36	(0.84 to 2.21)
Maternal height (cm)						
Mean, 95% CI: 150.55 (150.49 to 150.61)						
<145	1.00	Reference	1.00	Reference	1.00	Reference
145–150	0.70	(0.59 to 0.83)	0.64	(0.48 to 0.86)	0.75	(0.62 to 0.92)
150	0.49	(0.41 to 0.57)	0.57	(0.43 to 0.75)	0.55	(0.46 to 0.67)
Maternal education (years)						
Mean, 95% CI: 2.61 (2.56 to 2.65)						
No schooling	1.00	Reference	1.00	Reference	1.00	Reference
1–5	0.81	(0.64 to 1.03)	1.01	(0.69 to 1.49)	0.92	(0.71 to 1.20)
>5	0.64	(0.54 to 0.76)	0.79	(0.57 to 1.09)	0.78	(0.62 to 0.97)
Wealth quintile						
Poorest	1.00	Reference	1.00	Reference	1.00	Reference
Poorer	0.87	(0.72 to 1.05)	0.85	(0.61 to 1.17)	0.92	(0.74 to 1.13)
Middle	0.80	(0.66 to 0.97)	0.96	(0.69 to 1.33)	0.95	(0.76 to 1.19)
Richer	0.60	(0.49 to 0.74)	0.73	(0.50 to 1.06)	0.82	(0.64 to 1.05)
Richest	0.59	(0.48 to 0.73)	0.89	(0.60 to 1.32)	0.91	(0.70 to 1.18)
Caste of the family						
Brahmin and Chhetri	1.00	Reference	1.00	Reference	1.00	Reference
Vaishya	1.10	(0.74 to 1.63)	1.29	(0.57 to 2.91)	0.86	(0.55 to 1.34)
Shudra	1.57	(1.05 to 2.37)	1.61	(0.69 to 3.76)	1.01	(0.63 to 1.64)
Muslim and others	1.01	(0.65 to 1.57)	1.04	(0.43 to 2.52)	0.69	(0.41 to 1.14)
Parity						
1–4	1.00	Reference	1.00	Reference	1.00	Reference
≥5	1.75	(1.33 to 2.30)	1.20	(0.66 to 2.19)	1.04	(0.70 to 1.55)
Prior pregnancy but no parity	1.75	(1.24 to 2.47)	1.99	(0.43 to 9.11)	1.63	(0.69 to 3.84)
No prior pregnancy	1.58	(1.38 to 1.82)	1.43	(1.06 to 1.92)	1.73	(1.42 to 2.12)
Prior child deaths						
No prior pregnancy	1.76	(1.52 to 2.04)	Omitted		Omitted	
Prior live births but no child deaths	1.00	Reference	1.00	Reference	1.00	Reference
Prior live births and child death	1.80	(1.48 to 2.18)	1.11	(0.79 to 1.55)	1.56	(1.26 to 1.95)
Prior pregnancy but no live birth	1.83	(1.33 to 2.53)	0.71	(0.17 to 2.93)	1.28	(0.57 to 2.88)
Tetanus vaccination in the past 2 years						
No	1.00	Reference	1.00	Reference	1.00	Reference
Yes	0.78	(0.66 to 0.92)	0.84	(0.63 to 1.11)	0.74	(0.62 to 0.89)
Place of delivery						
At home or Maiti*	1.00	Reference	1.00	Reference	1.00	Reference
At health post/clinic or in hospital	1.22	(1.06 to 1.40)	0.94	(0.73 to 1.21)	1.34	(1.13 to 1.58)
On the way to facility or outdoors	2.43	(1.70 to 3.48)	1.42	(0.72 to 2.81)	2.26	(1.57 to 3.26)
Number of ANC						
0	1.00	Reference	1.00	Reference	1.00	Reference
1	1.07	(0.86 to 1.34)	1.03	(0.73 to 1.47)	1.07	(0.85 to 1.34)
2 or 3	1.12	(0.93 to 1.35)	1.06	(0.79 to 1.42)	1.06	(0.87 to 1.29)
≥4	0.61	(0.50 to 0.76)	0.77	(0.54 to 1.11)	0.67	(0.53 to 0.86)
Sex of child						
Male	1.00	Reference	1.00	Reference	1.00	Reference
Female	0.88	(0.77 to 0.99)	1.16	(0.94 to 1.43)	0.92	(0.80 to 1.06)
SGA/preterm (within 72 hours)						
AGA/LGA and term/post-term	1.00	Reference	1.00	Reference		
SGA and term/post-term	2.55	(1.93 to 3.37)	2.11	(1.56 to 2.83)		
AGA/LGA and preterm	3.86	(2.76 to 5.39)	3.11	(2.22 to 4.35)		
SGA and preterm	13.95	(9.67 to 20.13)	6.90	(4.33 to 10.97)		
Imputed SGA/preterm						
AGA/LGA and term/post-term	1.00	Reference			1.00	Reference
SGA and term/post-term	2.06	(1.50 to 2.83)			1.67	(1.10 to 2.54)
AGA/LGA and preterm	5.73	(4.56 to 7.21)			4.26	(3.20 to 5.66)
SGA and preterm	8.82	(5.85 to 13.28)			3.92	(2.18 to 7.07)
Singleton/twin/triplet						
Singleton	1.00	Reference	1.00	Reference	1.00	Reference
Twin or triplet	8.31	(6.43 to 10.74)	4.89	(2.91 to 8.21)	5.43	(4.01 to 7.37)

*Maiti is the maternal home, where women may go to deliver, especially in the first pregnancy.

AGA, appropriate-for-gestational-age; ANC, antenatal care; LGA, large-for-gestational-age; LMP, Last Menstrual Period; SGA, small-for-gestational-age.

**Table 3:** Multivariate Cox regression model 2

Variables	Crude HR	95% CI	Model 2: maternal age, mother’s height, mother’s years of education, wealth quintile, caste, parity, prior child death, tetanus vaccination, sex of child, SGA/preterm, imputed SGA/preterm, singleton/twin/triplet			
			Adjusted HR (n=26 680)	95% CI	Adjusted HR (using imputed SGA) (n=31 116)	95% CI
Maternal age (years) at LMP						
Mean, 95% CI: 22.46 (22.41 to 22.51)						
≤18	1.57	(1.34 to 1.83)	1.41	(1.05 to 1.88)	1.15	(0.95, to 1.38)
18–35	1.00	Reference	1.00	Reference	1.00	Reference
>35	1.62	(1.14 to 2.31)	1.26	(0.58 to 2.71)	1.46	(0.93 to 2.28)
Maternal height (cm)						
Mean, 95% CI: 150.55 (150.49 to 150.61)						
<145	1.00	Reference	1.00	Reference	1.00	Reference
145–150	0.70	(0.59 to 0.83)	0.64	(0.48 to 0.85)	0.78	(0.65 to 0.94)
≥150	0.49	(0.41 to 0.57)	0.57	(0.43 to 0.75)	0.57	(0.47 to 0.68)
Maternal education (years)						
Mean, 95% CI: 2.61 (2.56 to 2.65)						
No schooling	1.00	Reference	1.00	Reference	1.00	Reference
1–5	0.81	(0.64 to 1.03)	0.98	(0.67 to 1.44)	0.89	(0.69 to 1.14)
>5	0.64	(0.54 to 0.76)	0.75	(0.55 to 1.04)	0.75	(0.62 to 0.92)
Wealth quintile						
Poorest	1.00	Reference	1.00	Reference	1.00	Reference
Poorer	0.87	(0.72 to 1.05)	0.84	(0.61 to 1.17)	0.96	(0.79 to 1.18)
Middle	0.80	(0.66 to 0.97)	0.94	(0.68 to 1.30)	0.94	(0.76 to 1.16)
Richer	0.60	(0.49 to 0.74)	0.71	(0.49 to 1.03)	0.82	(0.66 to 1.04)
Richest	0.59	(0.48 to 0.73)	0.87	(0.59 to 1.29)	0.92	(0.72 to 1.17)
Caste of the family						
Brahmin and Chhetri	1.00	Reference	1.00	Reference	1.00	Reference
Vaishya	1.10	(0.74 to 1.63)	1.34	(0.59 to 3.03)	0.86	(0.57 to 1.31)
Shudra	1.57	(1.05 to 2.37)	1.68	(0.72 to 3.93)	1.00	(0.64 to 1.57)
Muslim and others	1.01	(0.65 to 1.57)	1.08	(0.44 to 2.62)	0.67	(0.41 to 1.07)
Parity						
1–4	1.00	Reference	1.00	Reference	1.00	Reference
≥5	1.75	(1.33 to 2.30)	1.21	(0.67 to 2.21)	1.05	(0.72 to 1.52)
Prior pregnancy but no parity	1.75	(1.24 to 2.47)	2.01	(0.44 to 9.24)	1.52	(0.71 to 3.27)
No prior pregnancy	1.58	(1.38 to 1.82)	1.39	(1.05 to 1.85)	1.79	(1.50 to 2.15)
Prior child deaths						
No prior pregnancy	1.76	(1.52 to 2.04)	Omitted		Omitted	
Prior live births but no child death	1.00	Reference	1.00	Reference	1.00	Reference
Prior pregnancy but no live births	1.80	(1.48 to 2.18)	0.67	(0.16 to 2.77)	1.32	(0.65 to 2.67)
Prior live births and child death	1.83	(1.33 to 2.53)	1.11	(0.79 to 1.56)	1.53	(1.24 to 1.87)
Tetanus vaccination in the past 2 years						
No	1.00	Reference	1.00	Reference	1.00	Reference
Yes	0.78	(0.66 to 0.92)	0.84	(0.63 to 1.10)	0.88	(0.74 to 1.04)
Sex of child						
Male	1.00	Reference	1.00	Reference	1.00	Reference
Female	0.88	(0.77 to 0.99)	1.17	(0.95 to 1.45)	0.89	(0.78 to 1.01)
SGA/preterm (within 72 hours)						
AGA/LGA and term/post-term	1.00	Reference	1.00	Reference		
SGA and term/post-term	2.55	(1.93 to 3.37)	2.12	(1.58 to 2.86)		
AGA/LGA and preterm	3.86	(2.76 to 5.39)	3.23	(2.30 to 4.54)		
SGA and preterm	13.95	(9.67 to 20.13)	7.09	(4.44 to 11.31)		
Imputed SGA/preterm						
AGA/LGA and term/post-term	1.00	Reference			1.00	Reference
SGA and term/post-term	2.06	(1.50 to 2.83)			1.68	(1.20 to 2.35)
AGA/LGA and preterm	5.73	(4.56 to 7.21)			4.50	(3.51 to 5.77)
SGA and preterm	8.82	(5.85 to 13.28)			4.11	(3.52 to 6.69)
Singleton/twin/triplet						
Singleton	1.00	Reference	1.00	Reference	1.00	Reference
Twin or triplet	8.31	(6.43 to 10.74)	4.76	(2.84 to 8.00)	5.64	(4.25 to 7.48)

AGA, appropriate-for-gestational-age; LGA, large-for-gestational-age; LMP, Last Menstrual Period; SGA, small-for-gestational-age.

The total number of live births in the analysis was 26 626 with original data (weights taken within 72 hours), and 27 819 with imputed birth weight and SGA data in model 1 (table 2). Place of delivery had a significant impact on neonatal mortality: the risks were higher both at health posts/clinics or in hospital (aHR: 1.34, 95% CI: (1.13 to 1.58)) and on the way to the facility or outdoors (aHR: 2.26, 95% CI: (1.57 to 3.26)); the number of ANC visits was statistically significant for four or more visits (aHR: 0.67, 95% CI: (0.53 to 0.86)).

In model 2 (table 3), the number of participants included was 26 680 with weights taken within 72 hours, and 31 116 after birth weight imputation. The difference was due to more than 10% missing values for place of delivery and number of ANC visits, which were included in model 1, but not in model 2. Results for models 1 and 2 are quite similar, indicating that exclusion of place of delivery and ANC did not change the associations of the other risk factors with mortality.

After imputation and adjustment, variables such as maternal age, wealth quintile and caste of the family were not significantly associated with neonatal mortality. Increasing maternal height from <145 cm to 145–150 cm (aHR: 0.78, 95% CI: (0.65 to 0.94)) to ≥150 cm (aHR: 0.57, 95% CI: (0.47 to 0.68)) and education to >5 years (aHR: 0.75, 95% CI: (0.62 to 0.92)) remained protective factors. Parity was not significantly associated with neonatal mortality. Prior live births and child death (aHR: 1.53, 95% CI: (1.24 to 1.87)) were associated with higher risk. Tetanus vaccination did not have a significant impact on neonatal mortality. Being a twin or triplet (aHR: 5.64, 95% CI: (4.25 to 7.48)) was also significantly associated with neonatal mortality. The findings were similar in models 1 and 2.

**Figure 2:**
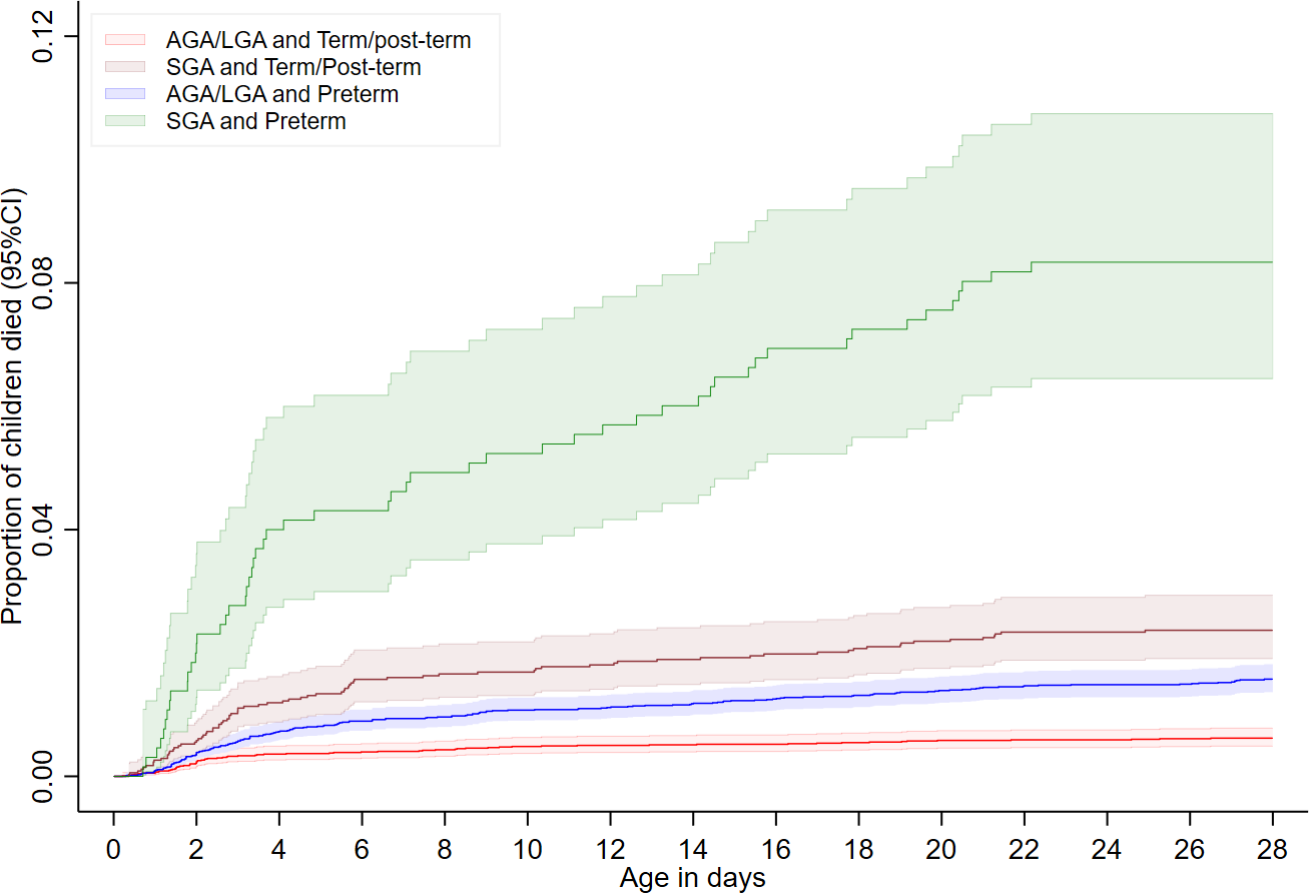
Cumulative neonatal mortality curves by SGA/preterm excluding imputed data. AGA, appropriate-for-gestational-age; LGA, large-for-gestational-age; SGA, small-for-gestational-age.

Any SGA and/or preterm was strongly associated with an increased likelihood of neonatal mortality (figure 2): SGA and preterm (aHR: 7.09, 95% CI: (4.44 to 11.31)), SGA and term/post-term (aHR: 2.12, 95% CI: (1.58 to 2.86)), AGA/LGA and preterm (aHR: 3.23, 95% CI: (2.30 to 4.54)). Imputation of birth weights increased the association between AGA/LGA and preterm and mortality but not the other categories of adverse birth outcomes.

We also created two other multivariate regression models, models S1 and S2 as shown in online supplemental tables S1 and S2 in the appendices. These included gestational age and size for gestational age as individual variables.

10.1136/bmjopen-2022-066931.supp1 Supplementary data

## Discussion

Nepal was one of the few countries to meet the Millennium Development Goal 4 for child mortality but neonatal mortality rates are still high and need to be reduced by twofold to meet the Sustainable Development Goals of 12 per 1000 live births.^33 34^ This study identified maternal characteristics (maternal height, education and prior child death), neonatal characteristics (SGA, preterm and twin or triplet), delivery and healthcare (place of delivery and number of ANC visits) as significant risk factors for neonatal mortality in Sarlahi district, rural southern Nepal.

Compared with previous existing studies, maternal age,^11 35 36^ maternal height,^37^ maternal education,^12 13^ wealth,^13 35^ LBW,^36 38 39^ SGA and preterm,^36 40–42^ twins^13 40 43^ and triplets^43^ were all strongly associated with neonatal deaths, which correspond to our findings. Previous studies suggest that parity was not considered a predictor of neonatal mortality,^44^ which was also supported by our analysis. Healthcare services, specifically the number of ANC^10 16^ visits and place of delivery^17^ also have an impact on neonatal deaths. Nevertheless, in our study, neonatal mortality was higher among those delivering in a facility, outdoors or on the way to a facility than at home. This is likely due to some women in this population starting their delivery at home, and only going to a facility if something goes wrong during labour and delivery, or being referred to the facility because they were high risk. As the proportion of routine deliveries at facilities increases, this association may reverse.

A previous systematic review indicated that neonatal mortality is reduced by vaccination of women of childbearing age with tetanus toxoid in LMICs,^15^ while our study found no association with mortality both before and after adjustment. Neonatal tetanus is an unlikely cause of neonatal deaths due to the high rate of tetanus toxoid vaccination among women of childbearing age in Nepal. A recent programmatic focus on vaccinating adolescent girls with tetanus toxoid may also explain this lack of association. Tetanus toxoid vaccine receipt during pregnancy (as collected in this study) is often used as a proxy for ANC utilisation in LMICs, but we included the number of ANC visits in the regressions and found it to be associated with neonatal mortality. ANC visits are likely a more proximate risk factor for mortality than tetanus toxoid receipt.

Strengths of this study included a large cohort of pregnancies followed through 28 days postpartum with up to eight visits in the neonatal period. The frequent visits reduced loss to follow-up and improved estimates of age at death. While gestational age was not measured using gold standard ultrasound, the date of last menstrual period was obtained early in the pregnancy, limiting recall bias. However, the reliance on date of last menstrual period could have led to some misclassification of preterm births. Another strength of this study was that stillbirths were identified using maternal recall of whether the baby moved, cried or breathed after birth. If the infant did none of these, they were classified as a stillbirth. However, there could be some misclassification of stillbirths as live births and vice versa. Another limitation is that we did not include maternal morbidity in pregnancy in this analysis. Maternal morbidity may have impacted neonatal mortality but would likely have worked through adverse pregnancy outcomes such as preterm birth and SGA, which were the primary focus of risk factors for mortality in this analysis. Some other limitations include many missing weights due to early newborn death or weighing of the baby 72 hours or more after birth. Most of the missing values for birth weight were estimated by using multiple imputation, which increased the uncertainty of this analysis. The imputation of birth weights also had a qualitative impact on the regression coefficients, in particular for the AGA/LGA and preterm category, which went from an aHR of 3.13 to one of 5.46. This may be due to the fact that preterm infants who died soon after birth were likely to be missing weights. The neonatal mortality rate among preterm births was 33.02 per 1000 before imputation and 83.19 per 1000 after imputation. Similarly, the neonatal mortality rate among SGA before imputation was 19.10 per 1000 and 32.92 per 1000 after imputation.

In addition to missing birth weights, about 10% of values were missing for place of delivery and number of ANC visits. We also conducted sensitivity analyses including and excluding these covariates and found that the other regression coefficients were not altered significantly by their being included.

Our study found that maternal age, wealth quintile, caste of the family, parity and tetanus vaccination were not significantly associated with neonatal mortality after adjustment and imputation. Although these findings conflict with some of the existing literature, it is possible that the inclusion of more proximate risk factors such as gestational age and size for gestational age lessened the importance of these other more distal risk factors.

## Conclusion

Maternal height, maternal education, prior child deaths, SGA, preterm, twins or triplets, place of delivery and number of ANC visits were significantly associated with neonatal mortality risk. Low birthweight babies who are preterm and SGA, preterm and LGA, or SGA and term have different mortality risks and these adverse outcomes likely have different aetiologies. These findings could help refine interventions to reduce these adverse birth outcomes. Interventions that focus on preventing SGA and preterm births, improving education for women, promoting quality antenatal healthcare and facility delivery, and care of vulnerable infants after birth should continue to be emphasised and prioritised.

## Supplementary Material

Reviewer comments

Author's manuscript

## Data Availability

Data are available upon reasonable request.

## References

[1] United Nations International Children’s Emergency Fund, Unicef. Neonatal mortality. 2023. Available: https://data.unicef.org/topic/child-survival/neonatal-mortality/#:~:text=Globally%2C%202.3%20million%20children%20died,6%2C400%20neonatal%20deaths%20every%20day

[2] Ministry of Health and Population, Nepal; New ERA; and ICF. Nepal Demographic and Health Survey 2022: Key Indicators Report. Kathmandu, Nepal: Ministry of Health and Population, 2022.

[3] Paudel D, Thapa A, Shedain PR, et al. Trends and determinants of neonatal mortality in Nepal: further analysis of the Nepal demographic and health surveys, 2001-2011; 2013. DHS further analysis report

[4] Kc A, Jha AK, Shrestha MP, et al. Trends for neonatal deaths in Nepal (2001–2016) to project progress towards the SDG target in 2030, and risk factor analyses to focus action. Matern Child Health J 2020;24(Suppl 1):5–14. 10.1007/s10995-019-02826-0 31773465 PMC7048722

[5] Government of Nepal, National Planning Commission. National Planning Commission, 2015: Sustainable Development Goals, 2016-2030, National (Preliminary) Report. Kathmandu, Nepal, 2015: 39–52.

[6] Public Health Update. Number of public health facilities in Nepal. 2020. Available: https://publichealthupdate.com/number-of-health-facilities-in-nepal/

[7] Joshi R, Sharma S, Teijlingen E. Improving neonatal health in Nepal: major challenges to achieving millennium development goal 4. Health Science Journal 2013;7:247–57.

[8] Mishra SR, Khanal P, Karki DK, et al. National health insurance policy in Nepal: challenges for implementation. Glob Health Action 2015;8:28763. 10.3402/gha.v8.28763 26300556 PMC4546934

[9] Kamal S, Ashrafuzzaman M, Nasreen S. Risk factors of neonatal mortality in Bangladesh. J Nepal Paedtr Soc 2012;32:37–46. 10.3126/jnps.v32i1.4845

[10] Batieha AM, Khader YS, Berdzuli N, et al. Level, causes and risk factors of neonatal mortality, in Jordan: results of a national prospective study. Matern Child Health J 2016;20:1061–71. 10.1007/s10995-015-1892-x 26645614

[11] Sharma V, Katz J, Mullany LC, et al. Young maternal age and the risk of neonatal mortality in rural Nepal. Arch Pediatr Adolesc Med 2008;162:828–35. 10.1001/archpedi.162.9.828 18762599 PMC2535853

[12] Kamal SMM. Maternal education as a determinant of neonatal mortality in Bangladesh. Journal of Health Management 2012;14:269–81. 10.1177/0972063412457509

[13] McKinnon B, Harper S, Kaufman JS, et al. Socioeconomic inequality in neonatal mortality in countries of low and middle income: a Multicountry analysis. Lancet Glob Health 2014;2:e165–73. 10.1016/S2214-109X(14)70008-7 25102849

[14] Katz J, West KP, Khatry SK, et al. Risk factors for early infant mortality in Sarlahi district. Bull World Health Organ 2003;81:717–25. 14758431 PMC2572324

[15] Khan AA, Zahidie A, Rabbani F. Interventions to reduce neonatal mortality from neonatal tetanus in Low- and middle-income countries - A systematic review. BMC Public Health 2013;13:322. 10.1186/1471-2458-13-322 23570611 PMC3637612

[16] Arunda M, Emmelin A, Asamoah BO. Effectiveness of Antenatal care services in reducing neonatal mortality in Kenya: analysis of national survey data. Glob Health Action 2017;10:1328796. 10.1080/16549716.2017.1328796 28621201 PMC5496054

[17] Ajaari J. Impact of place of delivery on neonatal mortality in rural Tanzania. Value in Health 2013;16:A209–10. 10.1016/j.jval.2013.03.1059 PMC4948160 27621958

[18] Subedi S, Katz J, Erchick DJ, et al. Does higher early neonatal mortality in boys reverse over the neonatal period? A pooled analysis from three trials of Nepal. BMJ Open 2022;12:e056112. 10.1136/bmjopen-2021-056112 PMC9121405 35589346

[19] Saya AR, Katz J, Khatry SK, et al. Causes of neonatal mortality using verbal Autopsies in rural Southern Nepal, 2010–2017. PLOS Glob Public Health 2022;2:e0001072. 10.1371/journal.pgph.0001072 36962665 PMC10021801

[20] World Health Organization. Indicator Metadata Registry list/neonatal mortality rate (0 to 27 days) per 1000 live births). 2022. Available: https://www.who.int/data/gho/indicator-metadata-registry/imr-details/67

[21] World Health Organization. Low birth weight. nutrition and nutrition-related health and development data. 2022. Available: https://www.who.int/data/nutrition/nlis/info/low-birth-weight

[22] Gill SV, May-Benson TA, Teasdale A, et al. Birth and developmental correlates of birth weight in a sample of children with potential sensory processing disorder. BMC Pediatr 2013;13:29. 10.1186/1471-2431-13-29 23442948 PMC3598529

[23] Gestational age assessment: II. prediction from combined clinical observations. Am J Obstet Gynecol 1981;140:770–4. 10.1016/0002-9378(81)90738-9 7258258

[24] Swann T. “'anarchist Technologies': anarchism, cybernetics and mutual aid in community responses to the COVID-19 crisis”. Organization (Lond) 2023;30:193–209. 10.1177/13505084221090632 37038431 PMC10076238

[25] Spong CY. “Defining “term” pregnancy: recommendations from the defining “term” pregnancy Workgroup”. JAMA 2013;309:2445–6. 10.1001/jama.2013.6235 23645117

[26] Galal M, Symonds I, Murray H, et al. Postterm pregnancy. Facts, Views & Vision in ObGyn 2012;4:175. PMC3991404 24753906

[27] Villar J, Ismail LC, Victora CG, et al. International standards for newborn weight, length, and head circumference by gestational age and sex: the newborn cross-sectional study of the INTERGROWTH-21St project. The Lancet 2014;384:857–68. 10.1016/S0140-6736(14)60932-6 25209487

[28] Tidy C, Payne J. Gravidity and Parity Definitions (Implications in Risk Assessment). 2019.

[29] StataCorp. Stata Statistical Software: Release 16. College Station, TX, 2019.

[30] Siegel EH, Stoltzfus RJ, Khatry SK, et al. Epidemiology of anemia among 4- to 17-month-old children living in South central Nepal. Eur J Clin Nutr 2006;60:228–35. 10.1038/sj.ejcn.1602306 16234835 PMC1360164

[31] Hazel EA, Mullany LC, Zeger SL, et al. Development of an imputation model to Recalibrate Birthweights measured in the early neonatal period to time at delivery and assessment of its impact on size-for-gestational age and low birthweight prevalence estimates: a secondary analysis of a pregnancy cohort in rural Nepal. BMJ Open 2022;12:e060105. 10.1136/bmjopen-2021-060105 PMC9277385 35820766

[32] Lin DY, Wei L-J. The robust inference for the Cox proportional hazards model. Journal of the American Statistical Association 1989;84:1074–8. 10.1080/01621459.1989.10478874

[33] Boerma T, Requejo J, Victora CG, et al. Countdown to 2030: tracking progress towards universal coverage for reproductive, maternal, newborn, and child health. The Lancet 2018;391:1538–48. 10.1016/S0140-6736(18)30104-1 29395268

[34] Victora CG, Requejo JH, Barros AJD, et al. Countdown to 2015: A decade of tracking progress for maternal, newborn, and child survival. The Lancet 2016;387:2049–59. 10.1016/S0140-6736(15)00519-X PMC7613171 26477328

[35] Neupane S, Doku DT. Neonatal mortality in Nepal: a Multilevel analysis of a nationally representative sample. [corrected]. J Epidemiol Glob Health 2014;4:213–22. 10.1016/j.jegh.2014.02.001 25107657 PMC7333823

[36] De Jesus LC, Pappas A, Shankaran S, et al. Risk factors for post-neonatal intensive care unit discharge mortality among extremely low birth weight infants. J Pediatr 2012;161:70–4. 10.1016/j.jpeds.2011.12.038 22325187 PMC3366175

[37] Khatun W, Alam A, Rasheed S, et al. Exploring the Intergenerational effects of Undernutrition: Association of maternal height with neonatal, infant and under-five mortality in Bangladesh. BMJ Glob Health 2018;3:e000881. 10.1136/bmjgh-2018-000881 PMC6254740 30498585

[38] Pulver LS, Guest-Warnick G, Stoddard GJ, et al. Weight for gestational age affects the mortality of late Preterm infants. Pediatrics 2009;123:e1072–7. 10.1542/peds.2008-3288 19482740

[39] Yasmin S, Osrin D, Paul E, et al. Neonatal mortality of low-birth-weight infants in Bangladesh. Bull World Health Organ 2001;79:608–14. 11477963 PMC2566474

[40] Blickstein I, Keith LG. Neonatal mortality rates among growth-discordant twins, classified according to the birth weight of the smaller twin. Am J Obstet Gynecol 2004;190:170–4. 10.1016/j.ajog.2003.07.025 14749655

[41] Clausson B, Cnattingius S, Axelsson O. Preterm and term births of small for gestational age infants: A population-based study of risk factors among nulliparous women. BJOG:An International Journal of O&G 1998;105:1011–7. 10.1111/j.1471-0528.1998.tb10266.x 9763054

[42] Kozuki N, Lee AC, Silveira MF, et al. The associations of parity and maternal age with small-for-gestational-age, preterm, and neonatal and infant mortality: a meta-analysis. BMC Public Health 2013;13. 10.1186/1471-2458-13-S3-S2 PMC3847520 24564800

[43] Garite TJ, Clark RH, Elliott JP, et al. Twins and triplets: the effect of plurality and growth on neonatal outcome compared with Singleton infants. Am J Obstet Gynecol 2004;191:700–7. 10.1016/j.ajog.2004.03.040 15467528

[44] Wulandari RD, Laksono AD. Is parity a Predictor of neonatal death in Indonesia analysis of the 2017 Indonesia demographic and health survey. Indian Journal of Forensic Medicine and Toxicology 2020;14.

