# Supplementary material for: Risk factors for neonatal mortality: an observational cohort study in Sarlahi district of rural southern Nepal
Source: BMJ Open. 2023 Sep 14;13(9):e066931. doi: 10.1136/bmjopen-2022-066931 (PMC10503364; doi:10.1136/bmjopen-2022-066931)
Supplement: Supplementary data [file bmjopen-2022-066931supp001.pdf]

APPENDIX: SUPPLEMENTAL MATERIALS

Table S1 Multivariate Cox Regression Model S1

Variables	Crude Hazard Ratio	95% CI	**Model S1:** Maternal Age, Mother's Height, Mother's Years of Education, Wealth Quintile, Caste, Parity, Prior Child Deaths, Tetanus Vaccination, Place of Delivery, Number of ANC Visits, Sex of Child, Gestational Age, Size for Gestation, Singleton/Twin or Triplet			
			**Adjusted Hazard Ratio (N=26,626)**	**95% CI**	**Adjusted HR (Using imputed SGA) (N=27,158)**	**95% CI**
**Maternal Age (years) at LMP**						
Mean, 95% CI: 22.46 (22.41, 22.51)						
<=18	1.57	(1.34, 1.83)	1.34	(1.00, 1.76)	1.25	(1.02, 1.52)
18-35	1.00	Reference	1.00	Reference	1.00	Reference
>35	1.62	(1.14, 2.31)	1.39	(0.64, 3.04)	1.25	(0.74, 2.10)
**Maternal Height (cm)**						
Mean, 95% CI: 150.55 (150.49, 150.61)						
<145	1.00	Reference	1.00	Reference	1.00	Reference
145-150	0.70	(0.59, 0.83)	0.69	(0.52, 0.93)	0.82	(0.67, 1.00)
>=150	0.49	(0.41, 0.57)	0.63	(0.48, 0.84)	0.59	(0.49, 0.72)
**Maternal Education (years)**						
Mean, 95% CI: 2.61 (2.56, 2.65)						
0	1.00	Reference	1.00	Reference	1.00	Reference
1-5	0.81	(0.64, 1.03)	0.98	(0.67, 1.43)	0.95	(0.74, 1.24)
>5	0.64	(0.54, 0.76)	0.75	(0.53, 1.04)	0.77	(0.62, 0.97)
**Wealth Quintile**						
Poorest	1.00	Reference	1.00	Reference	1.00	Reference
Poorer	0.87	(0.72, 1.05)	0.92	(0.66, 1.27)	0.95	(0.76, 1.17)
Middle	0.80	(0.66, 0.97)	1.03	(0.74, 1.43)	1.00	(0.80, 1.25)
Richer	0.60	(0.49, 0.74)	0.82	(0.56, 1.19)	0.88	(0.69, 1.12)
Richest	0.59	(0.48, 0.73)	1.01	(0.68, 1.50)	1.00	(0.77, 1.31)
**Caste of the Family**						
Brahmin and Chhetri	1.00	Reference	1.00	Reference	1.00	Reference
Vaishya	1.10	(0.74, 1.63)	1.19	(0.53, 2.67)	0.86	(0.54, 1.37)
Shudra	1.57	(1.05, 2.37)	1.48	(0.63, 3.46)	1.00	(0.61, 1.64)
Muslim and others	1.01	(0.65, 1.57)	1.06	(0.44, 2.57)	0.73	(0.44, 1.23)
**Parity**						
1-4	1.00	Reference	1.00	Reference	1.00	Reference
>=5	1.75	(1.33, 2.30)	1.18	(0.65, 2.13)	1.02	(0.68, 1.53)
Prior pregnancy but no parity	1.75	(1.24, 2.47)	2.04	(0.45, 9.35)	1.50	(0.65, 3.46)
No prior pregnancy	1.58	(1.38, 1.82)	1.38	(1.02, 1.85)	1.68	(1.29, 1.93)
**Prior child death**						
No prior pregnancy	1.76	(1.52, 2.04)	Omitted		Omitted	
Prior live births but no deaths	1.00	Reference	1.00	Reference	1.49	(0.67, 3.29)
Prior live births and child death	1.80	(1.48, 2.18)	1.14	(0.82, 1.58)	1.00	Reference
Prior pregnancy but no live birth	1.83	(1.33, 2.53)	0.75	(0.18, 3.09)	1.55	(1.26, 1.91)
**Tetanus Vaccination in past 2 years**						
No	1.00	Reference	1.00	Reference	1.00	Reference
Yes	0.78	(0.66, 0.92)	0.84	(0.64, 1.11)	0.81	(0.67, 0.96)
**Place of Delivery**						
At home or Maiti*	1.00	Reference	1.00	Reference	1.00	Reference
At health post/clinic or in hospital	1.22	(1.06, 1.40)	0.92	(0.71, 1.18)	1.30	(1.10, 1.54)
On the way to facility or outdoors	2.43	(1.70, 3.48)	1.20	(0.66, 2.17)	2.08	(1.47, 2.95)
**Number of Antenatal Care (ANC)**						
0	1.00	Reference	1.00	Reference	1.00	Reference
1	1.07	(0.86, 1.34)	0.99	(0.70, 1.39)	1.05	(0.84, 1.31)
2 or 3	1.12	(0.93, 1.35)	1.04	(0.78, 1.39)	1.10	(0.91, 1.34)
>=4	0.61	(0.50, 0.76)	0.77	(0.54, 1.10)	0.70	(0.55, 0.90)
**Sex of Child**						
Male	1.00	Reference	1.00	Reference	1.00	Reference
Female	0.88	(0.77, 0.99)	1.16	(0.94, 1.43)	0.91	(0.80, 1.05)
**Gestational Age**						
Very preterm (<32 weeks)	17.44	(14.54, 20.91)	45.45	(27.63, 74.75)	20.7	(13.88, 30.86)
Moderate to late preterm (32-37 weeks)	2.66	(2.25, 3.14)	2.85	(2.11, 3.85)	2.59	(2.07, 3.25)
Term (37-42 weeks)	1.00	Reference	1.00	Reference	1.00	Reference
Post-term (>= 42 weeks)	1.42	(1.16, 1.75)	1.14	(0.82, 1.59)	1.25	(0.98, 1.59)
**Size for Gestational Age (within 72 hours)**						
AGA	1.00	Reference	1.00	Reference		
SGA	1.76	(1.42, 2.18)	2.30	(1.77, 3.00)		
LGA	0.63	(0.35, 1.12)	0.09	(0.04, 0.19)		
**Imputed Size for Gestational Age**						
AGA	1.00	Reference			1.00	Reference
SGA	1.43	(1.10, 1.87)			1.66	(1.10, 2.50)
LGA	4.52	(3.49, 5.85)			0.75	(0.52, 1.10)
**Singleton/Twin/Triplet**						
Singleton	1.00	Reference	1.00	Reference	1.00	Reference
Twin or Triplet	8.31	(6.43, 10.74)	3.26	(2.04, 5.21)	3.77	(2.75, 5.17)

* Maiti is the maternal home, where women may go to deliver, especially in the first pregnancy

BMJ Publishing Group Limited (BMJ) disclaims all liability and responsibility arising from any reliance placed on this supplemental material which has been supplied by the author(s)

Table S2 Multivariate Cox Regression Model S2

Variables	Crude Hazard Ratio	95% CI	**Model S2:** Maternal Age, Mother's Height, Mother's Years of Education, Wealth Quintile, Caste, Parity, Prior Child Death, Tetanus Vaccination, Sex of Child, Gestational Age, Size for Gestation, Singleton/Twin or Triplet			
			Adjusted Hazard Ratio (N=26,680)	95% CI	Adjusted HR (Using imputed SGA) (N=30,332)	95% CI
**Maternal Age (years) at LMP**						
Mean, 95% CI: 22.46 (22.41, 22.51)						
<=18	1.57	(1.34, 1.83)	1.35	(1.02, 1.80)	1.15	(0.96, 1.39)
18-35	1.00	Reference	1.00	Reference	1.00	Reference
>35	1.62	(1.14, 2.31)	1.36	(0.63, 2.93)	1.32	(0.82, 2.15)
**Maternal Height (cm)**						
Mean, 95% CI: 150.55 (150.49, 150.61)						
<145	1.00	Reference	1.00	Reference	1.00	Reference
145-150	0.70	(0.59, 0.83)	0.69	(0.51, 0.93)	0.83	(0.69, 1.01)
>=150	0.49	(0.41, 0.57)	0.63	(0.48, 0.84)	0.60	(0.50, 0.72)
**Maternal education (years)**						
Mean, 95% CI: 2.61 (2.56, 2.65)						
No schooling	1.00	Reference	1.00	Reference	1.00	Reference
1-5	0.81	(0.64, 1.03)	0.95	(0.65, 1.39)	0.92	(0.72, 1.17)
>5	0.64	(0.54, 0.76)	0.71	(0.51, 0.99)	0.75	(0.61, 0.93)
**Wealth Quintile**						
Poorest	1.00	Reference	1.00	Reference	1.00	Reference
Poorer	0.87	(0.72, 1.05)	0.91	(0.66, 1.25)	0.99	(0.81, 1.22)
Middle	0.80	(0.66, 0.97)	1.00	(0.72, 1.39)	0.99	(0.80, 1.22)
Richer	0.60	(0.49, 0.74)	0.79	(0.54, 1.15)	0.88	(0.70, 1.10)
Richest	0.59	(0.48, 0.73)	0.99	(0.67, 1.46)	0.99	(0.78, 1.27)
**Caste of the Family**						
Brahmin and Chhetri	1.00	Reference	1.00	Reference	1.00	Reference
Vaishya	1.10	(0.74, 1.63)	1.23	(0.54, 2.77)	0.88	(0.57, 1.34)
Shudra	1.57	(1.05, 2.37)	1.54	(0.66, 3.60)	0.99	(0.62, 1.56)
Muslim	1.01	(0.65, 1.57)	1.10	(0.45, 2.65)	0.70	(0.43, 1.13)
**Parity**						
1-4	1.00	Reference	1.00	Reference	1.00	Reference
>=5	1.75	(1.33, 2.30)	1.19	(0.66, 2.15)	1.03	(0.70, 1.51)
Prior pregnancy but no parity	1.75	(1.24, 2.47)	2.08	(0.45, 9.57)	1.38	(0.65, 2.93)
No prior pregnancy	1.58	(1.38, 1.82)	1.34	(1.01, 1.77)	1.65	(1.38, 1.98)
**Prior child deaths**						
No prior pregnancy	1.76	(1.52, 2.04)	Omitted		Omitted	
Prior live births but no deaths	1.00	Reference	1.00	Reference	1.00	Reference
Prior live births and child death	1.80	(1.48, 2.18)	1.14	(0.82, 1.59)	1.49	(1.22, 1.82)
Prior pregnancy but no live birth	1.83	(1.33, 2.53)	0.69	(0.17, 2.87)	1.54	(0.76, 3.11)
**Tetanus Vaccination in past 2 years**						
No	1.00	Reference	1.00	Reference	1.00	Reference
Yes	0.78	(0.66, 0.92)	0.84	(0.64, 1.10)	0.94	(0.79, 1.11)
**Sex of Child**						
Male	1.00	Reference	1.00	Reference	1.00	Reference
Female	0.88	(0.77, 0.99)	1.18	(0.96, 1.46)	0.89	(0.78, 1.01)
**Gestational Age**						
Very preterm (<32 weeks)	17.44	(14.54, 20.91)	47.53	(28.85, 78.31)	22.57	(15.95, 31.95)
Moderate to late preterm (32-37 weeks)	2.66	(2.25, 3.14)	2.92	(2.16, 3.94)	2.70	(2.21, 3.30)
Term (37-42 weeks)	1.00	Reference	1.00	Reference	1.00	Reference
Post-term (42-45 weeks)	1.42	(1.16, 1.75)	1.13	(0.81, 1.58)	1.25	(1.00, 1.57)
**Size for Gestational Age (within 72 hours)**						
AGA	1.00	Reference	1.00	Reference		
SGA	1.76	(1.42, 2.18)	2.31	(1.78, 3.00)		
LGA	0.63	(0.35, 1.12)	0.09	(0.04, 0.19)		
**Imputed Size for Gestational Age**						
AGA	1.00	Reference			1.00	Reference
SGA	1.43	(1.10, 1.87)			1.66	(1.18, 2.34)
LGA	4.52	(3.49, 5.85)			0.76	(0.52, 1.10)
**Singleton/Twin/Triplet**						
Singleton	1.00	Reference	1.00	Reference	1.00	Reference
Twin or Triplet	8.31	(6.43, 10.74)	3.15	(1.98, 5.04)	3.97	(2.97, 5.30)
