# Supplementary material for: Risk factors for neonatal mortality: an observational cohort study in Sarlahi district of rural southern Nepal
Source: BMJ Open. 2023 Sep 14;13(9):e066931. doi: 10.1136/bmjopen-2022-066931 (PMC10503364; doi:10.1136/bmjopen-2022-066931)
Supplement: Author's manuscript [file bmjopen-2022-066931.draft_revisions.pdf]

BMJ Open is committed to open peer review. As part of this commitment we make the peer review history of every article we publish publicly available.

When an article is published we post the peer reviewers' comments and the authors' responses online. We also post the versions of the paper that were used during peer review. These are the versions that the peer review comments apply to.

The versions of the paper that follow are the versions that were submitted during the peer review process. They are not the versions of record or the final published versions. They should not be cited or distributed as the published version of this manuscript.

BMJ Open is an open access journal and the full, final, typeset and author-corrected version of record of the manuscript is available on our site with no access controls, subscription charges or pay-per-view fees (http://bmjopen.bmj.com).

If you have any questions on BMJ Open's open peer review process please email info.bmjopen@bmj.com

BMJ Open

Risk Factors for Neonatal Mortality in Sarlahi District of Rural Southern Nepal

Journal:	*BMJ Open*
Manuscript ID	bmjopen-2022-066931
Article Type:	Original research
Date Submitted by the Author:	20-Aug-2022

Complete List of Authors:	Yan, Tingting; Johns Hopkins University Bloomberg School of Public Health, International Health<br>Mullany, Luke C.; Johns Hopkins University Bloomberg School of Public Health, International Health<br>Subedi, Seema; Johns Hopkins University Bloomberg School of Public Health, International Health<br>Hazel , Elizabeth; Johns Hopkins University Bloomberg School of Public Health<br>Khatry, Subarna; Johns Hopkins University Bloomberg School of Public Health, Department of International Health; Nepal Eye Hospital Complex, Nepal Nutrition Intervention Project - Sarlahi (NNIPS)<br>Mohan, Diwakar; Johns Hopkins University Bloomberg School of Public Health, Department of International Health<br>Zeger, Scott; Johns Hopkins University Bloomberg School of Public Health, Department of Biostatistics<br>Tielsch, James; George Washington University School of Public Health and Health Services, Department of Global Health<br>LeClerq, Steven; Johns Hopkins University Bloomberg School of Public Health, Department of International Health; Nepal Eye Hospital Complex, Nepal Nutrition Intervention Project - Sarlahi (NNIPS)<br>Katz, Joanne; Johns Hopkins University Bloomberg School of Public Health, Department of International Health

Keywords:	Epidemiology < TROPICAL MEDICINE, Public health < INFECTIOUS DISEASES, Risk management < HEALTH SERVICES ADMINISTRATION & MANAGEMENT

SCHOLARONE™ Manuscripts

I, the Submitting Author has the right to grant and does grant on behalf of all authors of the Work (as defined in the below author licence), an exclusive licence and/or a non-exclusive licence for contributions from authors who are: i) UK Crown employees; ii) where BMJ has agreed a CC-BY licence shall apply, and/or iii) in accordance with the terms applicable for US Federal Government officers or employees acting as part of their official duties; on a worldwide, perpetual, irrevocable, royalty-free basis to BMJ Publishing Group Ltd ("BMJ") its licensees and where the relevant Journal is co-owned by BMJ to the co-owners of the Journal, to publish the Work in this journal and any other BMJ products and to exploit all rights, as set out in our licence.

The Submitting Author accepts and understands that any supply made under these terms is made by BMJ to the Submitting Author unless you are acting as an employee on behalf of your employer or a postgraduate student of an affiliated institution which is paying any applicable article publishing charge ("APC") for Open Access articles. Where the Submitting Author wishes to make the Work available on an Open Access basis (and intends to pay the relevant APC), the terms of reuse of such Open Access shall be governed by a Creative Commons licence – details of these licences and which Creative Commons licence will apply to this Work are set out in our licence referred to above.

Other than as permitted in any relevant BMJ Author's Self Archiving Policies, I confirm this Work has not been accepted for publication elsewhere, is not being considered for publication elsewhere and does not duplicate material already published. I confirm all authors consent to publication of this Work and authorise the granting of this licence.

For peer review only

Risk Factors for Neonatal Mortality in Sarlahi District of Rural Southern Nepal

Tingting Yan, MSPH[1], Luke C Mullany, PhD[1], Seema Subedi, MPH[1], Elizabeth Hazel, PhD[1], Subarna K Khatry, MBBS, FRCS[1,2], Diwakar Mohan, MD, DrPH[1], Scott Zeger, PhD[3], James M Tielsch, PhD[4], Steven C LeClerq, MPH[1,2], Joanne Katz, ScD[1#]

1. Department of International Health, Johns Hopkins Bloomberg School of Public Health, 615 N. Wolfe Street, Baltimore, MD, 21205, USA
2. Nepal Nutrition Intervention Project - Sarlahi (NNIPS), Nepal Eye Hospital Complex, PO Box 335, Tripureshwor, Kathmandu, Nepal
3. Department of Biostatistics, Johns Hopkins Bloomberg School of Public Health, 615 N. Wolfe Street, Baltimore, MD, 21205, USA
4. Department of Global Health, Milken Institute School of Public Health, George Washington University, 950 New Hampshire Avenue, Washington, DC, USA

Corresponding author: Joanne Katz, ScD. Email: jkatz1@jhu.edu

Word Count. 3222 words. Abstract 291.

Number of figures and tables. This manuscript included 2 figures and 3 tables (2 in supplemental materials).

For peer review only

ABSTRACT

Objectives To assess the association between maternal characteristics, adverse birth outcomes (small-for-gestational-age (SGA) and/or preterm) and neonatal mortality in rural Nepal.

Design This is a secondary observational analysis of data from a randomized trial to assess the impact of newborn massage with different oils on neonatal mortality in Sarlahi district, Nepal.

Setting Rural Sarlahi district, Nepal

Participants 40,119 pregnant women enrolled from September 9, 2010, to Jan 16, 2017

Main outcome The outcome variable is neonatal death. Cox regression was used to estimate adjusted Hazard Ratios (aHR) to assess the association between adverse birth outcomes and neonatal mortality.

Results There were 32,004 live births and 998 neonatal deaths. SGA and/or preterm birth was strongly associated with increased neonatal mortality: SGA and preterm [aHR: 7.09, 95% CI: (4.44, 11.31)], SGA and term/post-term [aHR: 2.12, 95% CI: (1.58, 2.86)], appropriate-for-gestational-age (AGA)/large-for-gestational-age (LGA) and preterm [aHR: 3.23, 95% CI: (2.30, 4.54)]. Neonatal mortality risk was increased with a history of prior child deaths [aHR: 1.53, 95% CI: (1.24, 1.87)], twins or triplets [aHR: 5.64, 95% CI: (4.25, 7.48)], births at health posts/clinics or in hospital [aHR: 1.34, 95% CI: (1.13, 1.58)] and on the way to facilities or outdoors [aHR: 2.26, 95% CI: (1.57, 3.26)]. Risk was lower with increasing maternal height from <145 cm to 145-150 cm [aHR: 0.78, 95% CI: (0.65, 0.94)] to ≥150 cm [aHR: 0.57, 95% CI: (0.47, 0.68)], four or more antenatal care (ANC) visits [aHR: 0.67, (0.53, 0.86)] and education >5 years [aHR: 0.75, 95% CI: (0.62, 0.92)].

Conclusion SGA and/or preterm birth is strongly associated with increased neonatal mortality. To reduce neonatal mortality, interventions that prevent SGA and preterm births by promoting antenatal care and facility delivery, and care of high-risk infants after birth should be tested.

Trial Registration Number

NCT01177111

Strengths and limitations of the study

- A large size of pregnancies followed with frequent visits in the neonatal period ensures a large, statistically powered study to be performed.
- The availability of a large amount of data for each participants enables a wide range of risk factor to be investigated.

For peer review only

- The date of last menstrual period was obtained, limiting recall bias, and the stillbirths were identified, but both could lead to some misclassification.
- Many missing weights due to early newborn death or weighing of the baby 72 hours or more after birth were estimated by using multiple imputation, which increased the uncertainty of this analysis.

For peer review only

INTRODUCTION

Each year approximately 2.9 million neonates die worldwide(1). Almost 99% of these deaths occur in low- and middle-income countries(2). Neonatal mortality in Nepal continues to decline, from 38 per 1,000 live births in 2000 to 21 in 2016(2), but accounts for a higher percentage of under-5 child deaths because mortality rates among older children have decreased faster than neonatal mortality(3). If the trend continues, it will take much longer to reach the Sustainable Development Goals target of reducing neonatal mortality to 12 per 1,000 live births by 2030(4).

Studies have shown numerous risk factors contributing to neonatal mortality(5–11).
Small-for-gestational-age (SGA) and preterm births are associated with neonatal deaths in LMICs(6). Other factors include maternal age(7) and education(8), household income(9), previous pregnancy history(10), tetanus vaccination(11), attendance at antenatal care (ANC)(12), and place of delivery(13).

We used data from the Nepal Oil Massage Study (NOMS), a community-based, cluster-randomized controlled trial in rural southern Nepal to assess the association between maternal characteristics, adverse birth outcomes (small-for-gestational-age (SGA) and/or preterm) and other maternal and infant characteristics and neonatal mortality to provide evidence for intervention on reducing neonatal mortalit.

METHODS

Study Setting

This study was conducted in Sarlahi District of Southern Nepal, a rural area of subsistence farming in the low-lying plains of Nepal bordering Bihar, India.

Study Design

The NOMS study was a cluster-randomized, community-based trial (Clinicaltrials.gov (NCT01177111)). The geographic area was divided into sectors that were randomized to receive promotion of full-body massage with mustard oil, which is the standard practice, or the intervention group that received promotion of full-body massage with sunflower seed oil. All women of childbearing age in the 34 participating Village Development Committees (VDCs) were eligible for the trial. Women who consented to pregnancy surveillance were visited every 5 weeks and asked whether they had their period since the last visit. If not, they were offered a pregnancy test. If positive, they were enrolled in the trial. Women were followed with monthly visits until delivery. In late pregnancy (30-32 weeks), women were provided with either sunflower seed or mustard seed oil, based on their allocation to randomized sectors. A birth assessment was conducted on the first visit to the newborn baby (the day of birth or as soon after birth as possible), and neonates were followed up on 1, 3, 7, 10, 14, 21 and 28 days of age. Vital status was collected on mothers and infants at

For peer review only

each of these time points. A verbal autopsy was conducted on the causes of neonatal death and the date of death was also recorded. For the analysis of risk factors for neonatal mortality, only live born infants and their mothers were included.

Data Collection

Data for the trial were collected from November 2010 through January 2017. At the time women were identified as pregnant and enrolled, date of last menstrual period, maternal age, height, education and reproductive history were recorded. Information about household assets, caste and ethnicity of the family was also collected. The enrollment form and monthly visits in pregnancy recorded receipt of tetanus vaccination, tobacco and alcohol use, and maternal morbidities. In the first visit after birth, late pregnancy morbidity and labor and delivery characteristics were collected. Neonatal characteristics included sex, weight and the time since birth at which weight was measured. The aim was to measure weight as soon after birth as possible.

Patient and Public Involvement

Our study was an IRB-approved secondary analysis of a pre-existing dataset generated in NOMS study. Therefore, it was not appropriate to involve patients or the public in the design, or conduct, or reporting, or dissemination plans of our research.

Definitions of Variables

Neonatal death was defined as the death of a live born baby in the first 28 days of life and the neonatal mortality rate was defined as the number of neonatal deaths per 1000 live births. Low birth weight was defined as a birth weight less than 2500 grams (g). Birth weight was also divided into 4 groups: <1500g as very low birth weight (VLBW), 1500-2500g as moderately low birth weight (LBW), 2500-4000g (4000g included) as normal birth weight (NBW), >4000g as high birth weight (HBW). Gestational age was calculated by taking the number of days between pregnancy outcome and date of last menstrual period obtained at enrollment in the trial. Preterm births were babies born alive before 37 completed weeks gestation. Gestational age was also categorized into 4 groups: <32, 32-<37, 37-<42, 42-45 weeks, and labeled as very preterm, moderate to late preterm, term, and post-term, respectively.

Small-for-gestational-age (SGA) was defined as newborns with weight below the 10th percentile of newborns using the Intergrowth reference population(14). Appropriate-for-gestational-age (AGA) was defined as weight between the 10th and 90th percentiles of the reference population, and large-for-gestational-age (LGA) was defined as a weight above the 90th percentile of the reference population. Parity was defined as the number of prior pregnancies resulting in a live or stillbirth. Gravidity

For peer review only - http://bmjopen.bmj.com/site/about/guidelines.xhtml

was the number of prior pregnancies, regardless of the outcome of the pregnancy.

Data Analysis

The data described above were managed and analyzed using Stata version 16.0(15). The association between potential risk factors (maternal and neonatal characteristics) and neonatal death was examined using crude hazard ratios (cHR) and 95% confidence intervals (CI) obtained from simple Cox regression models. Cox regression with multiple predictors was used to estimate the adjusted hazard ratios (aHR) and 95% CIs. Cumulative mortality curves (1-The inverse of Kaplan-Meier (K-M) survival curves) (cumulative mortality) were used to describe the differences in neonatal mortality by SGA and preterm status for, specifically four groups: (SGA and preterm, SGA and term/post-term, AGA/LGA and preterm, and AGA/LGA and term/post-term).

Maternal characteristics included demographics, socioeconomic status, lifestyle and reproductive history. Maternal age in pregnancy was categorized as ≤ 18, 18 to 35, and >35 years. Maternal height was categorized as <145, 145 to <150, and ≥ 150 cm. Socioeconomic information included years of education (no schooling as reference, 1 to ≤ 5, and > 5 years), and wealth quintiles based on a standardized score of the total number of household assets(16), caste of the family (Brahmin and Chhetri, Vaishya, Shudra, and Muslim). Reproductive information included parity (prior pregnancy but no parity, no prior pregnancy, 1-4, and ≥5), gravidity (0, and ≥ 1), last pregnancy outcome (no prior pregnancy, at least one live birth, stillbirth, or miscarriage and abortion), a history of child deaths, any prior pregnancy that ended in stillbirth or miscarriage, and if the current pregnancy was a multiple birth (twins or triplets). Maternal exposures in pregnancy included self-reported tobacco and/or alcohol use.

For health care in pregnancy and characteristics of delivery, tetanus vaccination receipt, place of delivery (at home or maternal home, at a health post/clinic or in hospital, or on the way to the facility or outdoors), and the number of antenatal care (ANC) visits (0, 1, 2 or 3, ≥ 4) were examined.

Neonatal characteristics included birth weight (taken within 72 hours of birth), gestational age, sex, size for gestational age and singleton/twin/triplet in our analysis. Because weights were taken at varying times after birth, and birth weight was missing for some infants who died very soon after birth, we multiply imputed birth weights (and hence SGA percentiles) for those with missing weights and also imputed weight at time zero for all those with weights and times of weight measurement. This was done using a regression model based on longitudinal daily weights from day of birth through 10 days in a population of infants from the same study area as this trial(17).

In the simple Cox regression models, we calculated the cHR and its 95% CI for each variable. In

For peer review only - http://bmjopen.bmj.com/site/about/guidelines.xhtml

multivariable Cox regression, we developed 4 different models to examine the aHR and its 95% CI for risk factors. In model 1, we included maternal age, height, years of education, wealth quintile, caste, parity, prior child deaths, tetanus vaccination, place of delivery, number of ANC visits, sex of the infant, preterm, adverse birth outcomes (SGA and preterm, SGA and term/post-term, AGA/LGA and preterm, and AGA/LGA and term/post-term), and singleton/twin/triplet. In model 2, we included all the variables in model 1, except place of delivery and number of ANC visits. We ran the models with and without these two variables because about 10% of the population was missing these variables and we wanted to see whether excluding these variables produced similar regression results for the predictors of interest to when they were included. For models 3 and 4, we included gestational age and size for gestation separately, to compare with models 1 and 2 respectively. We ran each of these 4 models twice, one time where we used weights and associated SGA/preterm taken within 72 hours of birth, and again using the multiply imputed weights and imputed SGA. This allowed us to compare how the imputation impacted the regression coefficients. Since some women had more than one pregnancy included in the analysis, we accounted for correlation within women using robust variance estimation (18).

RESULTS

A total of 32,004 live-born babies were included in this analysis. **Figure 1** shows the number of pregnancies identified, enrolled, and their outcomes (not including imputed birthweight). The baseline characteristics, neonatal mortality, and cHR of each potential risk factor is shown in **Table 1**.

For peer review only

Table 1 Characteristics, Neonatal Mortality and Crude Hazard Ratio of Potential Risk Factors (N=32,004)

Variables	Study Population (N=32,004)			Neonatal Mortality (per 1,000)	Crude Hazard Ratio	95% CI
	Deaths	Total	Distribu-tion %			
**Maternal Age (years) at LMP**						
Mean, 95% CI: 22.46 (22.41, 22.51)						
<=18	218	4,948	15.46	44.06	1.57	(1.34, 1.83)
18-35	748	26,351	82.34	28.39	1.00	Reference
>35	32	703	2.20	45.52	1.62	(1.14, 2.31)
Missing	0	2		0.00		
**Maternal Height (cm)**						
Mean, 95% CI: 150.55 (150.49, 150.61)						
<145	232	4,703	14.72	49.33	1.00	Reference
145-150	332	9,587	30.00	34.64	0.70	(0.59, 0.83)
>=150	429	17,664	55.28	24.29	0.49	(0.41, 0.57)
Missing	5	50		100.00		
**Maternal Education (years)**						
Mean, 95% CI: 2.61 (2.56, 2.65)						
No schooling	748	21,557	67.42	34.70	1.00	Reference
1-5	77	2,721	8.51	28.30	0.81	(0.64, 1.03)
>5	173	7,695	24.07	22.48	0.64	(0.54, 0.76)
Missing	0	31		0.00		
**Wealth Quintile**						
Poorest	262	6,534	20.43	40.10	1.00	Reference
Poorer	223	6,407	20.03	34.81	0.87	(0.72, 1.05)
Middle	206	6,400	20.01	32.19	0.80	(0.66, 0.97)
Richer	154	6,303	19.71	24.43	0.60	(0.49, 0.74)
Richest	152	6,339	19.82	23.98	0.59	(0.48, 0.73)
Missing	1	21		47.62		
**Caste of the Family**						
Brahmin and Chhetri	26	965	3.02	26.94	1.00	Reference
Vaishya	682	23,062	72.13	29.57	1.10	(0.74, 1.63)
Shudra	208	4,947	15.47	42.05	1.57	(1.05, 2.37)
Muslim and others	81	2,999	9.38	27.01	1.01	(0.65, 1.57)
Missing	1	31		32.26		
**Parity**						
1-4	523	20,444	64.22	25.58	1.00	Reference
>=5	62	1,398	4.39	44.35	1.75	(1.33, 2.30)
Prior pregnancy but no parity	35	791	2.48	44.25	1.75	(1.24, 2.47)
No prior pregnancy	369	9,199	28.90	40.11	1.58	(1.38, 1.82)
Missing	9	172		52.33		
**Gravidity**						
>=1	629	22,804	71.26	27.58	1.00	Reference
0 (First pregnancy)	369	9,199	28.74	40.11	1.46	(1.28, 1.67)
Missing	0	1		0.00		
**Last pregnancy outcome**						
No prior pregnancy	369	9,199	28.76	40.11	1.53	(1.34, 1.76)
At least one live birth	535	20,284	63.43	26.38	1.00	Reference
Stillbirth	19	532	1.66	35.71	1.36	(0.86, 2.14)
Miscarriage and abortion	75	1,965	6.14	38.17	1.46	(1.13, 1.89)
Missing	0	24		0.00		
**Prior child deaths**						
No prior pregnancy	369	9,199	29.19	40.11	1.76	(1.52, 2.04)
Prior live births but no child deaths	405	17,597	55.83	23.02	1.00	Reference
Prior live births and child death	149	3,641	11.55	40.92	1.80	(1.48, 2.18)
Prior pregnancy but no live birth	45	1,081	3.43	41.63	1.83	(1.33, 2.53)
Missing	30	486		61.73		
**Any prior still birth**						
No prior pregnancy	369	9,199	28.76	40.11	1.52	(1.32, 1.74)
Prior pregnancy but no still births	571	21,394	66.88	26.69	1.00	Reference
Prior still births	58	1,394	4.36	41.61	1.58	(1.18, 2.10)
Missing	0	17		0.00		
**Any prior miscarriage**						
No prior pregnancy	369	9,199	28.75	40.11	1.54	(1.34, 1.77)
Prior pregnancy but no miscarriage	503	19,142	59.83	26.28	1.00	Reference
Prior miscarriage	126	3,652	11.41	34.50	1.32	(1.08, 1.62)
Missing	0	11		0.00		
**Any prior multiple births**						
No prior pregnancy	369	9,199	28.76	40.11	1.49	(1.30, 1.70)
Prior pregnancy but no multiples	611	22,484	70.30	27.17	1.00	Reference
Prior multiples	18	299	0.93	60.20	2.25	(1.38, 3.68)
Missing	0	22		0.00		
**Tetanus vaccination in the past 2 years**						
No	193	5,066	15.83	38.10	1.00	Reference
Yes	805	26,938	84.17	29.88	0.78	(0.66, 0.92)
Missing	0	0				
**Any tobacco use in pregnancy**						
No	987	31,649	98.89	31.19	1.00	Reference
Yes	11	355	1.11	30.99	0.99	(0.55, 1.80)
Missing	0	0				
**Any alcohol use in pregnancy**						
No	997	31,908	99.70	31.34	1.00	Reference

For peer review only

Yes	1	96	0.30	10.42	0.33	(0.05, 2.37)
Missing	0	0				
**Place of delivery**						
At home or Maiti*	437	15,872	55.46	27.53	1.00	Reference
At health post/clinic or in hospital	405	12,130	42.38	33.39	1.22	(1.06, 1.40)
On the way to facility or outdoors	40	617	2.16	64.83	2.43	(1.70, 3.48)
Missing	116	3,385		34.27		
**Number of Antenatal Care (ANC) visits**						
0	184	5,559	19.46	33.10	1.00	Reference
1	148	4,173	14.61	35.47	1.07	(0.86, 1.34)
2 or 3	366	9,872	34.55	37.07	1.12	(0.93, 1.35)
>=4	183	8,967	31.38	20.41	0.61	(0.50, 0.76)
Missing	117	3,433		34.08		
**Intervention**						
Mustard Oil	520	16,327	51.02	31.85	1.00	Reference
Sunflower Oil	478	15,676	48.98	30.49	0.95	(0.84, 1.09)
Missing	0	1		0		
**Sex of Child**						
Male	548	16,531	51.73	26.63	1.00	Reference
Female	449	15,424	48.27	29.91	0.88	(0.77, 0.99)
Missing	1	49		20.41		
**Birth Weight (within 72 hours)**						
Mean, 95%CI: 2773.19g (2766.95, 2779.44)						
VLBW (<1500g)	51	128	0.46	398.44	69.88	(49.58, 98.50)
LBW (1500-2500g)	199	7,859	28.02	25.32	3.43	(2.76, 4.26)
NBW (2500-4000g)	141	19,354	69.01	7.29	1.00	Reference
HBW (>4000g)	3	706	2.52	4.25	0.57	(0.18, 1.78)
Missing	601	3,957		151.88		
**Imputed Birth Weight**						
Mean, 95%CI: 2774.9g (2770.1, 2779.8)						
VLBW (<1500g)	65	158	0.00	412.98	25.94	(19.38, 34.73)
LBW (1500-2500g)	491	8,298	26.00	59.22	3.23	(2.65, 3.94)
NBW (2500-4000g)	437	23,355	73.17	18.69	1.00	Reference
HBW (>4000g)	4	110	0.00	32.72	1.74	(0.59, 5.14)
Missing	1	83		12.05		
**Birth Weight (within 72 hours)**						
LBW (<2500g)	250	7,987	28.48	31.30	4.32	(3.51, 5.33)
Normal BW (>=2500g)	147	20,060	71.52	7.33	1.00	Reference
Missing	601	3,957		151.88		
**Imputed Birth Weight**						
LBW (<2500g)	556	8,456	26.49	65.77	3.59	(2.99, 4.32)
Normal BW (>=2500g)	440	23,465	73.51	18.76	1.00	Reference
Missing	1	83		12.05		
**Gestational Age (days)**						
Mean, 95%CI: 275.74 (275.49,275.99)						
Very preterm (<32 weeks)	193	662	2.12	291.54	17.44	(14.54, 20.91)
Moderate to late preterm (32-37 weeks)	224	4,244	13.62	52.78	2.66	(2.25, 3.14)
Term (37-42 weeks)	451	22,371	71.78	20.16	1.00	Reference
Post-term (42-45 weeks)	111	3,887	12.47	28.56	1.42	(1.16, 1.75)
Missing	0	3		0.00		
**Gestational Age**						
Preterm (<37 weeks)	417	4,906	15.74	85.00	4.12	(3.60, 4.71)
Term (37-45 weeks)	562	26,258	84.26	21.40	1.00	Reference
Missing	0	3		0.00		
**Size for Gestational Age (within 72 hours)**						
AGA	140	13,172	47.01	10.69	1.00	Reference
SGA	244	13,060	46.61	18.54	1.76	(1.42, 2.18)
LGA	12	1,789	6.38	6.83	0.63	(0.35, 1.12)
Missing	602	3,983		148.36		
**Imputed Size for Gestational Age**						
AGA	373	16,127	50.57	23.15	1.00	Reference
SGA	471	14,275	44.77	32.96	1.43	(1.10, 1.87)
LGA	147	1,486	4.66	98.95	4.52	(3.49, 5.85)
Missing	7	116		60.34		
**SGA/Preterm (within 72 hours)**						
AGA/LGA and Term/Post-term	70	11,381	41.64	6.15	1.00	Reference
SGA and Term/Post-term	185	11,859	43.39	15.60	2.55	(1.93, 3.37)
AGA/LGA and Preterm	81	3,437	12.58	23.57	3.86	(2.76, 5.39)
SGA and Preterm	54	652	2.39	82.82	13.95	(9.67, 20.13)
Missing	608	4,675		130.05		
**Imputed SGA/Preterm**						
AGA/LGA and Term/Post-term	189	13,413	43.42	14.06	1.00	Reference
SGA and Term/Post-term	391	13,607	41.32	28.76	2.06	(1.50, 2.83)
AGA/LGA and Preterm	324	4,175	13.30	77.55	5.73	(4.56, 7.21)
SGA and Preterm	79	669	1.97	118.38	8.82	(5.85, 13.28)
Missing	15	140		60.34		
**Singleton/Twin/Triplet**						
Singleton	892	31,502	98.43	28.32	1.00	Reference
Twin or Triplet	106	502	1.57	211.16	8.31	(6.43, 10.74)
Missing	0	0				

* Maiti is the maternal home, where women may go to deliver, especially in the first pregnancy

For peer review only - http://bmjopen.bmj.com/site/about/guidelines.xhtml

For peer review only

Based on the crude hazard ratios, maternal height, education, wealth quintile, caste, parity, gravidity, prior child deaths, and prior adverse pregnancy outcomes, place of delivery, number of ANC visits, multiple births, birthweight, SGA and preterm were all significantly associated with neonatal mortality. Birthweight imputation altered the cHRs for high birthweight (> 4000g) from being a risk factor to being protective and LGA (> 90th percentile) from no association to being a significant risk factor. While being SGA and preterm had a large and significant hazard ratio in the analysis without imputed weight values, this hazard ratio increased significantly when birthweights were imputed.

Variables with high proportions of missing data (around 10%) were ANC visits (10.7%), place of delivery (10.6%), birth weight (12.4%) and weights taken after 72 hours (13.5%). Neonatal mortality was higher among women who delivered at a facility or on their way to a facility compared to home births. Women who attended 4 or more ANC visits had lower neonatal mortality risk. Both before and after imputation, very low birth weight and low birth weight significantly increased neonatal mortality. Preterm or post-term resulted in higher neonatal mortality. SGA and LGA babies were at higher risk compared to AGA ones. Alcohol and tobacco use was not included in multivariate models, given the low prevalence of their use in pregnancy.

Multivariable regression results are shown in **Table 2** and **Table 3**. These models did not include gravidity, last pregnancy outcome, any prior pregnancy ending in stillbirth, miscarriage and multiples, and tobacco and alcohol use. Model 1 included place of delivery and number of ANC visits, while model 2 did not.

The total number of live births in the analysis was 26,626 with original data (weights taken within 72 hours), and 27,819 with imputed birthweight and SGA data in model 1 (**Table 2**). Place of delivery had a significant impact on neonatal mortality: the risks were higher both at health posts/clinics or in hospital [aHR: 1.34, 95% CI: (1.13, 1.58)] and on the way to the facility or outdoors [aHR: 2.26, 95% CI: (1.57, 3.26)]; the number of ANC visits was statistically significant for four or more visits [aHR: 0.67, 95% CI: (0.53, 0.86)].

In model 2 (**Table 3**), the number of participants included was 26,680 with weights taken within 72 hours, and 31,116 after birthweight imputation. The difference was due to more than 10% missing values for place of delivery and number of ANC visits, which were included in model 1, but not in model 2. Results for models 1 and 2 are quite similar, indicating that exclusion of place of delivery and ANC did not change the associations of the other risk factors with mortality.

For peer review only - http://bmjopen.bmj.com/site/about/guidelines.xhtml

Table 2 Multivariate Cox Regression Model 1

Variables	Crude Hazard Ratio	95% CI	**Model 1:** Maternal Age, Mother's Height, Mother's Years of Education, Wealth Quintile, Caste, Parity, Prior Child Deaths, Tetanus Vaccination, Place of Delivery, Number of ANC Visits, Sex of Child, SGA/Preterm, Imputed SGA/Preterm, Singleton/Twin+ Triplet			
			Adjusted Hazard Ratio (N=26,626)	95% CI	Adjusted HR (Using imputed SGA) (N=27,819)	95% CI
**Maternal Age (years) at LMP**						
Mean, 95% CI: 22.46 (22.41, 22.51)						
<=18	1.57	(1.34, 1.83)	1.39	(1.04, 1.86)	1.22	(1.01, 1.50)
18-35	1.00	Reference	1.00	Reference	1.00	Reference
>35	1.62	(1.14, 2.31)	1.28	(0.60, 2.77)	1.36	(0.84, 2.21)
**Maternal Height (cm)**						
Mean, 95% CI: 150.55 (150.49, 150.61)						
<145	1.00	Reference	1.00	Reference	1.00	Reference
145-150	0.70	(0.59, 0.83)	0.64	(0.48, 0.86)	0.75	(0.62, 0.92)
150	0.49	(0.41, 0.57)	0.57	(0.43, 0.75)	0.55	(0.46, 0.67)
**Maternal Education (years)**						
Mean, 95% CI: 2.61 (2.56, 2.65)						
No schooling	1.00	Reference	1.00	Reference	1.00	Reference
1-5	0.81	(0.64, 1.03)	1.01	(0.69, 1.49)	0.92	(0.71,1.20)
>5	0.64	(0.54, 0.76)	0.79	(0.57, 1.09)	0.78	(0.62, 0.97)
**Wealth Quintile**						
Poorest	1.00	Reference	1.00	Reference	1.00	Reference
Poorer	0.87	(0.72, 1.05)	0.85	(0.61, 1.17)	0.92	(0.74, 1.13)
Middle	0.80	(0.66, 0.97)	0.96	(0.69, 1.33)	0.95	(0.76, 1.19)
Richer	0.60	(0.49, 0.74)	0.73	(0.50, 1.06)	0.82	(0.64, 1.05)
Richest	0.59	(0.48, 0.73)	0.89	(0.60, 1.32)	0.91	(0.70, 1.18)
**Caste of the Family**						
Brahmin and Chhetri	1.00	Reference	1.00	Reference	1.00	Reference
Vaishya	1.10	(0.74, 1.63)	1.29	(0.57, 2.91)	0.86	(0.55, 1.34)
Shudra	1.57	(1.05, 2.37)	1.61	(0.69, 3.76)	1.01	(0.63, 1.64)
Muslim and others	1.01	(0.65, 1.57)	1.04	(0.43, 2.52)	0.69	(0.41, 1.14)
**Parity**						
1-4	1.00	Reference	1.00	Reference	1.00	Reference
>=5	1.75	(1.33, 2.30)	1.20	(0.66, 2.19)	1.04	(0.70, 1.55)
Prior pregnancy but no parity	1.75	(1.24, 2.47)	1.99	(0.43, 9.11)	1.63	(0.69, 3.84)
No prior pregnancy	1.58	(1.38, 1.82)	1.43	(1.06, 1.92)	1.73	(1.42, 2.12)
**Prior child deaths**						
No prior pregnancy	1.76	(1.52, 2.04)	Omitted		Omitted	
Prior live births but no child deaths	1.00	Reference	1.00	Reference	1.00	Reference
Prior live births and child death	1.80	(1.48, 2.18)	1.11	(0.79, 1.55)	1.56	(1.26, 1.95)
Prior pregnancy but no live birth	1.83	(1.33, 2.53)	0.71	(0.17, 2.93)	1.28	(0.57, 2.88)
**Tetanus vaccination in the past 2 years**						
No	1.00	Reference	1.00	Reference	1.00	Reference
Yes	0.78	(0.66, 0.92)	0.84	(0.63, 1.11)	0.74	(0.62, 0.89)
**Place of delivery**						
At home or Maiti*	1.00	Reference	1.00	Reference	1.00	Reference
At health post/clinic or in hospital	1.22	(1.06, 1.40)	0.94	(0.73, 1.21)	1.34	(1.13, 1.58)
On the way to facility or outdoors	2.43	(1.70, 3.48)	1.42	(0.72, 2.81)	2.26	(1.57, 3.26)
**Number of Antenatal Care (ANC)**						
0	1.00	Reference	1.00	Reference	1.00	Reference
1	1.07	(0.86, 1.34)	1.03	(0.73, 1.47)	1.07	(0.85, 1.34)
2 or 3	1.12	(0.93, 1.35)	1.06	(0.79, 1.42)	1.06	(0.87, 1.29)
>=4	0.61	(0.50, 0.76)	0.77	(0.54, 1.11)	0.67	(0.53, 0.86)
**Sex of Child**						
Male	1.00	Reference	1.00	Reference	1.00	Reference
Female	0.88	(0.77, 0.99)	1.16	(0.94, 1.43)	0.92	(0.80, 1.06)
**SGA/Preterm (within 72 hours)**						
AGA/LGA and Term/Post-term	1.00	Reference	1.00	Reference		
SGA and Term/Post-term	2.55	(1.93, 3.37)	2.11	(1.56, 2.83)		
AGA/LGA and Preterm	3.86	(2.76, 5.39)	3.11	(2.22, 4.35)		
SGA and Preterm	13.95	(9.67, 20.13)	6.90	(4.33, 10.97)		
**Imputed SGA/Preterm**						
AGA/LGA and Term/Post-term	1.00	Reference			1.00	Reference
SGA and Term/Post-term	2.06	(1.50, 2.83)			1.67	(1.10, 2.54)
AGA/LGA and Preterm	5.73	(4.56, 7.21)			4.26	(3.20, 5.66)
SGA and Preterm	8.82	(5.85, 13.28)			3.92	(2.18, 7.07)
**Singleton/Twin/Triplet**						
Singleton	1.00	Reference	1.00	Reference	1.00	Reference
Twin or Triplet	8.31	(6.43, 10.74)	4.89	(2.91, 8.21)	5.43	(4.01, 7.37)

* Maiti is the maternal home, where women may go to deliver, especially in the first pregnancy

For peer review only

For peer review only - http://bmjopen.bmj.com/site/about/guidelines.xhtml

Table 3 Multivariate Cox Regression Model 2

Variables	Crude Hazard Ratio	95% CI	**Model 2**: Maternal Age, Mother's Height, Mother's Years of Education, Wealth Quintile, Caste, Parity, Prior Child Death, Tetanus Vaccination, Sex of Child, SGA/Preterm, Imputed SGA/Preterm, Singleton/Twin/Triplet			
			**Adjusted Hazard Ratio (N=26,680)**	**95% CI**	**Adjusted HR (Using imputed SGA) (N=31,116)**	**95% CI**
**Maternal Age (years) at LMP**						
Mean, 95% CI: 22.46 (22.41, 22.51)						
<=18	1.57	(1.34, 1.83)	1.41	(1.05, 1.88)	1.15	(0.95, 1.38)
18-35	1.00	Reference	1.00	Reference	1.00	Reference
>35	1.62	(1.14, 2.31)	1.26	(0.58, 2.71)	1.46	(0.93, 2.28)
**Maternal Height (cm)**						
Mean, 95% CI: 150.55 (150.49, 150.61)						
<145	1.00	Reference	1.00	Reference	1.00	Reference
145-150	0.70	(0.59, 0.83)	0.64	(0.48, 0.85)	0.78	(0.65, 0.94)
>=150	0.49	(0.41, 0.57)	0.57	(0.43, 0.75)	0.57	(0.47, 0.68)
**Maternal Education (years)**						
Mean, 95% CI: 2.61 (2.56, 2.65)						
No schooling	1.00	Reference	1.00	Reference	1.00	Reference
1-5	0.81	(0.64, 1.03)	0.98	(0.67, 1.44)	0.89	(0.69, 1.14)
>5	0.64	(0.54, 0.76)	0.75	(0.55, 1.04)	0.75	(0.62, 0.92)
**Wealth Quintile**						
Poorest	1.00	Reference	1.00	Reference	1.00	Reference
Poorer	0.87	(0.72, 1.05)	0.84	(0.61, 1.17)	0.96	(0.79, 1.18)
Middle	0.80	(0.66, 0.97)	0.94	(0.68, 1.30)	0.94	(0.76, 1.16)
Richer	0.60	(0.49, 0.74)	0.71	(0.49, 1.03)	0.82	(0.66, 1.04)
Richest	0.59	(0.48, 0.73)	0.87	(0.59, 1.29)	0.92	(0.72, 1.17)
**Caste of the Family**						
Brahmin and Chhetri	1.00	Reference	1.00	Reference	1.00	Reference
Vaishya	1.10	(0.74, 1.63)	1.34	(0.59, 3.03)	0.86	(0.57, 1.31)
Shudra	1.57	(1.05, 2.37)	1.68	(0.72, 3.93)	1.00	(0.64, 1.57)
Muslim and others	1.01	(0.65, 1.57)	1.08	(0.44, 2.62)	0.67	(0.41, 1.07)
**Parity**						
1-4	1.00	Reference	1.00	Reference	1.00	Reference
>=5	1.75	(1.33, 2.30)	1.21	(0.67, 2.21)	1.05	(0.72, 1.52)
Prior pregnancy but no parity	1.75	(1.24, 2.47)	2.01	(0.44, 9.24)	1.52	(0.71, 3.27)
No prior pregnancy	1.58	(1.38, 1.82)	1.39	(1.05, 1.85)	1.79	(1.50, 2.15)
**Prior child deaths**						
No prior pregnancy	1.76	(1.52, 2.04)	Omitted		Omitted	
Prior live births but no child death	1.00	Reference	1.00	Reference	1.00	Reference
Prior pregnancy but no live births	1.80	(1.48, 2.18)	0.67	(0.16, 2.77)	1.32	(0.65, 2.67)
Prior live births and child death	1.83	(1.33, 2.53)	1.11	(0.79, 1.56)	1.53	(1.24, 1.87)
**Tetanus Vaccination in the past 2 years**						
No	1.00	Reference	1.00	Reference	1.00	Reference
Yes	0.78	(0.66, 0.92)	0.84	(0.63, 1.10)	0.88	(0.74, 1.04)
**Sex of Child**						
Male	1.00	Reference	1.00	Reference	1.00	Reference
Female	0.88	(0.77, 0.99)	1.17	(0.95, 1.45)	0.89	(0.78, 1.01)
**SGA/Preterm (within 72 hours)**						
AGA/LGA and Term/Post-term	1.00	Reference	1.00	Reference		
SGA and Term/Post-term	2.55	(1.93, 3.37)	2.12	(1.58, 2.86)		
AGA/LGA and Preterm	3.86	(2.76, 5.39)	3.23	(2.30, 4.54)		
SGA and Preterm	13.95	(9.67, 20.13)	7.09	(4.44 11.31)		
**Imputed SGA/Preterm**						
AGA/LGA and Term/Post-term	1.00	Reference			1.00	Reference
SGA and Term/Post-term	2.06	(1.50, 2.83)			1.68	(1.20, 2.35)
AGA/LGA and Preterm	5.73	(4.56, 7.21)			4.50	(3.51, 5.77)
SGA and Preterm	8.82	(5.85, 13.28)			4.11	(3.52, 6.69)
**Singleton/Twin/Triplet**						
Singleton	1.00	Reference	1.00	Reference	1.00	Reference
Twin or Triplet	8.31	(6.43, 10.74)	4.76	(2.84, 8.00)	5.64	(4.25, 7.48)

For peer review only

After imputation and adjustment, variables such as maternal age, wealth quintile, and caste of the family were not significantly associated with neonatal mortality. Increasing maternal height from <145 cm to 145-150 cm [aHR: 0.78, 95% CI: (0.65, 0.94)] to ≥150 cm [aHR: 0.57, 95% CI: (0.47, 0.68)] and education to >5 years [aHR: 0.75, 95% CI: (0.62, 0.92)] remained protective factors. Parity was not significantly associated with neonatal mortality. Prior live births and child death [aHR: 1.53, 95% CI: (1.24, 1.87)] were associated with higher risk. Tetanus vaccination did not have a significant impact on neonatal mortality. Being a twin or triplet [aHR:5.64, 95% CI: (4.25, 7.48)] was also significantly associated with neonatal mortality. The findings were similar in models 1 and 2.

Any SGA and/or preterm was strongly associated with an increased likelihood of neonatal mortality (**Figure 2**): SGA and preterm [aHR: 7.09, 95% CI: (4.44, 11.31)], SGA and term/post-term [aHR: 2.12, 95% CI: (1.58, 2.86)], AGA/LGA and preterm [aHR: 3.23, 95% CI: (2.30, 4.54)]. Imputation of birthweights increased the association between AGA/LGA and preterm and mortality but not the other categories of adverse birth outcomes.

We also created two other multivariate regression models, model S1 and S2 as shown in **Table S1** and **Table S2** in the appendices. These included gestational age and size for gestational age as individual variables.

DISCUSSION

Nepal was one of the few countries to meet the Millennium Development Goal 4 for child mortality but neonatal mortality rates are still high and need to be reduced by twofold to meet the sustainable development goals of 12 per 1000 live births(19,20). This study identified maternal characteristics (maternal height, education, and prior child death), neonatal characteristics (SGA, preterm, and twin or triplet), delivery and health care (place of delivery and number of ANC visits) as significant risk factors for neonatal mortality in rural southern Nepal.

According to previous existing studies, maternal age(7,21,22), maternal height(23), maternal education(8,9,15), wealth(9,21), low birth weight(22,24,25), SGA and preterm(22,26–28), twins(9,26,29) and triplets(29) were all strongly associated with neonatal deaths, which corresponds to our findings. Health care services, specifically the number of ANC(6,12) visits and place of delivery(13) also has an impact on neonatal deaths. In our study, neonatal mortality was higher among those delivering in a facility, outdoors or on the way to a facility than at home. This is likely due to some women in this population starting their delivery at home, and only going to a facility if something goes wrong during labor and delivery, or being referred to the facility because they were high risk. As the proportion of routine deliveries at facilities

For peer review only - http://bmjopen.bmj.com/site/about/guidelines.xhtml

increases, this association may reverse. Previous studies suggested that parity was not considered a predictor of neonatal mortality(30), which was also supported by our analysis.

A systematic review indicated that neonatal mortality is reduced by vaccination of women of childbearing age with tetanus toxoid in LMICs(11), while we found no association with mortality both before and after adjustment. Neonatal tetanus is an unlikely cause of neonatal deaths due to the high rate of tetanus toxoid vaccination among women of childbearing age in Nepal. A recent programmatic focus on vaccinating adolescent girls with tetanus toxoid may also explain this lack of association. Tetanus toxoid vaccine receipt during pregnancy (as collected in this study) is often used as a proxy for ANC utilization in LMICs, but we included the number of ANC visits in the regressions and found it to be associated with neonatal mortality. ANC visits are likely a more proximate risk factor for mortality than tetanus toxoid receipt.

Strengths of this study included a large cohort of pregnancies followed through 28 days postpartum with up to eight visits in the neonatal period. The frequent visits reduced loss to follow up and improved estimates of age at death. While gestational age was not measured using gold standard ultrasound, the date of last menstrual period was obtained early in the pregnancy, limiting recall bias. However, the reliance on date of last menstrual period could have led to some misclassification of preterm births. Another strength of this study was that stillbirths were identified using maternal recall of whether the baby moved, cried or breathed after birth. If the infant did none of these, they were classified as a stillbirth. However, there could be some misclassification of stillbirths as live births and vice versa. Some other limitations include many missing weights due to early newborn death or weighing of the baby 72 hours or more after birth. Most of the missing values for birth weight were estimated by using multiple imputation, which increased the uncertainty of this analysis. The imputation of birth weights also had a qualitative impact on the regression coefficients, in particular for the AGA/LGA and preterm category, which went from an adjusted hazard ratio of 3.13 to one of 5.46. This may be due to the fact that preterm infants who died soon after birth were likely to be missing weights. The neonatal mortality rate among preterm births was 33.02 per 1,000 before imputation and 83.19 per 1,000 after imputation. Similarly, the neonatal mortality rate among SGA before imputation was 19.10 per 1,000 and 32.92 per 1,000 after imputation.

In addition to missing birth weights, about 10% of values were missing for place of delivery and number of ANC visits. We conducted sensitivity analyses including and excluding these covariates and found that the other regression coefficients were not altered significantly by their being included.

Our study found that maternal age, wealth quintile, caste of the family, parity, and tetanus vaccination were not significantly associated with neonatal mortality after adjustment and imputation. Although these findings conflict with some of the existing literature, it is possible that the inclusion of more proximate risk factors

For peer review only - http://bmjopen.bmj.com/site/about/guidelines.xhtml

such as gestational age and size for gestational age lessened the importance of these other more distal risk factors.

CONCLUSION

Maternal height, maternal education, prior child deaths, SGA, preterm, and twins or triplets, place of delivery and number of ANC visits were significantly associated with neonatal mortality risk. Interventions that focus on preventing SGA and preterm births, improving education for women, promoting quality antenatal health care and facility delivery, and care of vulnerable infants after birth should continue to be emphasized and prioritized.

Contributors

JK conceived the idea for this paper. SS and EH managed the dataset. TY conducted the analysis. All authors contributed to the interpretation of the findings. TY and JK jointly wrote the first draft. All authors critically revised the paper for intellectual content and approved the final version of the manuscript.

Funding

This work was supported by the National Institute for Child Health and Human Development (R01HD092411 and R01HD060712) and the Bill & Melinda Gates Foundation (OPP1084399). The funders did not have a role in the design of the study, the data collection, nor the analysis, interpretation and writing of the manuscript.

Disclaimers

The views expressed in the submitted article are the authors' own and not an official position of the institution or funder.

Competing Interests

Each author has filled ICMJE form for competing interests disclosure. The authors have no conflicts of interest to declare.

Ethics Approval

This analysis of secondary data was considered exempt by the Johns Hopkins Bloomberg School of Public Health institutional review board (IRB) (FWA00000287). The original NOMS study received IRB approval the Johns Hopkins Bloomberg School of Public Health and Tribhuvan University Institute of Medicine in Nepal.

Data Sharing

For peer review only

No additional data are available.

REFERENCES

1. Wang H, Liddell CA, Coates MM, Mooney MD, Levitz CE, Schumacher AE, et al. Global, regional, and national levels of neonatal, infant, and under-5 mortality during 1990-2013: A systematic analysis for the Global Burden of Disease Study 2013. The Lancet. 2014;384(9947).
2. Ministry of Health Nepal, ICF, New ERA. 2016 Nepal Demographic and Health Survey Key Findings. Kathmandu, Nepal: Ministry of Health Nepal. 2017;1.
3. Paudel D, Thapa A, Shedain PR, Paudel B. Trends and determinants of neonatal mortality in Nepal: further analysis of the Nepal Demographic and Health Surveys, 2001-2011. DHS Further Analysis Report. 2013.
4. GoN/NPC. National Planning Commission, 2015: Sustainable Development Goals, 2016-2030, National (Preliminary) Report. Government of Nepal, National Planning Commission, Kathmandu, Nepal. 2015. 39–52 p.
5. Kamal SMM, Ashrafuzzaman M, Narreen SA. Risk factors of neonatal mortality in Bangladesh. Journal of Nepal Paediatric Society. 2012;32(1).
6. Batieha AM, Khader YS, Berdzuli N, Chua-Oon C, Badran EF, Al-sheyab NA, et al. Level, causes and risk factors of neonatal mortality, in jordan: results of a national prospective study. Maternal and Child Health Journal. 2016;20(5).
7. Sharma V, Katz J, Mullany LC, Khatry SK, LeClerq SC, Shrestha SR, et al. Young maternal age and the risk of neonatal mortality in rural Nepal. Archives of Pediatrics and Adolescent Medicine. 2008;162(9).
8. Kamal SMM. Maternal education as a determinant of neonatal mortality in Bangladesh. Journal of Health Management. 2012;14(3).
9. McKinnon B, Harper S, Kaufman JS, Bergevin Y. Socioeconomic inequality in neonatal mortality in countries of low and middle income: A multicountry analysis. The Lancet Global Health. 2014;2(3).
10. Katz J, West KP, Khatry SK, Christian P, LeClerq SC, Pradhan EK, et al. Risk factors for early infant mortality in Sarlahi district, Nepal. Bulletin of the World Health Organization. 2003;81(10).
11. Khan AA, Zahidie A, Rabbani F. Interventions to reduce neonatal mortality from neonatal tetanus in low- and middle-income countries - A systematic review. BMC Public Health. 2013;13(1).
12. Arunda M, Emmelin A, Asamoah BO. Effectiveness of antenatal care services in reducing neonatal mortality in Kenya: Analysis of national survey data. Global Health Action. 2017;10(1).
13. Ajaari J. Impact of Place of Delivery On Neonatal Mortality In Rural Tanzania. Value in Health. 2013;16(3).
14. Villar J, Ismail LC, Victora CG, Ohuma EO, Bertino E, Altman DG, et al. International standards for newborn weight, length, and head circumference by gestational age and sex: The Newborn Cross-Sectional Study of the INTERGROWTH-21st Project. The Lancet. 2014;384(9946).
15. StataCorp. Stata Statistical Software: Release 16. College Station, TX: StataCorp LLC. StataCorp LLC. 2019.
16. Siegel EH, Stoltzfus RJ, Khatry SK, LeClerq SC, Katz J, Tielsch JM. Epidemiology of anemia among 4- to 17-month-old children living in south central Nepal. European Journal of Clinical Nutrition. 2006;60(2).
17. Hazel E, Mullany LC, Zeger SL, Mohan D, Subedi S, Tielsch JM, Khatry SK, Katz J. Development of an imputation model to recalibrate birthweights measured in the early neonatal period to time at delivery and assessment of its impact on size-for-gestational age and low birthweight prevalence estimates: a secondary analysis of a pregnancy cohort in rural Nepal. BMJ Open 2022 (accepted for

For peer review only - http://bmjopen.bmj.com/site/about/guidelines.xhtml

publication)
18. Lin, Danyu Y., and Lee-Jen Wei. The robust inference for the Cox proportional hazards model. Journal of the American statistical Association 84.408 (1989): 1074-1078.
19. Boerma T, Requejo J, Victora CG, Amouzou A, George A, Agyepong I, et al. Countdown to 2030: tracking progress towards universal coverage for reproductive, maternal, newborn, and child health. Vol. 391, The Lancet. 2018.
20. Victora CG, Requejo JH, Barros AJD, Berman P, Bhutta Z, Boerma T, et al. Countdown to 2015: A decade of tracking progress for maternal, newborn, and child survival. Vol. 387, The Lancet. 2016.
21. Neupane S, Doku DT. Neonatal mortality in Nepal: A multilevel analysis of a nationally representative. Journal of Epidemiology and Global Health. 2014;4(3).
22. de Jesus LC, Pappas A, Shankaran S, Kendrick D, Das A, Higgins RD, et al. Risk factors for post-neonatal intensive care unit discharge mortality among extremely low birth weight infants. Journal of Pediatrics. 2012;161(1).
23. Khatun W, Alam A, Rasheed S, Huda TM, Dibley MJ. Exploring the intergenerational effects of undernutrition: Association of maternal height with neonatal, infant and under-five mortality in Bangladesh. BMJ Global Health. 2018;3(6).
24. Pulver LS, Guest-Warnick G, Stoddard GJ, Byington CL, Young PC. Weight for gestational age affects the mortality of late preterm infants. Pediatrics. 2009;123(6).
25. Yasmin S, Osrin D, Paul E, Costello A. Neonatal mortality of low-birth-weight infants in Bangladesh. Bulletin of the World Health Organization. 2001;79(7).
26. Blickstein I, Keith LG. Neonatal mortality rates among growth-discordant twins, classified according to the birth weight of the smaller twin. American Journal of Obstetrics and Gynecology. 2004;190(1).
27. Clausson B, Cnattingius S, Axelsson O. Preterm and term births of small for gestational age infants: A population-based study of risk factors among nulliparous women. BJOG: An International Journal of Obstetrics and Gynaecology. 1998;105(9).
28. Kozuki N, Lee AC, Silveira MF, Sania A, Vogel JP, Adair L, et al. The associations of parity and maternal age with small-for-gestational-age, preterm, and neonatal and infant mortality: A meta-analysis. Vol. 13, BMC Public Health. 2013.
29. Garite TJ, Clark RH, Elliott JP, Thorp JA. Twins and triplets: The effect of plurality and growth on neonatal outcome compared with singleton infants. American Journal of Obstetrics and Gynecology. 2004;191(3).
30. Wulandari RD, Laksono AD. Is parity a predictor of neonatal death in Indonesia Analysis of the 2017 Indonesia demographic and health survey. Indian Journal of Forensic Medicine and Toxicology. 2020;14(3).

Figure 1 Flow Diagram for Participants in NOMS Study

Figure 2 Cumulative Neonatal Mortality Curves by SGA/Preterm Excluding Imputed Data

For peer review only

Figure 1 Flow Diagram for Participants in NOMS Study

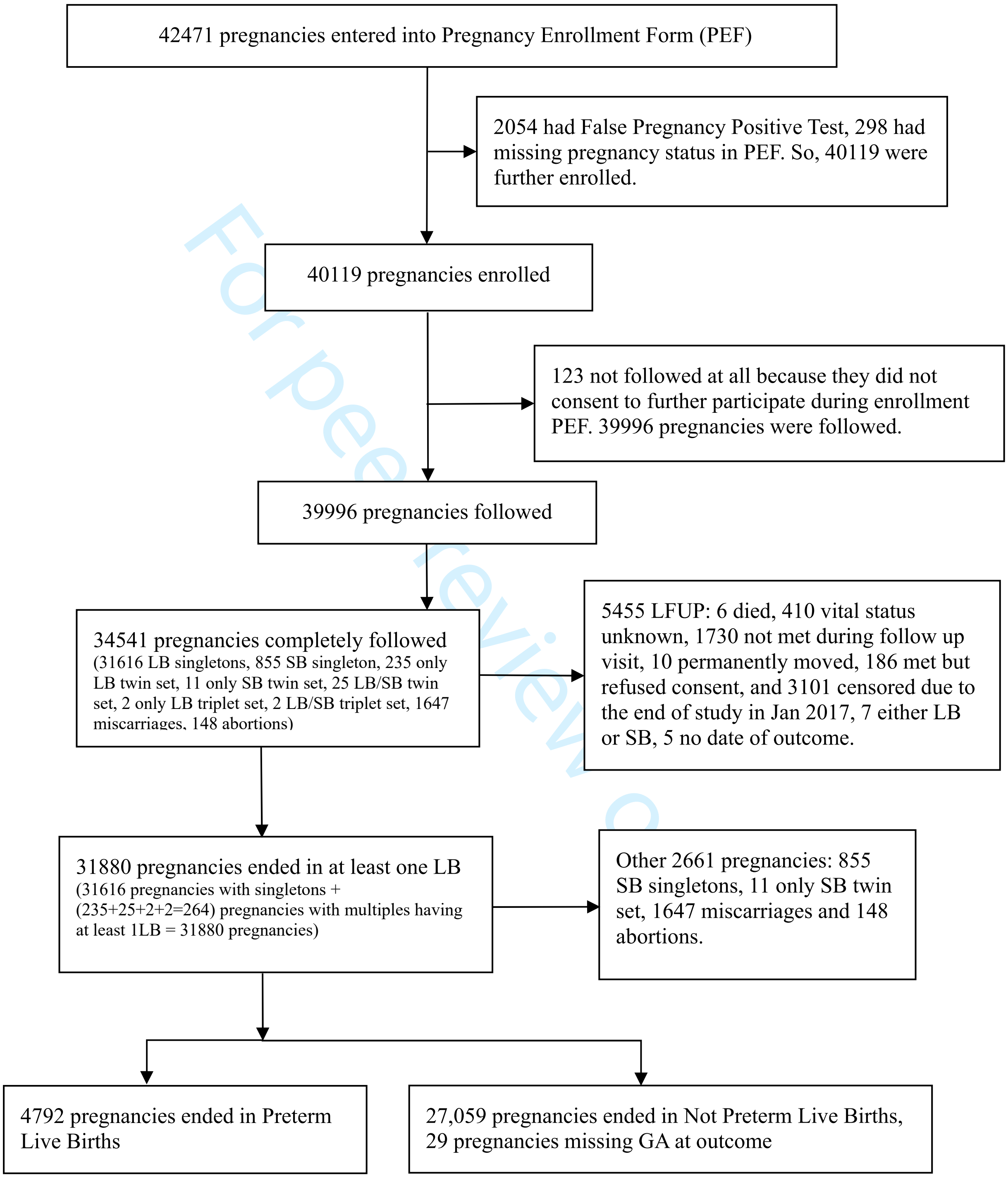

**Data collection was halted for 6 weeks in December 2016.*

For peer review only

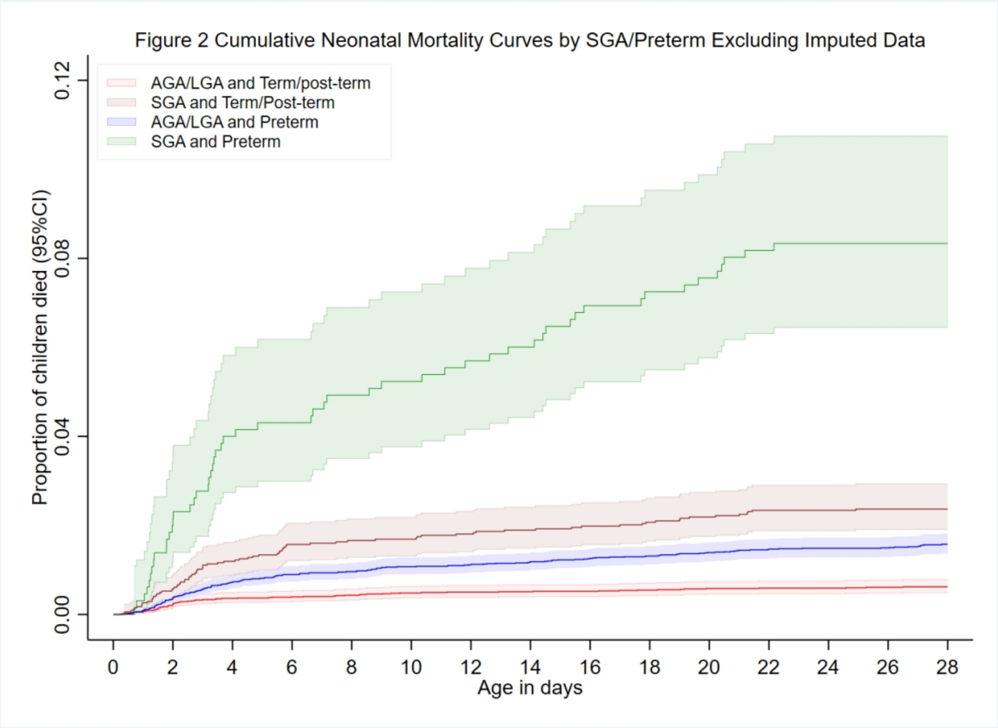

Figure 2 Cumulative Neonatal Mortality Curves by SGA/Preterm Excluding Imputed Data

489x355mm (72 x 72 DPI)

APPENDIX: SUPPLEMENTAL MATERIALS

Table S1 Multivariate Cox Regression Model S1

Variables	Crude Hazard Ratio	95% CI	**Model S1:** Maternal Age, Mother's Height, Mother's Years of Education, Wealth Quintile, Caste, Parity, Prior Child Deaths, Tetanus Vaccination, Place of Delivery, Number of ANC Visits, Sex of Child, Gestational Age, Size for Gestation, Singleton/Twin or Triplet			
			**Adjusted Hazard Ratio (N=26,626)**	**95% CI**	**Adjusted HR (Using imputed SGA) (N=27,158)**	**95% CI**
**Maternal Age (years) at LMP**						
Mean, 95% CI: 22.46 (22.41, 22.51)						
<=18	1.57	(1.34, 1.83)	1.34	(1.00, 1.76)	1.25	(1.02, 1.52)
18-35	1.00	Reference	1.00	Reference	1.00	Reference
>35	1.62	(1.14, 2.31)	1.39	(0.64, 3.04)	1.25	(0.74, 2.10)
**Maternal Height (cm)**						
Mean, 95% CI: 150.55 (150.49, 150.61)						
<145	1.00	Reference	1.00	Reference	1.00	Reference
145-150	0.70	(0.59, 0.83)	0.69	(0.52, 0.93)	0.82	(0.67, 1.00)
>=150	0.49	(0.41, 0.57)	0.63	(0.48, 0.84)	0.59	(0.49, 0.72)
**Maternal Education (years)**						
Mean, 95% CI: 2.61 (2.56, 2.65)						
0	1.00	Reference	1.00	Reference	1.00	Reference
1-5	0.81	(0.64, 1.03)	0.98	(0.67, 1.43)	0.95	(0.74, 1.24)
>5	0.64	(0.54, 0.76)	0.75	(0.53, 1.04)	0.77	(0.62, 0.97)
**Wealth Quintile**						
Poorest	1.00	Reference	1.00	Reference	1.00	Reference
Poorer	0.87	(0.72, 1.05)	0.92	(0.66, 1.27)	0.95	(0.76, 1.17)
Middle	0.80	(0.66, 0.97)	1.03	(0.74, 1.43)	1.00	(0.80, 1.25)
Richer	0.60	(0.49, 0.74)	0.82	(0.56, 1.19)	0.88	(0.69, 1.12)
Richest	0.59	(0.48, 0.73)	1.01	(0.68, 1.50)	1.00	(0.77, 1.31)
**Caste of the Family**						
Brahmin and Chhetri	1.00	Reference	1.00	Reference	1.00	Reference
Vaishya	1.10	(0.74, 1.63)	1.19	(0.53, 2.67)	0.86	(0.54, 1.37)
Shudra	1.57	(1.05, 2.37)	1.48	(0.63, 3.46)	1.00	(0.61, 1.64)
Muslim and others	1.01	(0.65, 1.57)	1.06	(0.44, 2.57)	0.73	(0.44, 1.23)
**Parity**						
1-4	1.00	Reference	1.00	Reference	1.00	Reference
>=5	1.75	(1.33, 2.30)	1.18	(0.65, 2.13)	1.02	(0.68, 1.53)
Prior pregnancy but no parity	1.75	(1.24, 2.47)	2.04	(0.45, 9.35)	1.50	(0.65, 3.46)
No prior pregnancy	1.58	(1.38, 1.82)	1.38	(1.02, 1.85)	1.68	(1.29, 1.93)
**Prior child death**						
No prior pregnancy	1.76	(1.52, 2.04)	Omitted		Omitted	
Prior live births but no deaths	1.00	Reference	1.00	Reference	1.49	(0.67, 3.29)
Prior live births and child death	1.80	(1.48, 2.18)	1.14	(0.82, 1.58)	1.00	Reference
Prior pregnancy but no live birth	1.83	(1.33, 2.53)	0.75	(0.18, 3.09)	1.55	(1.26, 1.91)
**Tetanus Vaccination in past 2 years**						
No	1.00	Reference	1.00	Reference	1.00	Reference
Yes	0.78	(0.66, 0.92)	0.84	(0.64, 1.11)	0.81	(0.67, 0.96)
**Place of Delivery**						
At home or Maiti*	1.00	Reference	1.00	Reference	1.00	Reference
At health post/clinic or in hospital	1.22	(1.06, 1.40)	0.92	(0.71, 1.18)	1.30	(1.10, 1.54)
On the way to facility or outdoors	2.43	(1.70, 3.48)	1.20	(0.66, 2.17)	2.08	(1.47, 2.95)
**Number of Antenatal Care (ANC)**						
0	1.00	Reference	1.00	Reference	1.00	Reference
1	1.07	(0.86, 1.34)	0.99	(0.70, 1.39)	1.05	(0.84, 1.31)
2 or 3	1.12	(0.93, 1.35)	1.04	(0.78, 1.39)	1.10	(0.91, 1.34)
>=4	0.61	(0.50, 0.76)	0.77	(0.54, 1.10)	0.70	(0.55, 0.90)
**Sex of Child**						
Male	1.00	Reference	1.00	Reference	1.00	Reference
Female	0.88	(0.77, 0.99)	1.16	(0.94, 1.43)	0.91	(0.80, 1.05)
**Gestational Age**						
Very preterm (<32 weeks)	17.44	(14.54, 20.91)	45.45	(27.63, 74.75)	20.7	(13.88, 30.86)
Moderate to late preterm (32-37 weeks)	2.66	(2.25, 3.14)	2.85	(2.11, 3.85)	2.59	(2.07, 3.25)
Term (37-42 weeks)	1.00	Reference	1.00	Reference	1.00	Reference
Post-term (>= 42 weeks)	1.42	(1.16, 1.75)	1.14	(0.82, 1.59)	1.25	(0.98, 1.59)
**Size for Gestational Age (within 72 hours)**						
AGA	1.00	Reference	1.00	Reference		
SGA	1.76	(1.42, 2.18)	2.30	(1.77, 3.00)		
LGA	0.63	(0.35, 1.12)	0.09	(0.04, 0.19)		
**Imputed Size for Gestational Age**						
AGA	1.00	Reference			1.00	Reference
SGA	1.43	(1.10, 1.87)			1.66	(1.10, 2.50)
LGA	4.52	(3.49, 5.85)			0.75	(0.52, 1.10)
**Singleton/Twin/Triplet**						
Singleton	1.00	Reference	1.00	Reference	1.00	Reference
Twin or Triplet	8.31	(6.43, 10.74)	3.26	(2.04, 5.21)	3.77	(2.75, 5.17)

* Maiti is the maternal home, where women may go to deliver, especially in the first pregnancy

Table S2 Multivariate Cox Regression Model S2

Variables	Crude Hazard Ratio	95% CI	**Model S2:** Maternal Age, Mother's Height, Mother's Years of Education, Wealth Quintile, Caste, Parity, Prior Child Death, Tetanus Vaccination, Sex of Child, Gestational Age, Size for Gestation, Singleton/Twin or Triplet			
			Adjusted Hazard Ratio (N=26,680)	95% CI	Adjusted HR (Using imputed SGA) (N=30,332)	95% CI
**Maternal Age (years) at LMP**						
Mean, 95% CI: 22.46 (22.41, 22.51)						
<=18	1.57	(1.34, 1.83)	1.35	(1.02, 1.80)	1.15	(0.96, 1.39)
18-35	1.00	Reference	1.00	Reference	1.00	Reference
>35	1.62	(1.14, 2.31)	1.36	(0.63, 2.93)	1.32	(0.82, 2.15)
**Maternal Height (cm)**						
Mean, 95% CI: 150.55 (150.49, 150.61)						
<145	1.00	Reference	1.00	Reference	1.00	Reference
145-150	0.70	(0.59, 0.83)	0.69	(0.51, 0.93)	0.83	(0.69, 1.01)
>=150	0.49	(0.41, 0.57)	0.63	(0.48, 0.84)	0.60	(0.50, 0.72)
**Maternal education (years)**						
Mean, 95% CI: 2.61 (2.56, 2.65)						
No schooling	1.00	Reference	1.00	Reference	1.00	Reference
1-5	0.81	(0.64, 1.03)	0.95	(0.65, 1.39)	0.92	(0.72, 1.17)
>5	0.64	(0.54, 0.76)	0.71	(0.51, 0.99)	0.75	(0.61, 0.93)
**Wealth Quintile**						
Poorest	1.00	Reference	1.00	Reference	1.00	Reference
Poorer	0.87	(0.72, 1.05)	0.91	(0.66, 1.25)	0.99	(0.81, 1.22)
Middle	0.80	(0.66, 0.97)	1.00	(0.72, 1.39)	0.99	(0.80, 1.22)
Richer	0.60	(0.49, 0.74)	0.79	(0.54, 1.15)	0.88	(0.70, 1.10)
Richest	0.59	(0.48, 0.73)	0.99	(0.67, 1.46)	0.99	(0.78, 1.27)
**Caste of the Family**						
Brahmin and Chhetri	1.00	Reference	1.00	Reference	1.00	Reference
Vaishya	1.10	(0.74, 1.63)	1.23	(0.54, 2.77)	0.88	(0.57, 1.34)
Shudra	1.57	(1.05, 2.37)	1.54	(0.66, 3.60)	0.99	(0.62, 1.56)
Muslim	1.01	(0.65, 1.57)	1.10	(0.45, 2.65)	0.70	(0.43, 1.13)
**Parity**						
1-4	1.00	Reference	1.00	Reference	1.00	Reference
>=5	1.75	(1.33, 2.30)	1.19	(0.66, 2.15)	1.03	(0.70, 1.51)
Prior pregnancy but no parity	1.75	(1.24, 2.47)	2.08	(0.45, 9.57)	1.38	(0.65, 2.93)
No prior pregnancy	1.58	(1.38, 1.82)	1.34	(1.01, 1.77)	1.65	(1.38, 1.98)
**Prior child deaths**						
No prior pregnancy	1.76	(1.52, 2.04)	Omitted		Omitted	
Prior live births but no deaths	1.00	Reference	1.00	Reference	1.00	Reference
Prior live births and child death	1.80	(1.48, 2.18)	1.14	(0.82, 1.59)	1.49	(1.22, 1.82)
Prior pregnancy but no live birth	1.83	(1.33, 2.53)	0.69	(0.17, 2.87)	1.54	(0.76, 3.11)
**Tetanus Vaccination in past 2 years**						
No	1.00	Reference	1.00	Reference	1.00	Reference
Yes	0.78	(0.66, 0.92)	0.84	(0.64, 1.10)	0.94	(0.79, 1.11)
**Sex of Child**						
Male	1.00	Reference	1.00	Reference	1.00	Reference
Female	0.88	(0.77, 0.99)	1.18	(0.96, 1.46)	0.89	(0.78, 1.01)
**Gestational Age**						
Very preterm (<32 weeks)	17.44	(14.54, 20.91)	47.53	(28.85, 78.31)	22.57	(15.95, 31.95)
Moderate to late preterm (32-37 weeks)	2.66	(2.25, 3.14)	2.92	(2.16, 3.94)	2.70	(2.21, 3.30)
Term (37-42 weeks)	1.00	Reference	1.00	Reference	1.00	Reference
Post-term (42-45 weeks)	1.42	(1.16, 1.75)	1.13	(0.81, 1.58)	1.25	(1.00, 1.57)
**Size for Gestational Age (within 72 hours)**						
AGA	1.00	Reference	1.00	Reference		
SGA	1.76	(1.42, 2.18)	2.31	(1.78, 3.00)		
LGA	0.63	(0.35, 1.12)	0.09	(0.04, 0.19)		
**Imputed Size for Gestational Age**						
AGA	1.00	Reference			1.00	Reference
SGA	1.43	(1.10, 1.87)			1.66	(1.18, 2.34)
LGA	4.52	(3.49, 5.85)			0.76	(0.52, 1.10)
**Singleton/Twin/Triplet**						
Singleton	1.00	Reference	1.00	Reference	1.00	Reference
Twin or Triplet	8.31	(6.43, 10.74)	3.15	(1.98, 5.04)	3.97	(2.97, 5.30)

For peer review only

STROBE Statement—checklist of items that should be included in reports of observational studies

	Item No	Recommendation	Page No
**Title and abstract**	1	(*a*) Indicate the study's design with a commonly used term in the title or the abstract	1
		(*b*) Provide in the abstract an informative and balanced summary of what was done and what was found	2-3
**Introduction**			
Background/rationale	2	Explain the scientific background and rationale for the investigation being reported	4
Objectives	3	State specific objectives, including any prespecified hypotheses	4
**Methods**			
Study design	4	Present key elements of study design early in the paper	4-5
Setting	5	Describe the setting, locations, and relevant dates, including periods of recruitment, exposure, follow-up, and data collection	4-5
Participants	6	(*a*) *Cohort study*—Give the eligibility criteria, and the sources and methods of selection of participants. Describe methods of follow-up<br>*Case-control study*—Give the eligibility criteria, and the sources and methods of case ascertainment and control selection. Give the rationale for the choice of cases and controls<br>*Cross-sectional study*—Give the eligibility criteria, and the sources and methods of selection of participants	4-5
		(*b*) *Cohort study*—For matched studies, give matching criteria and number of exposed and unexposed<br>*Case-control study*—For matched studies, give matching criteria and the number of controls per case	
Variables	7	Clearly define all outcomes, exposures, predictors, potential confounders, and effect modifiers. Give diagnostic criteria, if applicable	5-7
Data sources/ measurement	8*	For each variable of interest, give sources of data and details of methods of assessment (measurement). Describe comparability of assessment methods if there is more than one group	6-7
Bias	9	Describe any efforts to address potential sources of bias	6-7, 13-14
Study size	10	Explain how the study size was arrived at	5, Figure1
Quantitative variables	11	Explain how quantitative variables were handled in the analyses. If applicable, describe which groupings were chosen and why	5-7
Statistical methods	12	(*a*) Describe all statistical methods, including those used to control for confounding	5-7,13-14
		(*b*) Describe any methods used to examine subgroups and interactions	5-7,13-14
		(*c*) Explain how missing data were addressed	5-7,13-14
		(*d*) *Cohort study*—If applicable, explain how loss to follow-up was addressed<br>*Case-control study*—If applicable, explain how matching of cases and controls was addressed<br>*Cross-sectional study*—If applicable, describe analytical methods taking account of sampling strategy	5-7, Figure1
		(*e*) Describe any sensitivity analyses	14

Continued on next page

For peer review only

**Results**			
Participants	13*	(a) Report numbers of individuals at each stage of study—eg numbers potentially eligible, examined for eligibility, confirmed eligible, included in the study, completing follow-up, and analysed	7-10, Figure1
		(b) Give reasons for non-participation at each stage	Figure1
		(c) Consider use of a flow diagram	Figure1
Descriptive data	14*	(a) Give characteristics of study participants (eg demographic, clinical, social) and information on exposures and potential confounders	8-10
		(b) Indicate number of participants with missing data for each variable of interest	8-10
		(c) *Cohort study*—Summarise follow-up time (eg, average and total amount)	
Outcome data	15*	*Cohort study*—Report numbers of outcome events or summary measures over time	
		Case-control study—Report numbers in each exposure category, or summary measures of exposure	
		Cross-sectional study—Report numbers of outcome events or summary measures	8-9,11-12
Main results	16	(*a*) Give unadjusted estimates and, if applicable, confounder-adjusted estimates and their precision (eg, 95% confidence interval). Make clear which confounders were adjusted for and why they were included	8-13
		(*b*) Report category boundaries when continuous variables were categorized	8-12
		(*c*) If relevant, consider translating estimates of relative risk into absolute risk for a meaningful time period	
Other analyses	17	Report other analyses done—eg analyses of subgroups and interactions, and sensitivity analyses	12-14
**Discussion**			
Key results	18	Summarise key results with reference to study objectives	13-14
Limitations	19	Discuss limitations of the study, taking into account sources of potential bias or imprecision. Discuss both direction and magnitude of any potential bias	13-14
Interpretation	20	Give a cautious overall interpretation of results considering objectives, limitations, multiplicity of analyses, results from similar studies, and other relevant evidence	13-14
Generalisability	21	Discuss the generalisability (external validity) of the study results	14
**Other information**			
Funding	22	Give the source of funding and the role of the funders for the present study and, if applicable, for the original study on which the present article is based	15

*Give information separately for cases and controls in case-control studies and, if applicable, for exposed and unexposed groups in cohort and cross-sectional studies.

Note: An Explanation and Elaboration article discusses each checklist item and gives methodological background and published examples of transparent reporting. The STROBE checklist is best used in conjunction with this article (freely available on the Web sites of PLoS Medicine at http://www.plosmedicine.org/, Annals of Internal Medicine at http://www.annals.org/, and Epidemiology at http://www.epidem.com/). Information on the STROBE Initiative is available at www.strobe-statement.org.

For peer review only

For peer review only - http://bmjopen.bmj.com/site/about/guidelines.xhtml

Risk Factors for Neonatal Mortality; An Observational Cohort Study in Sarlahi District of Rural Southern Nepal

Journal:	*BMJ Open*
Manuscript ID	bmjopen-2022-066931.R1
Article Type:	Original research
Date Submitted by the Author:	28-Mar-2023

Complete List of Authors:	Yan, Tingting; Johns Hopkins University Bloomberg School of Public Health, International Health<br>Mullany, Luke C.; Johns Hopkins University Bloomberg School of Public Health, International Health<br>Subedi, Seema; Johns Hopkins University Bloomberg School of Public Health, International Health<br>Hazel , Elizabeth; Johns Hopkins University Bloomberg School of Public Health<br>Khatry, Subarna; Johns Hopkins University Bloomberg School of Public Health, Department of International Health; Nepal Eye Hospital Complex, Nepal Nutrition Intervention Project - Sarlahi (NNIPS)<br>Mohan, Diwakar; Johns Hopkins University Bloomberg School of Public Health, Department of International Health<br>Zeger, Scott; Johns Hopkins University Bloomberg School of Public Health, Department of Biostatistics<br>Tielsch, James; George Washington University School of Public Health and Health Services, Department of Global Health<br>LeClerq, Steven; Johns Hopkins University Bloomberg School of Public Health, Department of International Health; Nepal Eye Hospital Complex, Nepal Nutrition Intervention Project - Sarlahi (NNIPS)<br>Katz, Joanne; Johns Hopkins University Bloomberg School of Public Health, Department of International Health

**Primary Subject Heading**:	Public health
Secondary Subject Heading:	Global health, Epidemiology, Paediatrics
Keywords:	Epidemiology < TROPICAL MEDICINE, Public health < INFECTIOUS DISEASES, Risk Factors, Factor Analysis, Statistical, Primary Prevention, NEONATOLOGY

I, the Submitting Author has the right to grant and does grant on behalf of all authors of the Work (as defined in the below author licence), an exclusive licence and/or a non-exclusive licence for contributions from authors who are: i) UK Crown employees; ii) where BMJ has agreed a CC-BY licence shall apply, and/or iii) in accordance with the terms applicable for US Federal Government officers or employees acting as part of their official duties; on a worldwide, perpetual, irrevocable, royalty-free basis to BMJ Publishing Group Ltd ("BMJ") its licensees and where the relevant Journal is co-owned by BMJ to the co-owners of the Journal, to publish the Work in this journal and any other BMJ products and to exploit all rights, as set out in our licence.

The Submitting Author accepts and understands that any supply made under these terms is made by BMJ to the Submitting Author unless you are acting as an employee on behalf of your employer or a postgraduate student of an affiliated institution which is paying any applicable article publishing charge ("APC") for Open Access articles. Where the Submitting Author wishes to make the Work available on an Open Access basis (and intends to pay the relevant APC), the terms of reuse of such Open Access shall be governed by a Creative Commons licence – details of these licences and which Creative Commons licence will apply to this Work are set out in our licence referred to above.

Other than as permitted in any relevant BMJ Author's Self Archiving Policies, I confirm this Work has not been accepted for publication elsewhere, is not being considered for publication elsewhere and does not duplicate material already published. I confirm all authors consent to publication of this Work and authorise the granting of this licence.

For peer review only

Risk Factors for Neonatal Mortality; An Observational Cohort Study in Sarlahi District of Rural Southern Nepal

Tingting Yan, MSPH[1], Luke C Mullany, PhD[1], Seema Subedi, MPH[1], Elizabeth Hazel, PhD[1], Subarna K Khatry, MBBS, FRCS[1,2], Diwakar Mohan, MD, DrPH[1], Scott Zeger, PhD[3], James M Tielsch, PhD[4], Steven C LeClerq, MPH[1,2], Joanne Katz, ScD[1#]

1. Department of International Health, Johns Hopkins Bloomberg School of Public Health, 615 N. Wolfe Street, Baltimore, MD, 21205, USA
2. Nepal Nutrition Intervention Project - Sarlahi (NNIPS), Nepal Eye Hospital Complex, PO Box 335, Tripureshwor, Kathmandu, Nepal
3. Department of Biostatistics, Johns Hopkins Bloomberg School of Public Health, 615 N. Wolfe Street, Baltimore, MD, 21205, USA
4. Department of Global Health, Milken Institute School of Public Health, George Washington University, 950 New Hampshire Avenue, Washington, DC, USA

Corresponding author: Joanne Katz, ScD. Email: jkatz1@jhu.edu

Word Count. 3894 words. Abstract 300.

Number of figures and tables. This manuscript included 2 figures and 3 tables (2 in supplemental materials).

For peer review only

ABSTRACT

Objectives To assess the association between maternal characteristics, adverse birth outcomes (small-for-gestational-age (SGA) and/or preterm) and neonatal mortality in rural Nepal.

Design This is a secondary observational analysis to identify risk factors for neonatal mortality, using data from a randomized trial to assess the impact of newborn massage with different oils on neonatal mortality in Sarlahi district, Nepal.

Setting Rural Sarlahi district, Nepal

Participants 40,119 pregnant women enrolled from September 9, 2010, to January 16, 2017

Main outcome The outcome variable is neonatal death. Cox regression was used to estimate adjusted Hazard Ratios (aHR) to assess the association between adverse birth outcomes and neonatal mortality.

Results There were 32,004 live births and 998 neonatal deaths. SGA and/or preterm birth was strongly associated with increased neonatal mortality: SGA and preterm [aHR: 7.09, 95% CI: (4.44, 11.31)], SGA and term/post-term [aHR: 2.12, 95% CI: (1.58, 2.86)], appropriate-for-gestational-age (AGA)/large-for-gestational-age (LGA) and preterm [aHR: 3.23, 95% CI: (2.30, 4.54)]. Neonatal mortality was increased with a history of prior child deaths [aHR: 1.53, 95% CI: (1.24, 1.87)], being a twin or triplet [aHR: 5.64, 95% CI: (4.25, 7.48)], births at health posts/clinics or in hospital [aHR: 1.34, 95% CI: (1.13, 1.58)] and on the way to facilities or outdoors [aHR: 2.26, 95% CI: (1.57, 3.26)]. Risk was lower with increasing maternal height from <145 cm to 145-150 cm [aHR: 0.78, 95% CI: (0.65, 0.94)] to ≥150 cm [aHR: 0.57, 95% CI: (0.47, 0.68)], four or more antenatal care (ANC) visits [aHR: 0.67, 95% CI: (0.53, 0.86)] and education >5 years [aHR: 0.75, 95% CI: (0.62, 0.92)].

Conclusion SGA and/or preterm birth are strongly associated with increased neonatal mortality. To reduce neonatal mortality, interventions that prevent SGA and preterm births by promoting ANC and facility delivery, and care of high-risk infants after birth should be tested.

Trial Registration Number

NCT01177111

Strengths and limitations of the study

- A large number of pregnancies followed with frequent visits in the neonatal period ensures a statistically powered analysis.
- The availability of a large amount of data for each participants enables a wide range of risk factor to be

For peer review only

investigated.

- The date of last menstrual period was obtained early in pregnancy, limiting recall bias, and stillbirths were identified, but both could lead to some misclassification of preterm birth and neonatal death.
- Missing weights due to early newborn deaths or weighing of the baby 72 hours or more after birth were estimated by using multiple imputation, which increased the variability but reduced bias of risk estimates.

For peer review only

INTRODUCTION

Globally, 2.3 million children died in the first 28 days of life in 2021, with the neonatal mortality rate of 18 deaths per 1,000 live births(1). Almost 99% of neonatal deaths occur in low- and middle-income countries (LMICs) (2). Neonatal mortality in Nepal declined from 50 per 1,000 live births in 1996 to 21 in 2022 (2), but neonatal deaths account for a higher percentage of under-5 child deaths since mortality rates among older children have decreased faster than neonatal mortality(2,3). If the trend continues, it would take another 50 years(4) to reach the Sustainable Development Goals target of reducing neonatal mortality to 12 per 1,000 live births by 2030(5).

Although Nepal has continuously reformed its primary health care (PHC) system and expanded the number of health facilities(6), there are still daunting obstacles hindering neonatal health, including lacking access to antenatal care, limited health infrastructure, poor transport and communication, inadequate affordability(7), and significant discrepancy of Nepalese households' access to health services between urban and rural areas(8). Studies have also shown numerous risk factors contributing to neonatal mortality(9–15). Small-for-gestational-age (SGA) and preterm births are associated with neonatal deaths in low-and middle-income countries (LMICs) (10). Other factors include maternal age(11) and education(12), household income(13), previous pregnancy history(14), tetanus vaccination(15), attendance at antenatal care (ANC)(16), and place of delivery(17).

To identify relatively important ones and explore more evidence for interventions to reduce neonatal mortality in specific settings, we used data from the Nepal Oil Massage Study (NOMS), a community-based, cluster-randomized controlled trial in rural southern Nepal to conduct an observational analysis to assess the association between maternal characteristics, adverse birth outcomes (small-for-gestational-age (SGA) and/or preterm) and other maternal and infant characteristics and neonatal mortality.

METHODS

Study Setting

This study was conducted in Sarlahi District of Southern Nepal, a poor rural area of subsistence farming in the low-lying plains of Nepal bordering Bihar, India. In Sarlahi District, neonatal mortality ranges from 30.0-41.9 deaths per 1000 live births, even higher than the average in Nepal(18); 30% of births are <2500 grams and over 92% of births occur at home. Antenatal care in this setting is limited(19).

Study Design

The NOMS study was a cluster-randomized, community-based trial (Clinicaltrials.gov (NCT01177111)). The geographic area was divided into sectors that were randomized to receive promotion of full-body

For peer review only

massage with mustard oil, which is the standard practice, or the intervention group that received promotion of full-body massage with sunflower seed oil. All women of childbearing age in the 34 participating Village Development Committees (VDCs) were eligible for the trial. Women who consented to pregnancy surveillance were visited every 5 weeks and asked whether they had their period since the last visit. If not, they were offered a pregnancy test. If positive, they were enrolled in the trial. Women were followed with monthly visits until delivery. A birth assessment was conducted on the first visit to the newborn baby (the day of birth or as soon after birth as possible), and neonates were followed up on 1, 3, 7, 10, 14, 21 and 28 days of age. Vital status was collected on mothers and infants at each of these time points. A verbal autopsy was conducted on the causes of neonatal death and the date of death was also recorded. For the analysis of risk factors for neonatal mortality, only live born infants and their mothers were included.

Data Collection

Data for the trial were collected from November 2010 through January 2017. All data collectors were employed and trained by the study. This included local village women who collected data from the pregnant women in their communities. Data such as anthropometry were collected by study employees trained and standardized to collect these measurements. For more complex survey data and supervisory staff, local people with prior experience collecting these types of data were employed. At the time women were identified as pregnant and enrolled, date of last menstrual period, maternal age, height, education and reproductive history were recorded. Information about household assets, caste and ethnicity of the family was also collected at the time of enrollment. During pregnancy, baseline and monthly visits in pregnancy recorded receipt of tetanus vaccination, tobacco and alcohol use, and maternal morbidity. In the first visit within 72 hours after birth, late pregnancy morbidity, labor and delivery characteristics and immediate newborn care practices were collected. Neonatal characteristics including sex, weight and the time since birth at which weight was measured. The aim was to measure weight as soon after birth as possible.

Patient and Public Involvement

Our study was an IRB-approved secondary analysis of a pre-existing dataset generated from the NOMS trial. Therefore, it was not appropriate to involve patients or the public in the design, conduct, reporting, or dissemination plans for this research.

Definitions of Variables

Neonatal death was defined as the death of a live born baby in the first 28 days of life and the neonatal mortality rate was defined as the number of neonatal deaths per 1000 live births(20). Low birth weight was defined as a birth weight less than 2500 grams (g)(21). Birth weight was also divided into 4 groups: <1500g

For peer review only

as very low birth weight (VLBW), 1500-2500g as moderately low birth weight (LBW), 2500-4000g (4000g included) as normal birth weight (NBW), >4000g as high birth weight (HBW)(22). For our analysis, it was also categorized into 2 groups: LBW (<2500g) and Normal BW (>=2500g). Gestational age was calculated by taking the number of days between pregnancy outcome and date of last menstrual period obtained by recall at enrollment in the trial(23). Preterm births were babies born alive before 37 completed weeks gestation(24). Gestational age was also categorized into 4 groups: <32, 32-<37, 37-<42, 42-45 weeks, and labeled as very preterm, moderate to late preterm, term, and post-term, respectively(25, 26). For our analysis, it was also categorized into 2 groups: preterm (<37 weeks) and term (37-45 weeks). Small-for-gestational-age (SGA) was defined as newborns with weight below the 10th percentile of newborns using the Intergrowth reference population(27). Appropriate-for-gestational-age (AGA) was defined as weight between the 10th and 90th percentiles of the reference population, and large-for-gestational-age (LGA) was defined as a weight above the 90th percentile of the reference population(27). Parity was defined as the number of prior pregnancies resulting in a live or stillbirth. Gravidity was the number of prior pregnancies, regardless of the outcome of the pregnancy(28).

For peer review only

Data Analysis

The data described above were managed and analyzed using Stata version 16.0(29). Cumulative mortality curves (1-The inverse of Kaplan-Meier (K-M) survival curves) (cumulative mortality) were used to describe the differences in neonatal mortality by SGA and preterm status for specifically four groups: (SGA and preterm, SGA and term/post-term, AGA/LGA and preterm, and AGA/LGA and term/post-term).

Maternal characteristics included demographics, socioeconomic status, lifestyle and reproductive history. Maternal age in pregnancy was categorized as ≤ 18, 18 to 35, and >35 years. Maternal height was categorized as <145, 145 to <150, and ≥ 150 cm. Socioeconomic information included years of education (no schooling as reference, 1 to ≤ 5, and > 5 years), and wealth quintiles based on a standardized score of the total number of household assets including land, animal, transport and mobile phone ownership(30), and caste of the family (Brahmin and Chhetri, Vaishya, Shudra, and Muslim). Reproductive information included parity (prior pregnancy but no parity, no prior pregnancy, 1-4, and ≥5), gravidity (0, and ≥ 1), last pregnancy outcome (no prior pregnancy, at least one live birth, stillbirth, or miscarriage and abortion), a history of child deaths, any prior pregnancy that ended in stillbirth or miscarriage, and if the current pregnancy was a multiple birth (twins or triplets). Maternal exposures in pregnancy included self-reported tobacco and/or alcohol use.

For health care in pregnancy and characteristics of delivery, tetanus vaccination receipt, place of delivery (at home or maternal home, at a health post/clinic or in hospital, or on the way to the facility or outdoors), and

the number of antenatal care (ANC) visits (0, 1, 2 or 3, ≥ 4) were examined.

Neonatal characteristics included birth weight (taken within 72 hours of birth), gestational age, sex, size for gestational age and singleton/twin/triplet in our analysis. Because weights were taken at varying times after birth, and birth weight was missing for some infants who died very soon after birth or were measured more than 72 hours after birth, we multiply imputed birth weights (and hence SGA percentiles) for those with missing weights (601/3957, 15.2% missing) and also imputed weight at time zero for all those with weights and times of weight measurement. This was done using an empirical Bayes regression model of early neonatal weight change by estimating, then recalibrating from the conditional distribution of each child's birth weight given a single measurement at a known later time or imputing given missing weight based on longitudinal daily weights from day of birth through 10 days in a population of infants from the same study area as this trial.(31)

We did not include the intervention as a coviariate in the regression model because the results of the randomized trial are being written up for publication but are not yet citable. However, there was no significant difference in mortality between the intervention and control groups. Hence, not including this variable in the regression should not impact the results presented here.

For estimating hazard ratios we used Cox regression. The outcome was defined as time from birth to death or time the baby was last seen alive. This analysis takes account of loss to follow up and variable ages at death.

In the simple Cox regression models, we calculated the cHR and their 95% CI for each covariate. In multivariable regression, we did not include some variables because of collinearity. For example, we did not include both parity and gravidity. We did not include prior miscarriage or stillbirth because we included prior child deaths. We developed 4 different models to examine the adjusted hazard ratios (aHR) and their 95% CI for risk factors. In model 1, we included maternal age, height, years of education, wealth quintile, caste, parity, prior child deaths, tetanus vaccination, place of delivery, number of ANC visits, sex of the infant, preterm, adverse birth outcomes (SGA and preterm, SGA and term/post-term, AGA/LGA and preterm, and AGA/LGA and term/post-term), and singleton/twin/triplet. In model 2, we included all the variables in model 1, except place of delivery and number of ANC visits. We ran the models with and without these two variables because about 10% of the population was missing these variables and we wanted to see whether excluding these variables produced similar regression results for the predictors of interest to when they were included. For models 3 and 4 (in supplemental materials), we included gestational age and size for gestation separately, to compare with models 1 and 2 respectively. We ran each of these 4 models twice, one time where we used weights and associated SGA/preterm taken within 72 hours of birth, and

For peer review only - http://bmjopen.bmj.com/site/about/guidelines.xhtml

again using the multiply imputed weights and imputed SGA. This allowed us to compare how the imputation impacted the regression coefficients. Since some women had more than one pregnancy included in the analysis, we accounted for correlation within women using robust variance estimation (32).

For multivariable Cox regression, we have assessed the fitness of each of the four models using Stata and found that none indicated a significant deviation from the proportional hazard assumption.

RESULTS

A total of 32,004 live-born babies were included in this analysis. **Figure 1** shows the number of pregnancies identified, enrolled, and their outcomes (not including imputed birthweight). The baseline characteristics, neonatal mortality, and cHR of each potential risk factor is shown in **Table 1**. Most pregnant women were between ages 18-35 years. 15% of women were shorter than 145 cms and 67% had no schooling. 29% had no prior pregnancy and almost no women reported the use of alcohol or tobacco during pregnancy (Table 1). While 42% of women delivered in a facility, 97% of births were vaginal deliveries.

For peer review only

Table 1 Characteristics, Neonatal Mortality and Crude Hazard Ratio of Potential Risk Factors (N=32,004)

Variables	Study Population (N=32,004)			Neonatal Mortality (per 1,000)	Crude Hazard Ratio	95% CI
	Deaths	Total	Distribu-tion %			
**Maternal Age (years) at LMP**						
Mean, 95% CI: 22.46 (22.41, 22.51)						
<=18	218	4,948	15.46	44.06	1.57	(1.34, 1.83)
18-35	748	26,351	82.34	28.39	1.00	Reference
>35	32	703	2.20	45.52	1.62	(1.14, 2.31)
Missing	0	2		0.00		
**Maternal Height (cm)**						
Mean, 95% CI: 150.55 (150.49, 150.61)						
<145	232	4,703	14.72	49.33	1.00	Reference
145-150	332	9,587	30.00	34.64	0.70	(0.59, 0.83)
>=150	429	17,664	55.28	24.29	0.49	(0.41, 0.57)
Missing	5	50		100.00		
**Maternal Education (years)**						
Mean, 95% CI: 2.61 (2.56, 2.65)						
No schooling	748	21,557	67.42	34.70	1.00	Reference
1-5	77	2,721	8.51	28.30	0.81	(0.64, 1.03)
>5	173	7,695	24.07	22.48	0.64	(0.54, 0.76)
Missing	0	31		0.00		
**Wealth Quintile**						
Poorest	262	6,534	20.43	40.10	1.00	Reference
Poorer	223	6,407	20.03	34.81	0.87	(0.72, 1.05)
Middle	206	6,400	20.01	32.19	0.80	(0.66, 0.97)
Richer	154	6,303	19.71	24.43	0.60	(0.49, 0.74)
Richest	152	6,339	19.82	23.98	0.59	(0.48, 0.73)
Missing	1	21		47.62		
**Caste of the Family**						
Brahmin and Chhetri	26	965	3.02	26.94	1.00	Reference
Vaishya	682	23,062	72.13	29.57	1.10	(0.74, 1.63)
Shudra	208	4,947	15.47	42.05	1.57	(1.05, 2.37)
Muslim and others	81	2,999	9.38	27.01	1.01	(0.65, 1.57)
Missing	1	31		32.26		
**Parity**						
1-4	523	20,444	64.22	25.58	1.00	Reference
>=5	62	1,398	4.39	44.35	1.75	(1.33, 2.30)
Prior pregnancy but no parity	35	791	2.48	44.25	1.75	(1.24, 2.47)
No prior pregnancy	369	9,199	28.90	40.11	1.58	(1.38, 1.82)
Missing	9	172		52.33		
**Gravidity**						
>=1	629	22,804	71.26	27.58	1.00	Reference
0 (First pregnancy)	369	9,199	28.74	40.11	1.46	(1.28, 1.67)
Missing	0	1		0.00		
**Last pregnancy outcome**						
No prior pregnancy	369	9,199	28.76	40.11	1.53	(1.34, 1.76)
At least one live birth	535	20,284	63.43	26.38	1.00	Reference
Stillbirth	19	532	1.66	35.71	1.36	(0.86, 2.14)
Miscarriage and abortion	75	1,965	6.14	38.17	1.46	(1.13, 1.89)
Missing	0	24		0.00		
**Prior child deaths**						
No prior pregnancy	369	9,199	29.19	40.11	1.76	(1.52, 2.04)
Prior live births but no child deaths	405	17,597	55.83	23.02	1.00	Reference
Prior live births and child death	149	3,641	11.55	40.92	1.80	(1.48, 2.18)
Prior pregnancy but no live birth	45	1,081	3.43	41.63	1.83	(1.33, 2.53)
Missing	30	486		61.73		
**Any prior still birth**						
No prior pregnancy	369	9,199	28.76	40.11	1.52	(1.32, 1.74)
Prior pregnancy but no still births	571	21,394	66.88	26.69	1.00	Reference
Prior still births	58	1,394	4.36	41.61	1.58	(1.18, 2.10)
Missing	0	17		0.00		
**Any prior miscarriage**						
No prior pregnancy	369	9,199	28.75	40.11	1.54	(1.34, 1.77)
Prior pregnancy but no miscarriage	503	19,142	59.83	26.28	1.00	Reference
Prior miscarriage	126	3,652	11.41	34.50	1.32	(1.08, 1.62)
Missing	0	11		0.00		
**Any prior multiple births**						
No prior pregnancy	369	9,199	28.76	40.11	1.49	(1.30, 1.70)
Prior pregnancy but no multiples	611	22,484	70.30	27.17	1.00	Reference
Prior multiples	18	299	0.93	60.20	2.25	(1.38, 3.68)
Missing	0	22		0.00		
**Tetanus vaccination in the past 2 years**						
No	193	5,066	15.83	38.10	1.00	Reference
Yes	805	26,938	84.17	29.88	0.78	(0.66, 0.92)
Missing	0	0				
**Any tobacco use in pregnancy**						
No	987	31,649	98.89	31.19	1.00	Reference
Yes	11	355	1.11	30.99	0.99	(0.55, 1.80)
Missing	0	0				
**Any alcohol use in pregnancy**						
No	997	31,908	99.70	31.34	1.00	Reference

For peer review only

Yes	1	96	0.30	10.42	0.33	(0.05, 2.37)
Missing	0	0				
**Place of delivery**						
At home or Maiti*	437	15,872	55.46	27.53	1.00	Reference
At health post/clinic or in hospital	405	12,130	42.38	33.39	1.22	(1.06, 1.40)
On the way to facility or outdoors	40	617	2.16	64.83	2.43	(1.70, 3.48)
Missing	116	3,385		34.27		
**Number of Antenatal Care (ANC) visits**						
0	184	5,559	19.46	33.10	1.00	Reference
1	148	4,173	14.61	35.47	1.07	(0.86, 1.34)
2 or 3	366	9,872	34.55	37.07	1.12	(0.93, 1.35)
>=4	183	8,967	31.38	20.41	0.61	(0.50, 0.76)
Missing	117	3,433		34.08		
**Intervention**						
Mustard Oil	520	16,327	51.02	31.85	1.00	Reference
Sunflower Oil	478	15,676	48.98	30.49	0.95	(0.84, 1.09)
Missing	0	1		0		
**Sex of Child**						
Male	548	16,531	51.73	26.63	1.00	Reference
Female	449	15,424	48.27	29.91	0.88	(0.77, 0.99)
Missing	1	49		20.41		
**Birth Weight (within 72 hours)**						
Mean, 95%CI: 2773.19g (2766.95, 2779.44)						
VLBW (<1500g)	51	128	0.46	398.44	69.88	(49.58, 98.50)
LBW (1500-2500g)	199	7,859	28.02	25.32	3.43	(2.76, 4.26)
NBW (2500-4000g)	141	19,354	69.01	7.29	1.00	Reference
HBW (>4000g)	3	706	2.52	4.25	0.57	(0.18, 1.78)
Missing	601	3,957		151.88		
**Imputed Birth Weight**						
Mean, 95%CI: 2774.9g (2770.1, 2779.8)						
VLBW (<1500g)	65	158	0.00	412.98	25.94	(19.38, 34.73)
LBW (1500-2500g)	491	8,298	26.00	59.22	3.23	(2.65, 3.94)
NBW (2500-4000g)	437	23,355	73.17	18.69	1.00	Reference
HBW (>4000g)	4	110	0.00	32.72	1.74	(0.59, 5.14)
Missing	1	83		12.05		
**Birth Weight (within 72 hours)**						
LBW (<2500g)	250	7,987	28.48	31.30	4.32	(3.51, 5.33)
Normal BW (>=2500g)	147	20,060	71.52	7.33	1.00	Reference
Missing	601	3,957		151.88		
**Imputed Birth Weight**						
LBW (<2500g)	556	8,456	26.49	65.77	3.59	(2.99, 4.32)
Normal BW (>=2500g)	440	23,465	73.51	18.76	1.00	Reference
Missing	1	83		12.05		
**Gestational Age (days)**						
Mean, 95%CI: 275.74 (275.49,275.99)						
Very preterm (<32 weeks)	193	662	2.12	291.54	17.44	(14.54, 20.91)
Moderate to late preterm (32-37 weeks)	224	4,244	13.62	52.78	2.66	(2.25, 3.14)
Term (37-42 weeks)	451	22,371	71.78	20.16	1.00	Reference
Post-term (42-45 weeks)	111	3,887	12.47	28.56	1.42	(1.16, 1.75)
Missing	0	3		0.00		
**Gestational Age**						
Preterm (<37 weeks)	417	4,906	15.74	85.00	4.12	(3.60, 4.71)
Term (37-45 weeks)	562	26,258	84.26	21.40	1.00	Reference
Missing	0	3		0.00		
**Size for Gestational Age (within 72 hours)**						
AGA	140	13,172	47.01	10.69	1.00	Reference
SGA	244	13,060	46.61	18.54	1.76	(1.42, 2.18)
LGA	12	1,789	6.38	6.83	0.63	(0.35, 1.12)
Missing	602	3,983		148.36		
**Imputed Size for Gestational Age**						
AGA	373	16,127	50.57	23.15	1.00	Reference
SGA	471	14,275	44.77	32.96	1.43	(1.10, 1.87)
LGA	147	1,486	4.66	98.95	4.52	(3.49, 5.85)
Missing	7	116		60.34		
**SGA/Preterm (within 72 hours)**						
AGA/LGA and Term/Post-term	70	11,381	41.64	6.15	1.00	Reference
SGA and Term/Post-term	185	11,859	43.39	15.60	2.55	(1.93, 3.37)
AGA/LGA and Preterm	81	3,437	12.58	23.57	3.86	(2.76, 5.39)
SGA and Preterm	54	652	2.39	82.82	13.95	(9.67, 20.13)
Missing	608	4,675		130.05		
**Imputed SGA/Preterm**						
AGA/LGA and Term/Post-term	189	13,413	43.42	14.06	1.00	Reference
SGA and Term/Post-term	391	13,607	41.32	28.76	2.06	(1.50, 2.83)
AGA/LGA and Preterm	324	4,175	13.30	77.55	5.73	(4.56, 7.21)
SGA and Preterm	79	669	1.97	118.38	8.82	(5.85, 13.28)
Missing	15	140		60.34		
**Singleton/Twin/Triplet**						
Singleton	892	31,502	98.43	28.32	1.00	Reference
Twin or Triplet	106	502	1.57	211.16	8.31	(6.43, 10.74)
Missing	0	0				

* Maiti is the maternal home, where women may go to deliver, especially in the first pregnancy

For peer review only

Based on the crude hazard ratios, maternal height, education, wealth quintile, caste, parity, gravidity, prior child deaths, and prior adverse pregnancy outcomes, place of delivery, number of ANC visits, multiple births, birthweight, SGA and preterm were all significantly associated with neonatal mortality. Birthweight imputation altered the cHRs for high birthweight (> 4000g) from being a risk factor to being protective and LGA (> 90th percentile) from no association to being a significant risk factor. While being SGA and preterm had a large and significant hazard ratio in the analysis without imputed weight values, this hazard ratio increased significantly when birth weights were imputed.

Variables with high proportions of missing data (around 10%) were ANC visits (10.7%), place of delivery (10.6%), birth weight (12.4%) and weights taken after 72 hours (13.5%). Neonatal mortality was higher among women who delivered at a facility or on their way to a facility compared to home births. Women who attended 4 or more ANC visits had lower neonatal mortality risk. Both before and after imputation, very low birth weight and low birth weight significantly increased neonatal mortality. Preterm or post-term resulted in higher neonatal mortality. SGA and LGA babies were at higher risk compared to AGA ones. Alcohol and tobacco use was not included in multivariate models, given the low prevalence of their use in pregnancy.

Multivariable regression results are shown in **Table 2** and **Table 3**. We calculated cHRs for every categories for individual variables. If 95% CIs of cHRs only cover 1.00, we could then consider not to include the variable in our models. These models did not include gravidity, last pregnancy outcome, any prior pregnancy ending in stillbirth, miscarriage and multiples, and tobacco and alcohol use. Model 1 included place of delivery and number of ANC visits, while model 2 did not.

The total number of live births in the analysis was 26,626 with original data (weights taken within 72 hours), and 27,819 with imputed birthweight and SGA data in model 1 (**Table 2**). Place of delivery had a significant impact on neonatal mortality: the risks were higher both at health posts/clinics or in hospital [aHR: 1.34, 95% CI: (1.13, 1.58)] and on the way to the facility or outdoors [aHR: 2.26, 95% CI: (1.57, 3.26)]; the number of ANC visits was statistically significant for four or more visits [aHR: 0.67, 95% CI: (0.53, 0.86)].

In model 2 (**Table 3**), the number of participants included was 26,680 with weights taken within 72 hours, and 31,116 after birthweight imputation. The difference was due to more than 10% missing values for place of delivery and number of ANC visits, which were included in model 1, but not in model 2. Results for models 1 and 2 are quite similar, indicating that exclusion of place of delivery and ANC did not change the associations of the other risk factors with mortality.

For peer review only - http://bmjopen.bmj.com/site/about/guidelines.xhtml

Table 2 Multivariate Cox Regression Model 1

Variables	Crude Hazard Ratio	95% CI	**Model 1:** Maternal Age, Mother's Height, Mother's Years of Education, Wealth Quintile, Caste, Parity, Prior Child Deaths, Tetanus Vaccination, Place of Delivery, Number of ANC Visits, Sex of Child, SGA/Preterm, Imputed SGA/Preterm, Singleton/Twin+ Triplet			
			**Adjusted Hazard Ratio (N=26,626)**	**95% CI**	**Adjusted HR (Using imputed SGA) (N=27,819)**	**95% CI**
**Maternal Age (years) at LMP**						
Mean, 95% CI: 22.46 (22.41, 22.51)						
<=18	1.57	(1.34, 1.83)	1.39	(1.04, 1.86)	1.22	(1.01, 1.50)
18-35	1.00	Reference	1.00	Reference	1.00	Reference
>35	1.62	(1.14, 2.31)	1.28	(0.60, 2.77)	1.36	(0.84, 2.21)
**Maternal Height (cm)**						
Mean, 95% CI: 150.55 (150.49, 150.61)						
<145	1.00	Reference	1.00	Reference	1.00	Reference
145-150	0.70	(0.59, 0.83)	0.64	(0.48, 0.86)	0.75	(0.62, 0.92)
150	0.49	(0.41, 0.57)	0.57	(0.43, 0.75)	0.55	(0.46, 0.67)
**Maternal Education (years)**						
Mean, 95% CI: 2.61 (2.56, 2.65)						
No schooling	1.00	Reference	1.00	Reference	1.00	Reference
1-5	0.81	(0.64, 1.03)	1.01	(0.69, 1.49)	0.92	(0.71,1.20)
>5	0.64	(0.54, 0.76)	0.79	(0.57, 1.09)	0.78	(0.62, 0.97)
**Wealth Quintile**						
Poorest	1.00	Reference	1.00	Reference	1.00	Reference
Poorer	0.87	(0.72, 1.05)	0.85	(0.61, 1.17)	0.92	(0.74, 1.13)
Middle	0.80	(0.66, 0.97)	0.96	(0.69, 1.33)	0.95	(0.76, 1.19)
Richer	0.60	(0.49, 0.74)	0.73	(0.50, 1.06)	0.82	(0.64, 1.05)
Richest	0.59	(0.48, 0.73)	0.89	(0.60, 1.32)	0.91	(0.70, 1.18)
**Caste of the Family**						
Brahmin and Chhetri	1.00	Reference	1.00	Reference	1.00	Reference
Vaishya	1.10	(0.74, 1.63)	1.29	(0.57, 2.91)	0.86	(0.55, 1.34)
Shudra	1.57	(1.05, 2.37)	1.61	(0.69, 3.76)	1.01	(0.63, 1.64)
Muslim and others	1.01	(0.65, 1.57)	1.04	(0.43, 2.52)	0.69	(0.41, 1.14)
**Parity**						
1-4	1.00	Reference	1.00	Reference	1.00	Reference
>=5	1.75	(1.33, 2.30)	1.20	(0.66, 2.19)	1.04	(0.70, 1.55)
Prior pregnancy but no parity	1.75	(1.24, 2.47)	1.99	(0.43, 9.11)	1.63	(0.69, 3.84)
No prior pregnancy	1.58	(1.38, 1.82)	1.43	(1.06, 1.92)	1.73	(1.42, 2.12)
**Prior child deaths**						
No prior pregnancy	1.76	(1.52, 2.04)	Omitted		Omitted	
Prior live births but no child deaths	1.00	Reference	1.00	Reference	1.00	Reference
Prior live births and child death	1.80	(1.48, 2.18)	1.11	(0.79, 1.55)	1.56	(1.26, 1.95)
Prior pregnancy but no live birth	1.83	(1.33, 2.53)	0.71	(0.17, 2.93)	1.28	(0.57, 2.88)
**Tetanus vaccination in the past 2 years**						
No	1.00	Reference	1.00	Reference	1.00	Reference
Yes	0.78	(0.66, 0.92)	0.84	(0.63, 1.11)	0.74	(0.62, 0.89)
**Place of delivery**						
At home or Maiti*	1.00	Reference	1.00	Reference	1.00	Reference
At health post/clinic or in hospital	1.22	(1.06, 1.40)	0.94	(0.73, 1.21)	1.34	(1.13, 1.58)
On the way to facility or outdoors	2.43	(1.70, 3.48)	1.42	(0.72, 2.81)	2.26	(1.57, 3.26)
**Number of Antenatal Care (ANC)**						
0	1.00	Reference	1.00	Reference	1.00	Reference
1	1.07	(0.86, 1.34)	1.03	(0.73, 1.47)	1.07	(0.85, 1.34)
2 or 3	1.12	(0.93, 1.35)	1.06	(0.79, 1.42)	1.06	(0.87, 1.29)
>=4	0.61	(0.50, 0.76)	0.77	(0.54, 1.11)	0.67	(0.53, 0.86)
**Sex of Child**						
Male	1.00	Reference	1.00	Reference	1.00	Reference
Female	0.88	(0.77, 0.99)	1.16	(0.94, 1.43)	0.92	(0.80, 1.06)
**SGA/Preterm (within 72 hours)**						
AGA/LGA and Term/Post-term	1.00	Reference	1.00	Reference		
SGA and Term/Post-term	2.55	(1.93, 3.37)	2.11	(1.56, 2.83)		
AGA/LGA and Preterm	3.86	(2.76, 5.39)	3.11	(2.22, 4.35)		
SGA and Preterm	13.95	(9.67, 20.13)	6.90	(4.33, 10.97)		
**Imputed SGA/Preterm**						
AGA/LGA and Term/Post-term	1.00	Reference			1.00	Reference
SGA and Term/Post-term	2.06	(1.50, 2.83)			1.67	(1.10, 2.54)
AGA/LGA and Preterm	5.73	(4.56, 7.21)			4.26	(3.20, 5.66)
SGA and Preterm	8.82	(5.85, 13.28)			3.92	(2.18, 7.07)
**Singleton/Twin/Triplet**						
Singleton	1.00	Reference	1.00	Reference	1.00	Reference
Twin or Triplet	8.31	(6.43, 10.74)	4.89	(2.91, 8.21)	5.43	(4.01, 7.37)

* Maiti is the maternal home, where women may go to deliver, especially in the first pregnancy

For peer review only

For peer review only - http://bmjopen.bmj.com/site/about/guidelines.xhtml

Table 3 Multivariate Cox Regression Model 2

Variables	Crude Hazard Ratio	95% CI	**Model 2**: Maternal Age, Mother's Height, Mother's Years of Education, Wealth Quintile, Caste, Parity, Prior Child Death, Tetanus Vaccination, Sex of Child, SGA/Preterm, Imputed SGA/Preterm, Singleton/Twin/Triplet			
			Adjusted Hazard Ratio (N=26,680)	95% CI	Adjusted HR (Using imputed SGA) (N=31,116)	95% CI
**Maternal Age (years) at LMP**						
Mean, 95% CI: 22.46 (22.41, 22.51)						
<=18	1.57	(1.34, 1.83)	1.41	(1.05, 1.88)	1.15	(0.95, 1.38)
18-35	1.00	Reference	1.00	Reference	1.00	Reference
>35	1.62	(1.14, 2.31)	1.26	(0.58, 2.71)	1.46	(0.93, 2.28)
**Maternal Height (cm)**						
Mean, 95% CI: 150.55 (150.49, 150.61)						
<145	1.00	Reference	1.00	Reference	1.00	Reference
145-150	0.70	(0.59, 0.83)	0.64	(0.48, 0.85)	0.78	(0.65, 0.94)
>=150	0.49	(0.41, 0.57)	0.57	(0.43, 0.75)	0.57	(0.47, 0.68)
**Maternal Education (years)**						
Mean, 95% CI: 2.61 (2.56, 2.65)						
No schooling	1.00	Reference	1.00	Reference	1.00	Reference
1-5	0.81	(0.64, 1.03)	0.98	(0.67, 1.44)	0.89	(0.69, 1.14)
>5	0.64	(0.54, 0.76)	0.75	(0.55, 1.04)	0.75	(0.62, 0.92)
**Wealth Quintile**						
Poorest	1.00	Reference	1.00	Reference	1.00	Reference
Poorer	0.87	(0.72, 1.05)	0.84	(0.61, 1.17)	0.96	(0.79, 1.18)
Middle	0.80	(0.66, 0.97)	0.94	(0.68, 1.30)	0.94	(0.76, 1.16)
Richer	0.60	(0.49, 0.74)	0.71	(0.49, 1.03)	0.82	(0.66, 1.04)
Richest	0.59	(0.48, 0.73)	0.87	(0.59, 1.29)	0.92	(0.72, 1.17)
**Caste of the Family**						
Brahmin and Chhetri	1.00	Reference	1.00	Reference	1.00	Reference
Vaishya	1.10	(0.74, 1.63)	1.34	(0.59, 3.03)	0.86	(0.57, 1.31)
Shudra	1.57	(1.05, 2.37)	1.68	(0.72, 3.93)	1.00	(0.64, 1.57)
Muslim and others	1.01	(0.65, 1.57)	1.08	(0.44, 2.62)	0.67	(0.41, 1.07)
**Parity**						
1-4	1.00	Reference	1.00	Reference	1.00	Reference
>=5	1.75	(1.33, 2.30)	1.21	(0.67, 2.21)	1.05	(0.72, 1.52)
Prior pregnancy but no parity	1.75	(1.24, 2.47)	2.01	(0.44, 9.24)	1.52	(0.71, 3.27)
No prior pregnancy	1.58	(1.38, 1.82)	1.39	(1.05, 1.85)	1.79	(1.50, 2.15)
**Prior child deaths**						
No prior pregnancy	1.76	(1.52, 2.04)	Omitted		Omitted	
Prior live births but no child death	1.00	Reference	1.00	Reference	1.00	Reference
Prior pregnancy but no live births	1.80	(1.48, 2.18)	0.67	(0.16, 2.77)	1.32	(0.65, 2.67)
Prior live births and child death	1.83	(1.33, 2.53)	1.11	(0.79, 1.56)	1.53	(1.24, 1.87)
**Tetanus Vaccination in the past 2 years**						
No	1.00	Reference	1.00	Reference	1.00	Reference
Yes	0.78	(0.66, 0.92)	0.84	(0.63, 1.10)	0.88	(0.74, 1.04)
**Sex of Child**						
Male	1.00	Reference	1.00	Reference	1.00	Reference
Female	0.88	(0.77, 0.99)	1.17	(0.95, 1.45)	0.89	(0.78, 1.01)
**SGA/Preterm (within 72 hours)**						
AGA/LGA and Term/Post-term	1.00	Reference	1.00	Reference		
SGA and Term/Post-term	2.55	(1.93, 3.37)	2.12	(1.58, 2.86)		
AGA/LGA and Preterm	3.86	(2.76, 5.39)	3.23	(2.30, 4.54)		
SGA and Preterm	13.95	(9.67, 20.13)	7.09	(4.44 11.31)		
**Imputed SGA/Preterm**						
AGA/LGA and Term/Post-term	1.00	Reference			1.00	Reference
SGA and Term/Post-term	2.06	(1.50, 2.83)			1.68	(1.20, 2.35)
AGA/LGA and Preterm	5.73	(4.56, 7.21)			4.50	(3.51, 5.77)
SGA and Preterm	8.82	(5.85, 13.28)			4.11	(3.52, 6.69)
**Singleton/Twin/Triplet**						
Singleton	1.00	Reference	1.00	Reference	1.00	Reference
Twin or Triplet	8.31	(6.43, 10.74)	4.76	(2.84, 8.00)	5.64	(4.25, 7.48)

For peer review only

For peer review only - http://bmjopen.bmj.com/site/about/guidelines.xhtml

After imputation and adjustment, variables such as maternal age, wealth quintile, and caste of the family were not significantly associated with neonatal mortality. Increasing maternal height from <145 cm to 145-150 cm [aHR: 0.78, 95% CI: (0.65, 0.94)] to ≥150 cm [aHR: 0.57, 95% CI: (0.47, 0.68)] and education to >5 years [aHR: 0.75, 95% CI: (0.62, 0.92)] remained protective factors. Parity was not significantly associated with neonatal mortality. Prior live births and child death [aHR: 1.53, 95% CI: (1.24, 1.87)] were associated with higher risk. Tetanus vaccination did not have a significant impact on neonatal mortality. Being a twin or triplet [aHR:5.64, 95% CI: (4.25, 7.48)] was also significantly associated with neonatal mortality. The findings were similar in models 1 and 2.

Any SGA and/or preterm was strongly associated with an increased likelihood of neonatal mortality (**Figure 2**): SGA and preterm [aHR: 7.09, 95% CI: (4.44, 11.31)], SGA and term/post-term [aHR: 2.12, 95% CI: (1.58, 2.86)], AGA/LGA and preterm [aHR: 3.23, 95% CI: (2.30, 4.54)]. Imputation of birthweights increased the association between AGA/LGA and preterm and mortality but not the other categories of adverse birth outcomes.

We also created two other multivariate regression models, model S1 and S2 as shown in **Table S1** and **Table S2** in the appendices. These included gestational age and size for gestational age as individual variables.

DISCUSSION

Nepal was one of the few countries to meet the Millennium Development Goal 4 for child mortality but neonatal mortality rates are still high and need to be reduced by twofold to meet the sustainable development goals of 12 per 1000 live births(33,34). This study identified maternal characteristics (maternal height, education, and prior child death), neonatal characteristics (SGA, preterm, and twin or triplet), delivery and health care (place of delivery and number of ANC visits) as significant risk factors for neonatal mortality in Salahi district, rural southern Nepal.

Compared with previous existing studies, maternal age(11,35,36), maternal height(37), maternal education(12,13), wealth(13,35), low birth weight(36,38,39), SGA and preterm(36,40–42), twins(13,40,43) and triplets(43) were all strongly associated with neonatal deaths, which correspond to our findings. Previous studies suggest that parity was not considered a predictor of neonatal mortality(44), which was also supported by our analysis. Health care services, specifically the number of ANC(10,16) visits and place of delivery(17) also have an impact on neonatal deaths. Nevertheless, in our study, neonatal mortality was higher among those delivering in a facility, outdoors or on the way to a facility than at home. This is likely due to some women in this population starting their delivery at home, and only going to a facility if

For peer review only - http://bmjopen.bmj.com/site/about/guidelines.xhtml

something goes wrong during labor and delivery, or being referred to the facility because they were high risk. As the proportion of routine deliveries at facilities increases, this association may reverse.

A previous systematic review indicated that neonatal mortality is reduced by vaccination of women of childbearing age with tetanus toxoid in LMICs(15), while our study found no association with mortality both before and after adjustment. Neonatal tetanus is an unlikely cause of neonatal deaths due to the high rate of tetanus toxoid vaccination among women of childbearing age in Nepal. A recent programmatic focus on vaccinating adolescent girls with tetanus toxoid may also explain this lack of association. Tetanus toxoid vaccine receipt during pregnancy (as collected in this study) is often used as a proxy for ANC utilization in LMICs, but we included the number of ANC visits in the regressions and found it to be associated with neonatal mortality. ANC visits are likely a more proximate risk factor for mortality than tetanus toxoid receipt.

Strengths of this study included a large cohort of pregnancies followed through 28 days postpartum with up to eight visits in the neonatal period. The frequent visits reduced loss to follow up and improved estimates of age at death. While gestational age was not measured using gold standard ultrasound, the date of last menstrual period was obtained early in the pregnancy, limiting recall bias. However, the reliance on date of last menstrual period could have led to some misclassification of preterm births. Another strength of this study was that stillbirths were identified using maternal recall of whether the baby moved, cried, or breathed after birth. If the infant did none of these, they were classified as a stillbirth. However, there could be some misclassification of stillbirths as live births and vice versa. Another limitation is that we did not include maternal morbidity in pregnancy in this analysis. Maternal morbidity may have impacted neonatal mortality but would likely have worked through adverse pregnancy outcomes such as preterm birth and SGA, which were the primary focus of risk factors for mortality in this analysis. Some other limitations include many missing weights due to early newborn death or weighing of the baby 72 hours or more after birth. Most of the missing values for birth weight were estimated by using multiple imputation, which increased the uncertainty of this analysis. The imputation of birth weights also had a qualitative impact on the regression coefficients, in particular for the AGA/LGA and preterm category, which went from an adjusted hazard ratio of 3.13 to one of 5.46. This may be due to the fact that preterm infants who died soon after birth were likely to be missing weights. The neonatal mortality rate among preterm births was 33.02 per 1,000 before imputation and 83.19 per 1,000 after imputation. Similarly, the neonatal mortality rate among SGA before imputation was 19.10 per 1,000 and 32.92 per 1,000 after imputation.

In addition to missing birth weights, about 10% of values were missing for place of delivery and number of ANC visits. We also conducted sensitivity analyses including and excluding these covariates and found that

For peer review only - http://bmjopen.bmj.com/site/about/guidelines.xhtml

the other regression coefficients were not altered significantly by their being included.

Our study found that maternal age, wealth quintile, caste of the family, parity, and tetanus vaccination were not significantly associated with neonatal mortality after adjustment and imputation. Although these findings conflict with some of the existing literature, it is possible that the inclusion of more proximate risk factors such as gestational age and size for gestational age lessened the importance of these other more distal risk factors.

CONCLUSION

Maternal height, maternal education, prior child deaths, SGA, preterm, and twins or triplets, place of delivery and number of ANC visits were significantly associated with neonatal mortality risk. Low birthweight babies who are preterm and SGA, preterm and LGA or SGA and term have different mortality risks and these adverse outcomes likely have different etiologies. These findings could help refine interventions to reduce these adverse birth outcomes. Interventions that focus on preventing SGA and preterm births, improving education for women, promoting quality antenatal health care and facility delivery, and care of vulnerable infants after birth should continue to be emphasized and prioritized.

Contributorship

JK conceived the idea for this paper. All authors contributed to the planning of the study. JK, TY, LM, SS, EH, DM, JT and SZ had discussions and decided the study design, study methods, data analysis, and paper writing. SK and SL mainly contributed to the dataset acquisition. EH was responsible for dataset management, primarily multiple imputation. SS and LM both contributed a lot to help Stata code writing. TY conducted the analysis, including characteristics description, survival analysis and Cox regression and coordinated the whole research process. SZ, JT and DM were instrumental in addressing major technical problems related to statistical methods and models construction. All authors contributed to the interpretation of the findings. TY and JK jointly wrote the first draft and revised the manuscript. All authors critically revised the paper for intellectual content and approved the final version of the manuscript.

Funding

This work was supported by the National Institute for Child Health and Human Development (R01HD092411 and R01HD060712) and the Bill & Melinda Gates Foundation (OPP1084399). The funders did not have a role in the design of the study, the data collection, nor the analysis, interpretation and writing of the manuscript.

Disclaimers

For peer review only

The views expressed in the submitted article are the authors' own and not an official position of the institution or funder.

Competing Interests

Each author has filled ICMJE form for competing interests disclosure. The authors have no conflicts of interest to declare.

Ethics Approval

This analysis of secondary data was considered exempt by the Johns Hopkins Bloomberg School of Public Health institutional review board (IRB) (FWA00000287). The original NOMS study received IRB approval the Johns Hopkins Bloomberg School of Public Health and Tribhuvan University Institute of Medicine in Nepal.

Data Sharing

No additional data are available.

For peer review only

REFERENCES

1. United Nations International Children's Emergency Fund, Unicef. (2023) Neonatal mortality. Retrieved from: https://data.unicef.org/topic/child-survival/neonatal-mortality/#:~:text=Globally%2C%202.3%20million%20children%20died,6%2C400%20neonatal%20deaths%20every%20day.
2. Ministry of Health and Population, Nepal; New ERA; and ICF. 2022. Nepal Demographic and Health Survey 2022: Key Indicators Report. Kathmandu, Nepal: Ministry of Health and Population, Nepal.
3. Paudel D, Thapa A, Shedain PR, Paudel B. Trends and determinants of neonatal mortality in Nepal: further analysis of the Nepal Demographic and Health Surveys, 2001-2011. DHS Further Analysis Report. 2013.
4. Kc A, Jha AK, Shrestha MP, Zhou H, Gurung A, Thapa J, Budhathoki SS. Trends for neonatal deaths in Nepal (2001–2016) to project progress towards the SDG target in 2030, and risk factor analyses to focus action. Maternal and child health journal. 2020 Feb;24:5-14.
5. GoN/NPC. National Planning Commission, 2015: Sustainable Development Goals, 2016-2030, National (Preliminary) Report. Government of Nepal, National Planning Commission, Kathmandu, Nepal. 2015. 39–52 p.
6. Public Health Update (2020). Number of Public Health Facilities in Nepal. Available from: https://publichealthupdate.com/number-of-health-facilities-in-nepal/
7. Joshi, R., Sharma, S., & van Teijlingen, E. (2013). Improving neonatal health in Nepal: major challenges to achieving millennium development goal 4. Health Science Journal, 7(3), 247-257.
8. Mishra, S. R., Khanal, P., Karki, D. K., Kallestrup, P., & Enemark, U. (2015). National health insurance policy in Nepal: challenges for implementation. Global health action, 8(1), 28763.
9. Kamal SMM, Ashrafuzzaman M, Narreen SA. Risk factors of neonatal mortality in Bangladesh. Journal of Nepal Paediatric Society. 2012;32(1).

10. Batieha AM, Khader YS, Berdzuli N, Chua-Oon C, Badran EF, Al-sheyab NA, et al. Level, causes and risk factors of neonatal mortality, in jordan: results of a national prospective study. Maternal and Child Health Journal. 2016;20(5).
11. Sharma V, Katz J, Mullany LC, Khatry SK, LeClerq SC, Shrestha SR, et al. Young maternal age and the risk of neonatal mortality in rural Nepal. Archives of Pediatrics and Adolescent Medicine. 2008;162(9).
12. Kamal SMM. Maternal education as a determinant of neonatal mortality in Bangladesh. Journal of Health Management. 2012;14(3).
13. McKinnon B, Harper S, Kaufman JS, Bergevin Y. Socioeconomic inequality in neonatal mortality in countries of low and middle income: A multicountry analysis. The Lancet Global Health. 2014;2(3).
14. Katz J, West KP, Khatry SK, Christian P, LeClerq SC, Pradhan EK, et al. Risk factors for early infant mortality in Sarlahi district, Nepal. Bulletin of the World Health Organization. 2003;81(10).
15. Khan AA, Zahidie A, Rabbani F. Interventions to reduce neonatal mortality from neonatal tetanus in low- and middle-income countries - A systematic review. BMC Public Health. 2013;13(1).
16. Arunda M, Emmelin A, Asamoah BO. Effectiveness of antenatal care services in reducing neonatal mortality in Kenya: Analysis of national survey data. Global Health Action. 2017;10(1).
17. Ajaari J. Impact of Place of Delivery On Neonatal Mortality In Rural Tanzania. Value in Health. 2013;16(3).
18. Subedi, S., Katz, J., Erchick, D. J., Verhulst, A., Khatry, S. K., Mullany, L. C., ... & Guillot, M. (2022). Does higher early neonatal mortality in boys reverse over the neonatal period? A pooled analysis from three trials of Nepal. BMJ open, 12(5), e056112.
19. Saya, A. R., Katz, J., Khatry, S. K., Tielsch, J. M., LeClerq, S. C., & Mullany, L. C. (2022). Causes of neonatal mortality using verbal autopsies in rural Southern Nepal, 2010 – 2017. PLOS Global Public Health, 2(9), e0001072.
20. World Health Organization. (2022). Indicator Metadata Registry List/Neonatal mortality rate (0 to 27 days) per 1000 live births) (SDG 3.2.2). Retrieved from: https://www.who.int/data/gho/indicator-metadata-registry/imr-details/67
21. World Health Organization. (2022). Low birth weight. Nutrition and nutrition-related health and development data. Retrieved from: https://www.who.int/data/nutrition/nlis/info/low-birth-weight
22. Gill, S. V., May-Benson, T. A., Teasdale, A., & Munsell, E. G. (2013). Birth and developmental correlates of birth weight in a sample of children with potential sensory processing disorder. BMC pediatrics, 13, 1-8.
23. Andersen, H. F., Johnson Jr, T. R., Flora Jr, J. D., & Barclay, M. L. (1981). Gestational age assessment: II. Prediction from combined clinical observations. American journal of obstetrics and gynecology, 140(7), 770-774.
24. World Health Organization. (2022). Preterm birth. Retrieved from: https://www.who.int/news-room/fact-sheets/detail/preterm-birth
25. Spong, C. Y. (2013). Defining "term" pregnancy: recommendations from the Defining "Term" Pregnancy Workgroup. Jama, 309(23), 2445-2446.
26. Galal, M., Symonds, I., Murray, H., Petraglia, F., & Smith, R. (2012). Postterm pregnancy. Facts, views & vision in ObGyn, 4(3), 175.
27. Villar J, Ismail LC, Victora CG, Ohuma EO, Bertino E, Altman DG, et al. International standards for newborn weight, length, and head circumference by gestational age and sex: The Newborn Cross-Sectional Study of the INTERGROWTH-21st Project. The Lancet. 2014;384(9946).
28. Tidy, C., & Payne, J. (2019). Gravidity and Parity Definitions (Implications in Risk Assessment).
29. StataCorp. Stata Statistical Software: Release 16. College Station, TX: StataCorp LLC. StataCorp LLC. 2019.
30. Siegel EH, Stoltzfus RJ, Khatry SK, LeClerq SC, Katz J, Tielsch JM. Epidemiology of anemia among 4- to 17-month-old children living in south central Nepal. European Journal of Clinical Nutrition. 2006;60(2).
31. Hazel E, Mullany LC, Zeger SL, Mohan D, Subedi S, Tielsch JM, Khatry SK, Katz J. (2022).

For peer review only - http://bmjopen.bmj.com/site/about/guidelines.xhtml

Development of an imputation model to recalibrate birthweights measured in the early neonatal period to time at delivery and assessment of its impact on size-for-gestational age and low birthweight prevalence estimates: a secondary analysis of a pregnancy cohort in rural Nepal. BMJ open, 12(7), e060105.

32. Lin, Danyu Y., and Lee-Jen Wei. The robust inference for the Cox proportional hazards model. Journal of the American statistical Association 84.408 (1989): 1074-1078.
33. Boerma T, Requejo J, Victora CG, Amouzou A, George A, Agyepong I, et al. Countdown to 2030: tracking progress towards universal coverage for reproductive, maternal, newborn, and child health. Vol. 391, The Lancet. 2018.
34. Victora CG, Requejo JH, Barros AJD, Berman P, Bhutta Z, Boerma T, et al. Countdown to 2015: A decade of tracking progress for maternal, newborn, and child survival. Vol. 387, The Lancet. 2016.
35. Neupane S, Doku DT. Neonatal mortality in Nepal: A multilevel analysis of a nationally representative. Journal of Epidemiology and Global Health. 2014;4(3).
36. de Jesus LC, Pappas A, Shankaran S, Kendrick D, Das A, Higgins RD, et al. Risk factors for post-neonatal intensive care unit discharge mortality among extremely low birth weight infants. Journal of Pediatrics. 2012;161(1).
37. Khatun W, Alam A, Rasheed S, Huda TM, Dibley MJ. Exploring the intergenerational effects of undernutrition: Association of maternal height with neonatal, infant and under-five mortality in Bangladesh. BMJ Global Health. 2018;3(6).
38. Pulver LS, Guest-Warnick G, Stoddard GJ, Byington CL, Young PC. Weight for gestational age affects the mortality of late preterm infants. Pediatrics. 2009;123(6).
39. Yasmin S, Osrin D, Paul E, Costello A. Neonatal mortality of low-birth-weight infants in Bangladesh. Bulletin of the World Health Organization. 2001;79(7).
40. Blickstein I, Keith LG. Neonatal mortality rates among growth-discordant twins, classified according to the birth weight of the smaller twin. American Journal of Obstetrics and Gynecology. 2004;190(1).
41. Clausson B, Cnattingius S, Axelsson O. Preterm and term births of small for gestational age infants: A population-based study of risk factors among nulliparous women. BJOG: An International Journal of Obstetrics and Gynaecology. 1998;105(9).
42. Kozuki N, Lee AC, Silveira MF, Sania A, Vogel JP, Adair L, et al. The associations of parity and maternal age with small-for-gestational-age, preterm, and neonatal and infant mortality: A meta-analysis. Vol. 13, BMC Public Health. 2013.
43. Garite TJ, Clark RH, Elliott JP, Thorp JA. Twins and triplets: The effect of plurality and growth on neonatal outcome compared with singleton infants. American Journal of Obstetrics and Gynecology. 2004;191(3).
44. Wulandari RD, Laksono AD. Is parity a predictor of neonatal death in Indonesia Analysis of the 2017 Indonesia demographic and health survey. Indian Journal of Forensic Medicine and Toxicology. 2020;14(3).

Figure 1 Flow Diagram for Participants in NOMS Study

Figure 2 Cumulative Neonatal Mortality Curves by SGA/Preterm Excluding Imputed Data

For peer review only

Figure 1 Flow Diagram for Participants in NOMS Study

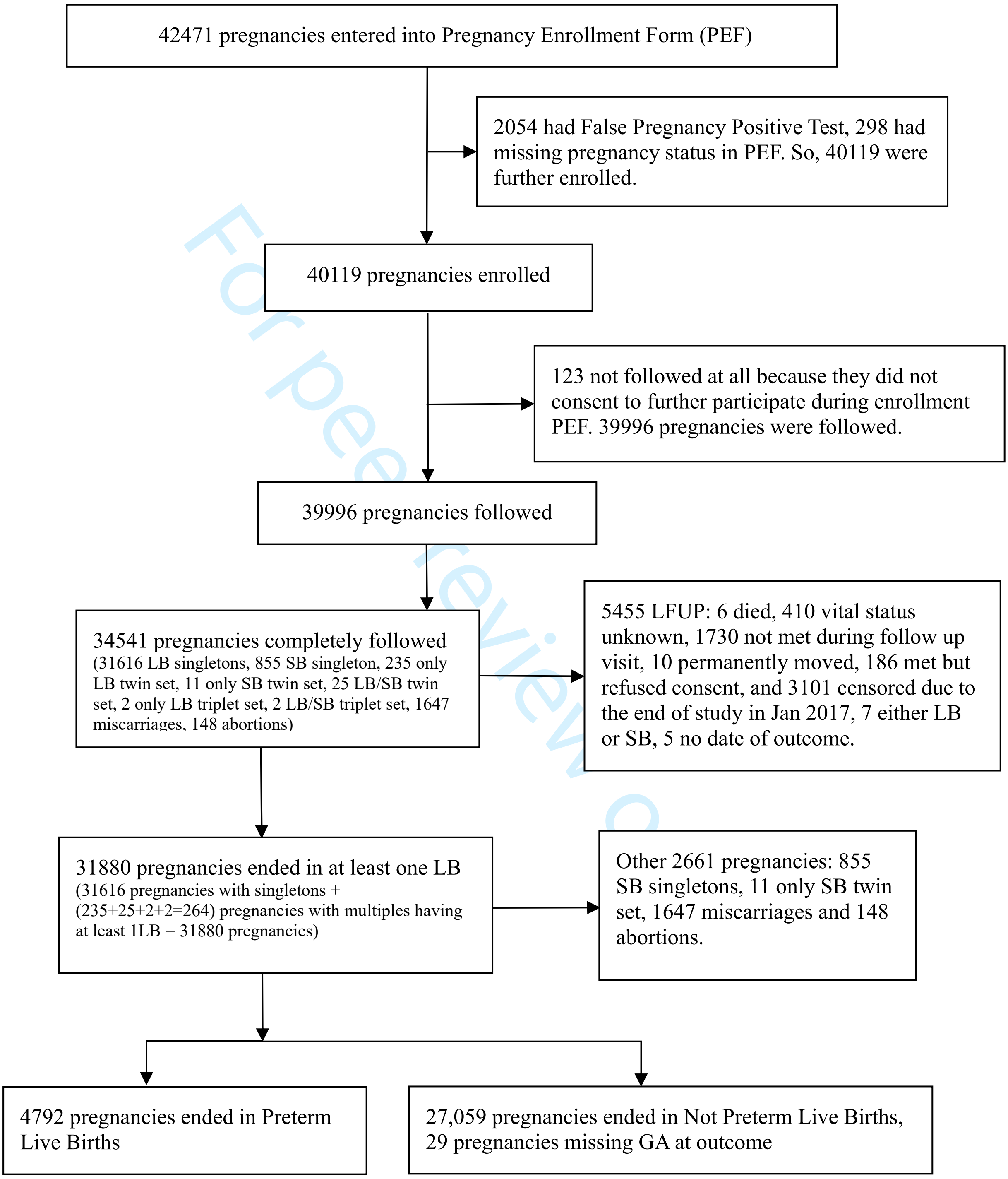

**Data collection was halted for 6 weeks in December 2016.*

For peer review only

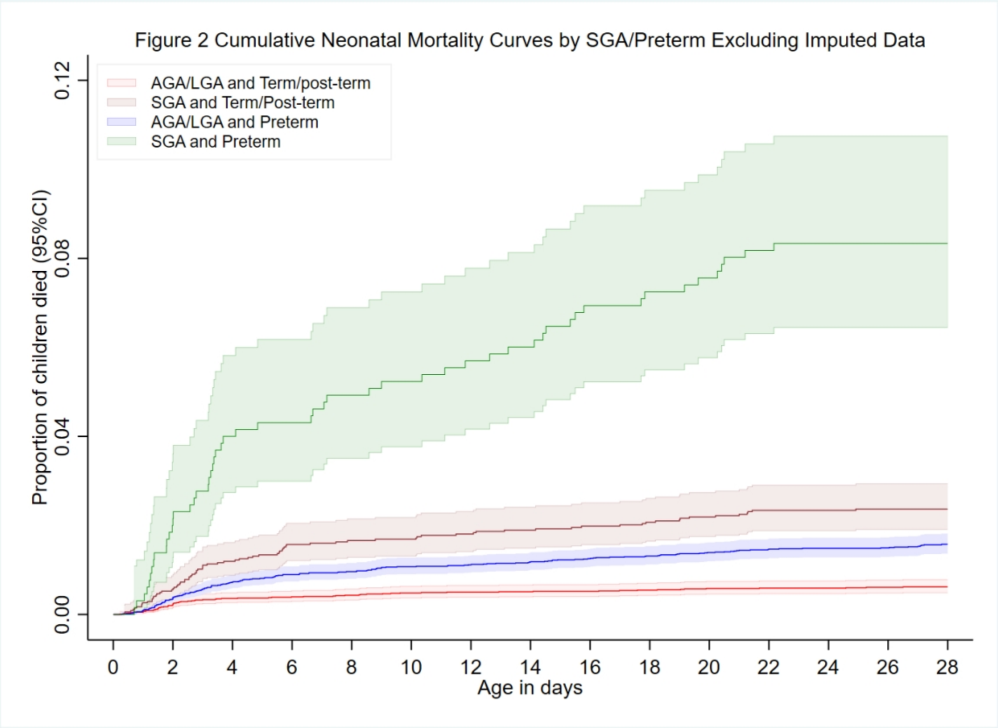

Figure 2 Cumulative Neonatal Mortality Curves by SGA/Preterm Excluding Imputed Data

489x355mm (72 x 72 DPI)

APPENDIX: SUPPLEMENTAL MATERIALS

Table S1 Multivariate Cox Regression Model S1

Variables	Crude Hazard Ratio	95% CI	**Model S1:** Maternal Age, Mother's Height, Mother's Years of Education, Wealth Quintile, Caste, Parity, Prior Child Deaths, Tetanus Vaccination, Place of Delivery, Number of ANC Visits, Sex of Child, Gestational Age, Size for Gestation, Singleton/Twin or Triplet			
			Adjusted Hazard Ratio (N=26,626)	95% CI	Adjusted HR (Using imputed SGA) (N=27,158)	95% CI
**Maternal Age (years) at LMP**						
Mean, 95% CI: 22.46 (22.41, 22.51)						
<=18	1.57	(1.34, 1.83)	1.34	(1.00, 1.76)	1.25	(1.02, 1.52)
18-35	1.00	Reference	1.00	Reference	1.00	Reference
>35	1.62	(1.14, 2.31)	1.39	(0.64, 3.04)	1.25	(0.74, 2.10)
**Maternal Height (cm)**						
Mean, 95% CI: 150.55 (150.49, 150.61)						
<145	1.00	Reference	1.00	Reference	1.00	Reference
145-150	0.70	(0.59, 0.83)	0.69	(0.52, 0.93)	0.82	(0.67, 1.00)
>=150	0.49	(0.41, 0.57)	0.63	(0.48, 0.84)	0.59	(0.49, 0.72)
**Maternal Education (years)**						
Mean, 95% CI: 2.61 (2.56, 2.65)						
0	1.00	Reference	1.00	Reference	1.00	Reference
1-5	0.81	(0.64, 1.03)	0.98	(0.67, 1.43)	0.95	(0.74, 1.24)
>5	0.64	(0.54, 0.76)	0.75	(0.53, 1.04)	0.77	(0.62, 0.97)
**Wealth Quintile**						
Poorest	1.00	Reference	1.00	Reference	1.00	Reference
Poorer	0.87	(0.72, 1.05)	0.92	(0.66, 1.27)	0.95	(0.76, 1.17)
Middle	0.80	(0.66, 0.97)	1.03	(0.74, 1.43)	1.00	(0.80, 1.25)
Richer	0.60	(0.49, 0.74)	0.82	(0.56, 1.19)	0.88	(0.69, 1.12)
Richest	0.59	(0.48, 0.73)	1.01	(0.68, 1.50)	1.00	(0.77, 1.31)
**Caste of the Family**						
Brahmin and Chhetri	1.00	Reference	1.00	Reference	1.00	Reference
Vaishya	1.10	(0.74, 1.63)	1.19	(0.53, 2.67)	0.86	(0.54, 1.37)
Shudra	1.57	(1.05, 2.37)	1.48	(0.63, 3.46)	1.00	(0.61, 1.64)
Muslim and others	1.01	(0.65, 1.57)	1.06	(0.44, 2.57)	0.73	(0.44, 1.23)
**Parity**						
1-4	1.00	Reference	1.00	Reference	1.00	Reference
>=5	1.75	(1.33, 2.30)	1.18	(0.65, 2.13)	1.02	(0.68, 1.53)
Prior pregnancy but no parity	1.75	(1.24, 2.47)	2.04	(0.45, 9.35)	1.50	(0.65, 3.46)
No prior pregnancy	1.58	(1.38, 1.82)	1.38	(1.02, 1.85)	1.68	(1.29, 1.93)
**Prior child death**						
No prior pregnancy	1.76	(1.52, 2.04)	Omitted		Omitted	
Prior live births but no deaths	1.00	Reference	1.00	Reference	1.49	(0.67, 3.29)
Prior live births and child death	1.80	(1.48, 2.18)	1.14	(0.82, 1.58)	1.00	Reference
Prior pregnancy but no live birth	1.83	(1.33, 2.53)	0.75	(0.18, 3.09)	1.55	(1.26, 1.91)
**Tetanus Vaccination in past 2 years**						
No	1.00	Reference	1.00	Reference	1.00	Reference
Yes	0.78	(0.66, 0.92)	0.84	(0.64, 1.11)	0.81	(0.67, 0.96)
**Place of Delivery**						
At home or Maiti*	1.00	Reference	1.00	Reference	1.00	Reference
At health post/clinic or in hospital	1.22	(1.06, 1.40)	0.92	(0.71, 1.18)	1.30	(1.10, 1.54)
On the way to facility or outdoors	2.43	(1.70, 3.48)	1.20	(0.66, 2.17)	2.08	(1.47, 2.95)
**Number of Antenatal Care (ANC)**						
0	1.00	Reference	1.00	Reference	1.00	Reference
1	1.07	(0.86, 1.34)	0.99	(0.70, 1.39)	1.05	(0.84, 1.31)
2 or 3	1.12	(0.93, 1.35)	1.04	(0.78, 1.39)	1.10	(0.91, 1.34)
>=4	0.61	(0.50, 0.76)	0.77	(0.54, 1.10)	0.70	(0.55, 0.90)
**Sex of Child**						
Male	1.00	Reference	1.00	Reference	1.00	Reference
Female	0.88	(0.77, 0.99)	1.16	(0.94, 1.43)	0.91	(0.80, 1.05)
**Gestational Age**						
Very preterm (<32 weeks)	17.44	(14.54, 20.91)	45.45	(27.63, 74.75)	20.7	(13.88, 30.86)
Moderate to late preterm (32-37 weeks)	2.66	(2.25, 3.14)	2.85	(2.11, 3.85)	2.59	(2.07, 3.25)
Term (37-42 weeks)	1.00	Reference	1.00	Reference	1.00	Reference
Post-term (>= 42 weeks)	1.42	(1.16, 1.75)	1.14	(0.82, 1.59)	1.25	(0.98, 1.59)
**Size for Gestational Age (within 72 hours)**						
AGA	1.00	Reference	1.00	Reference		
SGA	1.76	(1.42, 2.18)	2.30	(1.77, 3.00)		
LGA	0.63	(0.35, 1.12)	0.09	(0.04, 0.19)		
**Imputed Size for Gestational Age**						
AGA	1.00	Reference			1.00	Reference
SGA	1.43	(1.10, 1.87)			1.66	(1.10, 2.50)
LGA	4.52	(3.49, 5.85)			0.75	(0.52, 1.10)
**Singleton/Twin/Triplet**						
Singleton	1.00	Reference	1.00	Reference	1.00	Reference
Twin or Triplet	8.31	(6.43, 10.74)	3.26	(2.04, 5.21)	3.77	(2.75, 5.17)

* Maiti is the maternal home, where women may go to deliver, especially in the first pregnancy

For peer review only

Table S2 Multivariate Cox Regression Model S2

Variables	Crude Hazard Ratio	95% CI	**Model S2:** Maternal Age, Mother's Height, Mother's Years of Education, Wealth Quintile, Caste, Parity, Prior Child Death, Tetanus Vaccination, Sex of Child, Gestational Age, Size for Gestation, Singleton/Twin or Triplet			
			**Adjusted Hazard Ratio (N=26,680)**	**95% CI**	**Adjusted HR (Using imputed SGA) (N=30,332)**	**95% CI**
**Maternal Age (years) at LMP**						
Mean, 95% CI: 22.46 (22.41, 22.51)						
<=18	1.57	(1.34, 1.83)	1.35	(1.02, 1.80)	1.15	(0.96, 1.39)
18-35	1.00	Reference	1.00	Reference	1.00	Reference
>35	1.62	(1.14, 2.31)	1.36	(0.63, 2.93)	1.32	(0.82, 2.15)
**Maternal Height (cm)**						
Mean, 95% CI: 150.55 (150.49, 150.61)						
<145	1.00	Reference	1.00	Reference	1.00	Reference
145-150	0.70	(0.59, 0.83)	0.69	(0.51, 0.93)	0.83	(0.69, 1.01)
>=150	0.49	(0.41, 0.57)	0.63	(0.48, 0.84)	0.60	(0.50, 0.72)
**Maternal education (years)**						
Mean, 95% CI: 2.61 (2.56, 2.65)						
No schooling	1.00	Reference	1.00	Reference	1.00	Reference
1-5	0.81	(0.64, 1.03)	0.95	(0.65, 1.39)	0.92	(0.72, 1.17)
>5	0.64	(0.54, 0.76)	0.71	(0.51, 0.99)	0.75	(0.61, 0.93)
**Wealth Quintile**						
Poorest	1.00	Reference	1.00	Reference	1.00	Reference
Poorer	0.87	(0.72, 1.05)	0.91	(0.66, 1.25)	0.99	(0.81, 1.22)
Middle	0.80	(0.66, 0.97)	1.00	(0.72, 1.39)	0.99	(0.80, 1.22)
Richer	0.60	(0.49, 0.74)	0.79	(0.54, 1.15)	0.88	(0.70, 1.10)
Richest	0.59	(0.48, 0.73)	0.99	(0.67, 1.46)	0.99	(0.78, 1.27)
**Caste of the Family**						
Brahmin and Chhetri	1.00	Reference	1.00	Reference	1.00	Reference
Vaishya	1.10	(0.74, 1.63)	1.23	(0.54, 2.77)	0.88	(0.57, 1.34)
Shudra	1.57	(1.05, 2.37)	1.54	(0.66, 3.60)	0.99	(0.62, 1.56)
Muslim	1.01	(0.65, 1.57)	1.10	(0.45, 2.65)	0.70	(0.43, 1.13)
**Parity**						
1-4	1.00	Reference	1.00	Reference	1.00	Reference
>=5	1.75	(1.33, 2.30)	1.19	(0.66, 2.15)	1.03	(0.70, 1.51)
Prior pregnancy but no parity	1.75	(1.24, 2.47)	2.08	(0.45, 9.57)	1.38	(0.65, 2.93)
No prior pregnancy	1.58	(1.38, 1.82)	1.34	(1.01, 1.77)	1.65	(1.38, 1.98)
**Prior child deaths**						
No prior pregnancy	1.76	(1.52, 2.04)	Omitted		Omitted	
Prior live births but no deaths	1.00	Reference	1.00	Reference	1.00	Reference
Prior live births and child death	1.80	(1.48, 2.18)	1.14	(0.82, 1.59)	1.49	(1.22, 1.82)
Prior pregnancy but no live birth	1.83	(1.33, 2.53)	0.69	(0.17, 2.87)	1.54	(0.76, 3.11)
**Tetanus Vaccination in past 2 years**						
No	1.00	Reference	1.00	Reference	1.00	Reference
Yes	0.78	(0.66, 0.92)	0.84	(0.64, 1.10)	0.94	(0.79, 1.11)
**Sex of Child**						
Male	1.00	Reference	1.00	Reference	1.00	Reference
Female	0.88	(0.77, 0.99)	1.18	(0.96, 1.46)	0.89	(0.78, 1.01)
**Gestational Age**						
Very preterm (<32 weeks)	17.44	(14.54, 20.91)	47.53	(28.85, 78.31)	22.57	(15.95, 31.95)
Moderate to late preterm (32-37 weeks)	2.66	(2.25, 3.14)	2.92	(2.16, 3.94)	2.70	(2.21, 3.30)
Term (37-42 weeks)	1.00	Reference	1.00	Reference	1.00	Reference
Post-term (42-45 weeks)	1.42	(1.16, 1.75)	1.13	(0.81, 1.58)	1.25	(1.00, 1.57)
**Size for Gestational Age (within 72 hours)**						
AGA	1.00	Reference	1.00	Reference		
SGA	1.76	(1.42, 2.18)	2.31	(1.78, 3.00)		
LGA	0.63	(0.35, 1.12)	0.09	(0.04, 0.19)		
**Imputed Size for Gestational Age**						
AGA	1.00	Reference			1.00	Reference
SGA	1.43	(1.10, 1.87)			1.66	(1.18, 2.34)
LGA	4.52	(3.49, 5.85)			0.76	(0.52, 1.10)
**Singleton/Twin/Triplet**						
Singleton	1.00	Reference	1.00	Reference	1.00	Reference
Twin or Triplet	8.31	(6.43, 10.74)	3.15	(1.98, 5.04)	3.97	(2.97, 5.30)

For peer review only

STROBE Statement—checklist of items that should be included in reports of observational studies

	Item No	Recommendation	Page No
**Title and abstract**	1	(*a*) Indicate the study's design with a commonly used term in the title or the abstract	1
		(*b*) Provide in the abstract an informative and balanced summary of what was done and what was found	2-3
**Introduction**			
Background/rationale	2	Explain the scientific background and rationale for the investigation being reported	4
Objectives	3	State specific objectives, including any prespecified hypotheses	4
**Methods**			
Study design	4	Present key elements of study design early in the paper	4-5
Setting	5	Describe the setting, locations, and relevant dates, including periods of recruitment, exposure, follow-up, and data collection	4-5, Figure1
Participants	6	(*a*) *Cohort study*—Give the eligibility criteria, and the sources and methods of selection of participants. Describe methods of follow-up<br>*Case-control study*—Give the eligibility criteria, and the sources and methods of case ascertainment and control selection. Give the rationale for the choice of cases and controls<br>*Cross-sectional study*—Give the eligibility criteria, and the sources and methods of selection of participants	4-5
		(*b*) *Cohort study*—For matched studies, give matching criteria and number of exposed and unexposed<br>*Case-control study*—For matched studies, give matching criteria and the number of controls per case	
Variables	7	Clearly define all outcomes, exposures, predictors, potential confounders, and effect modifiers. Give diagnostic criteria, if applicable	5-7
Data sources/ measurement	8*	For each variable of interest, give sources of data and details of methods of assessment (measurement). Describe comparability of assessment methods if there is more than one group	5-8
Bias	9	Describe any efforts to address potential sources of bias	6-7, 14-15
Study size	10	Explain how the study size was arrived at	5, Figure1
Quantitative variables	11	Explain how quantitative variables were handled in the analyses. If applicable, describe which groupings were chosen and why	5-7
Statistical methods	12	(*a*) Describe all statistical methods, including those used to control for confounding	5-7,13-14
		(*b*) Describe any methods used to examine subgroups and interactions	5-7,13-14
		(*c*) Explain how missing data were addressed	5-7,13-14
		(*d*) *Cohort study*—If applicable, explain how loss to follow-up was addressed<br>*Case-control study*—If applicable, explain how matching of cases and controls was addressed<br>*Cross-sectional study*—If applicable, describe analytical methods taking account of sampling strategy	5-7, Figure1
		(*e*) Describe any sensitivity analyses	14

Continued on next page

For peer review only - http://bmjopen.bmj.com/site/about/guidelines.xhtml

Results

Participants	13*	(a) Report numbers of individuals at each stage of study—eg numbers potentially eligible, examined for eligibility, confirmed eligible, included in the study, completing follow-up, and analysed	7-10, Figure 1
		(b) Give reasons for non-participation at each stage	Figure 1
		(c) Consider use of a flow diagram	Figure 1
Descriptive data	14*	(a) Give characteristics of study participants (eg demographic, clinical, social) and information on exposures and potential confounders	8-10
		(b) Indicate number of participants with missing data for each variable of interest	8-10
		(c) *Cohort study*—Summarise follow-up time (eg, average and total amount)	
Outcome data	15*	*Cohort study*—Report numbers of outcome events or summary measures over time	
		Case-control study—Report numbers in each exposure category, or summary measures of exposure	
		Cross-sectional study—Report numbers of outcome events or summary measures	8-13
Main results	16	(*a*) Give unadjusted estimates and, if applicable, confounder-adjusted estimates and their precision (eg, 95% confidence interval). Make clear which confounders were adjusted for and why they were included	8-14
		(*b*) Report category boundaries when continuous variables were categorized	8-12
		(*c*) If relevant, consider translating estimates of relative risk into absolute risk for a meaningful time period	
Other analyses	17	Report other analyses done—eg analyses of subgroups and interactions, and sensitivity analyses	12-14

Discussion

Key results	18	Summarise key results with reference to study objectives	14-15
Limitations	19	Discuss limitations of the study, taking into account sources of potential bias or imprecision. Discuss both direction and magnitude of any potential bias	14-15
Interpretation	20	Give a cautious overall interpretation of results considering objectives, limitations, multiplicity of analyses, results from similar studies, and other relevant evidence	14-16
Generalisability	21	Discuss the generalisability (external validity) of the study results	14-15

Other information

Funding	22	Give the source of funding and the role of the funders for the present study and, if applicable, for the original study on which the present article is based	15-16

*Give information separately for cases and controls in case-control studies and, if applicable, for exposed and unexposed groups in cohort and cross-sectional studies.

Note: An Explanation and Elaboration article discusses each checklist item and gives methodological background and published examples of transparent reporting. The STROBE checklist is best used in conjunction with this article (freely available on the Web sites of PLoS Medicine at http://www.plosmedicine.org/, Annals of Internal Medicine at http://www.annals.org/, and Epidemiology at http://www.epidem.com/). Information on the STROBE Initiative is available at www.strobe-statement.org.

For peer review only - http://bmjopen.bmj.com/site/about/guidelines.xhtml
For peer review only
